# Supplementary figures and images for: A realist evaluation of the development, implementation and outcomes of the first public ART Centre in Morocco
Source: PLOS Glob Public Health. 2026 Apr 20;6(4):e0005318. doi: 10.1371/journal.pgph.0005318 (PMC13094999; doi:10.1371/journal.pgph.0005318)

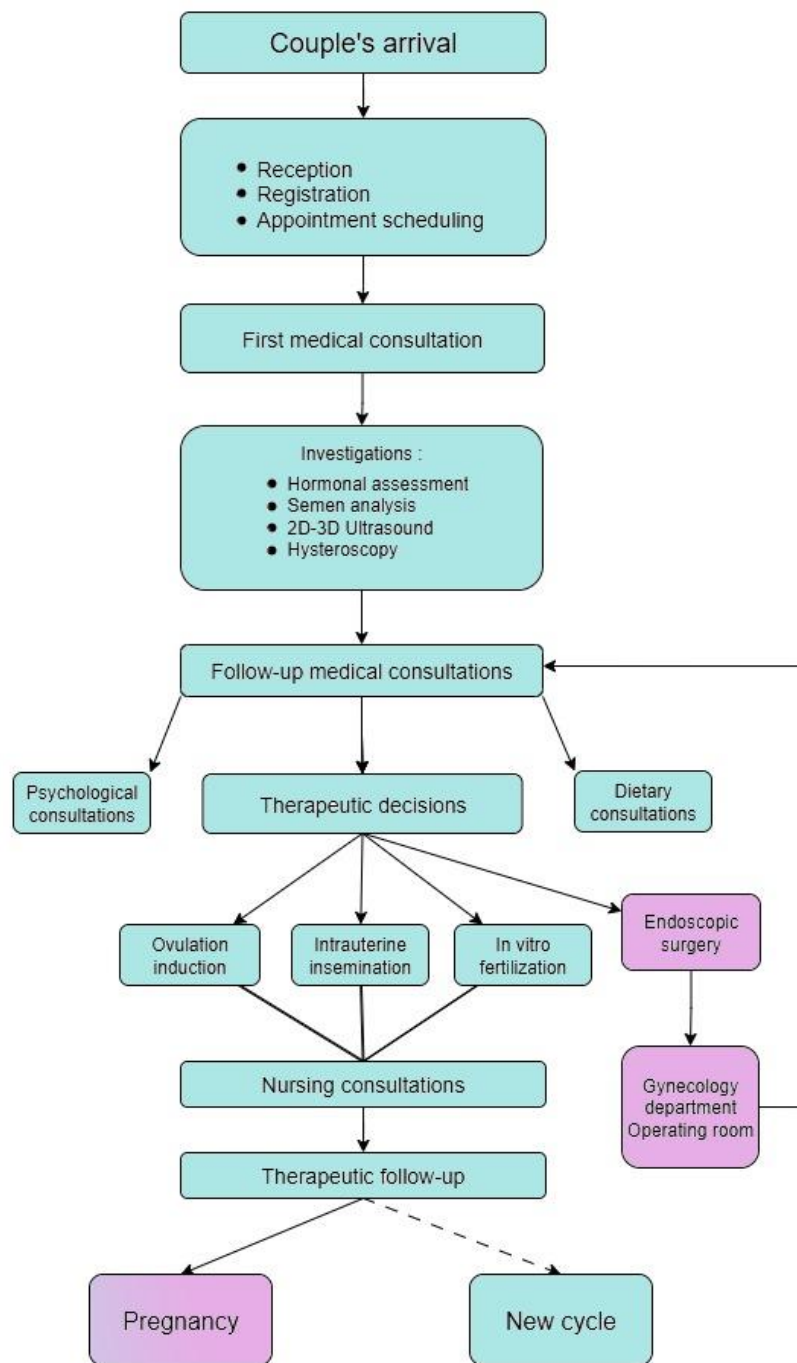

**Couples' care pathway**

Supplement: S1 Fig — (PDF) [file pgph.0005318.s001.pdf]
