# Supplementary material for: A realist evaluation of the development, implementation and outcomes of the first public ART Centre in Morocco
Source: PLOS Glob Public Health. 2026 Apr 20;6(4):e0005318. doi: 10.1371/journal.pgph.0005318 (PMC13094999; doi:10.1371/journal.pgph.0005318)
Supplement: S2 Table — (PDF) [file pgph.0005318.s006.pdf]

Characteristics of the centre's population

	**Women**	**Men**
**Age (mean ± SD)**	33.1 ± 5.5	40.7 ± 7.25
**Profession n (%)**		
Housewife / no profession	1875 (75.2%)	115 (4.6%)
Civil servant	221 (8.9%)	329 (13.2%)
Police, army, security	20 (0.8%)	304 (12.2%)
Merchant	15 0.6%	259 (10.4%)
Worker	59 (2.4%)	808 (32.4%)
Employee	166 (6.7%)	370 (14.8%)
Private executive	66 (2.6%)	99 (4%)
Farmer	1 (0.0%)	33 (1.3%)
Craftswoman / Craftsman	60 (2.4%)	142 (5.7%)
Other professions	12 (0.5%)	36 (1.4%)
**Region of residence (%)**		
Tanger-Tetouan-AlHoceima	152 (6.1%)	
Oriental	64 (2.6%)	
Fez-Meknes	170 (6.8%)	
Rabat-Sale-Kenitra	1649 (66.1%)	
Beni Mellal-Khenifra	50 (2%)	
Settat-Casablanca	190 (7.6%)	
Marrakech-Safi	66 (2.6%)	
Drâa-Tafilalt	63 (2.5%)	
Souss-Massa	40 (1.6%)	
Guelmim-Oued Noun	14 (0.6%)	
Laâayoun-Sakia El Hamra	16 (0.6%)	
Dakhla-Oued Eddahab	6 (0.2%)	
Other countries	15 (0.6%)	
**Infertility Duration (mean ± SD)**	6.37 ± 4.32	
**Infertility type n (%)**		
Primary infertility	1961 (78.6%)	
Secondary infertility	534 (21.4%)	
**Prior treatments n (%)**	**52.9%**	

Ovulation induction	926 (37.1%)
IUI	195 (7.8%)
IVF	199(8%)
**Diagnosis n (%)**	
**Ovulatory disorders**	615 (24.6%)
Polycystic Ovary Syndrome	273 (10.9%)
Reduced ovarian reserve	253 (10.1%)
Hyperprolactinemia	50 (2%)
Premature Ovarian Failure	32 (1.3%)
Hypogonadotropic Hypogonadism	7 (0.3%)
**Tubal factor**	632 (25.3%)
**Acquired Uterine abnormalities**	376 (15%)
**Congenital Uterine abnormalities**	75 (3%)
**Endometriosis**	78 (3.1%)
**Endocrine causes**	24 (1%)
**Male factor**	1195 (47.9%)
Oligoasthenoteratospermia	695 (27.9%)
Azoospermia	485 (19.4%)
Anejaculation	5 (0.2%)
Erectile dysfunction	5 (0.2%)
Retrograde ejaculation	5 (0.2%)
No sperm	10 (0.4%)
