# Supplementary material for: A realist evaluation of the development, implementation and outcomes of the first public ART Centre in Morocco
Source: PLOS Glob Public Health. 2026 Apr 20;6(4):e0005318. doi: 10.1371/journal.pgph.0005318 (PMC13094999; doi:10.1371/journal.pgph.0005318)
Supplement: S3 Table — (PDF) [file pgph.0005318.s007.pdf]

Timeline of the development and implementation of the first public ART centre in Morocco

Period	Project milestones	Details
**2008**	Project design	• ART centre project conceived, with the vision of establishing a public infertility service in Morocco
**2009-2010**	Team training (Phase 1)	• Multidisciplinary medical staff (gynaecologists, biologist) begin specialized ART training in Belgium (Free University of Brussels, Erasme Hospital, University of Liège, La Citadelle Hospital).<br>• Training lasted 12 months.
**2010-2011**	Team training (Phase 2)	• Multidisciplinary medical and paramedical staff (cytogenetic doctor, lab technicians, Head nurse) begin specialized ART training in Belgium<br>• Training lasted 3 months (nurse), 6 months (lab technicians), or 12 months (cytogenetic doctor) depending on profile.<br>• On-site training of the nursing team at the ART centre.
**2010-2013**	Infrastructure development	• Construction of a dedicated ART building at HMSRO financed by Ibn Sina University Hospital.<br>• Conducted in 2 phases (Nov 2010–Dec 2013).
**2011-2013**	Equipment procurement	• Purchasing, delivery, and installation of medical and non-medical equipment through CHUIS procedures.
**2012-2013**	Consumables and drugs	• Procurement of drugs and consumables according to CHUIS procedures<br>• Initial procurement of culture media and ART-specific devices was funded by Belgian budget<br>• Regulatory delays in registration temporarily hindered procurement via CHUIS budget.
	Protocols, procedures and adaptation to Moroccan context	• Clinical, laboratory, and nursing protocols co-developed with Belgian partners ;<br>• Manual of procedures finalized;<br>• Working procedures established with other CHUIS departments (pharmacy, laboratory, biomedical, billing).<br>• Eligibility, and counselling adapted to address legal, religious and social framework.
	Health Information System	• Gyneco 2000 software implemented for couples records and data monitoring
	Financing	• Pricing of ART procedures developed with CHUIS;<br>• Bundled fee system introduced for IUI, IVF, FET, and ovulation induction ;<br>• Advocacy to Ministry of Health for drug reimbursement and ART inclusion in insurance

		schemes.
**Oct 2013**	**Start of clinical activity**	• Gradual rollout of services:<br>• 2013 : Ovulation Induction<br>• 2014 : IUI<br>• 2015 : IVF<br>• 2017 : FET
**2013-2015**	Public communication & awareness	• Information campaign via CHUIS journal, staff updates, scientific meetings, and outreach to general practitioners and specialists.
**May 2016**	**Official inauguration**	• ART centre formally inaugurated and recognized by government officials;<br>• Extensive media coverage increased referrals and demand.
**2009-2022**	Governance & service consolidation	• Governance anchored within CHUIS and Mohammed V University, with Ministry of Health oversight;<br>• Coordination ensured through regular meetings, performance reviews, financial reporting, and service delivery monitoring.
**2013-2022**	Service uptake	• 2,495 couples received infertility services ;<br>• with discontinuous service provision due to delays in regulatory procedures for registration of ART supplies.
