# Supplementary material for: A realist evaluation of the development, implementation and outcomes of the first public ART Centre in Morocco
Source: PLOS Glob Public Health. 2026 Apr 20;6(4):e0005318. doi: 10.1371/journal.pgph.0005318 (PMC13094999; doi:10.1371/journal.pgph.0005318)
Supplement: S4 Table — (PDF) [file pgph.0005318.s008.pdf]

Cost of ART cycles at the centre

Treatment	Cost	Acts included in the package
**IVF**	13,910 MAD<br>(1,535 USD)	-Ovarian stimulation drugs (GnRH Agonist, GnRH Antagonist, Gonadotrophines)<br>-Hormonal and Ultrasound monitoring<br>-Oocyte pick up including anesthesia<br>-Laboratory acts til embryo transfer and / or vitrification<br>-Embryo transfer<br>-Embryo Cryopreservation for 5 years<br>-HCG dosage at L14<br>-Early pregnancy Ultasound at 6-7weeks
**FET**	3,746 MAD<br>(414 USD)	-Hormonal and Ultrasound monitoring<br>-Embryo thawing<br>-Embryo transfer
**IUI**	3,100 MAD<br>(342 USD)	-Hormonal and Ultrasound monitoring<br>-Spermo preparation<br>-Intra Uterine Insemination<br>-HCG dosage at L14<br>-Early pregnancy Ultasound at 6-7weeks
**Ovulation induction**	2,000 MAD<br>(221 USD)	-Ovarian stimulation drugs (Gonadotrophines)<br>-Hormonal and Ultrasound monitoring

*MAD: Moroccan dirhams, USD: US dollars
