# Supplementary material for: A realist evaluation of the development, implementation and outcomes of the first public ART Centre in Morocco
Source: PLOS Glob Public Health. 2026 Apr 20;6(4):e0005318. doi: 10.1371/journal.pgph.0005318 (PMC13094999; doi:10.1371/journal.pgph.0005318)
Supplement: S5 Table — (PDF) [file pgph.0005318.s009.pdf]

Characteristics of Fresh IVF cycles

**Own fresh IVF cycle (N)**	**236**
Initiated n/N (%)	236 (100%)
Cancelled n/N (%)	20 (8.5%)
Oocyte pick up (OPU) n/N (%)	216 (91.5%)
Embryo Transfer n/N (%)	151 (64%)
**Causes of cycle cancellation** n/N (%)	
No ovarian response	11 (4.7%)
OHSS risk	5 (2.1%)
No medical reason	4 (1.7%)
**Pituitary inhibition n/N (%)**	
Antagonist protocol	131 (55.5%)
Long Agonist protocol	17 (7.2%)
Short Agonist protocol	88 (37.3%)
**Ovarian stimulation n/N (%)**	
Stimulation with gonadotropins	236 (100%)
Total dose of gonadotropins (ui) (median, IC)	2700 [Q1 : 1838, Q3 : 3300]
**Ovulation Trigger n/N (%)**	
HCG	173 (73.3%)
GnRH Agonist	44 (18.6%)
**Number of Oocytes retrieved** (median, IC)	9 [Q1 : 5, Q3 : 13]
2 PN oocytes (median, IC)	4 [Q1 : 2, Q3 : 8]
**Complication rate after OPU**	0
**Number of embryos transferred (%)**	
One embryo	55/151 (36.4%)
Two embryos	96/151 (63.6%)
**Cause of No transfer n/N (%)**	65 (27.5%)
No oocytes	3 (1.3%)

Polyspermic fertilization	1 (0.4%)
Fertilization failure	13 (5.5%)
OHSS risk	33 (14%)
Poor embryo quality	5 (2.1%)
Premature progesterone elevation	1 (0.4%)
Other medical reasons	8 (3.4%)
No medical reason	1 (0.4%)
**Cryopreservation rate n/N (%)**	140 (59.3%)
**Clinical pregnancy rate /transfer**	36/151 (23.8%)
**Live Birth rate / transfer**	25/151 (16.6%)
**Multiple pregnancy rate**	5/36 (13.9%)
**Pregnancy loss**	
Miscarriage Rate	10/36 (27.8%)
Ectopic pregnancy	1 /36 (2.8%)
**Mode of delivery**	25 deliveries
C section	21/25 (84%)
Vaginal	4/25 (16%)
**Gender of the baby**	
Male	11
Female	13
**Birth weight (g)** (median, IC)	3200 [Q1: 2750 Q3: 3425]

*OHSS : Ovarian Hyperstimulation Syndrome
