# Supplementary material for: A realist evaluation of the development, implementation and outcomes of the first public ART Centre in Morocco
Source: PLOS Glob Public Health. 2026 Apr 20;6(4):e0005318. doi: 10.1371/journal.pgph.0005318 (PMC13094999; doi:10.1371/journal.pgph.0005318)
Supplement: S6 Table — (PDF) [file pgph.0005318.s010.pdf]

Characteristics of FET cycles

**Own FET cycle (N)**	**121**
Initiated n/N (%)	121 (100%)
Cancelled n/N (%)	16 (13.2%)
Embryo Transfer n/N (%)	105 (86.8%)
**Causes of cycle cancellation n/N (%)**	
Immature endometrium	3 (2.5%)
Other medical reason	3 (2.5%)
No medical reason	10 (8.3%)
**Endometrial preparation n/N (%)**	
Hormone replacement therapy	119 (98.3%)
Modified natural cycle	2 (1.7%)
**Number of embryos transferred (%)**	
One embryo	34 /105 (32.4%)
Two embryos	71 /105 (67.6%)
**Clinical pregnancy rate / transfer**	22 /105 (21%)
**Live Birth rate / transfer**	15/105 (14.3%)
**Multiple pregnancy rate**	4 /22 (18.2%)
**Pregnancy Loss**	7 /22 (31.8%)
Miscarriage Rate	6 /22 (27.3%)
Maternal death at 24 weeks (pulmonary embolism related to COVID-19)	1 / 22 (4.5%)
**Mode of delivery**	15 deliveries
C section	10 /15 (66.7%)
Vaginal	5 /15 (33.3%)

**Gender of the newborn**	
Male	8 /15 (53.3%)
Female	7 /15 (46.7%)
**Birth weight (g)** (mean ± SD)	2999 ± 972
**Early neonatal death rate (%)**	2 /15 (13.3%)
