# Supplementary material for: A realist evaluation of the development, implementation and outcomes of the first public ART Centre in Morocco
Source: PLOS Glob Public Health. 2026 Apr 20;6(4):e0005318. doi: 10.1371/journal.pgph.0005318 (PMC13094999; doi:10.1371/journal.pgph.0005318)
Supplement: S7 Table — (PDF) [file pgph.0005318.s011.pdf]

Characteristics of Ovulation induction cycles

**Ovulation induction cycle (N)**	**305**
Initiated n/N (%)	305 (100%)
Cancelled n/N (%)	73 /305 (23.9%)
**Stimulation protocol n/N (%)**	
Stimulation with clomiphene	58 (19%)
Stimulation with gonadotropins	247 (81%)
Total dose of gonadotropins administered (ui) (mean ± SD)	974.6 ± 684.2
**Ovulation Trigger n/N (%)**	
HCG	199 (65.2%)
Spontaneous LH surge	33 (10.8%)
**Cause of cancellation n/N (%)**	
No ovarian response	33 (10.8%)
Multiple pregnancy risk	20 (6.6%)
OHSS risk	2 (0.7%)
Other medical reason	4 (1.3%)
No medical reason	14 (4.6%)
**Clinical pregnancies rate /cycle (without cancelled cycles) (%)**	42 /232 (18.1%)
**Live birth rate /cycle (without cancelled cycles) (%)**	29 /232 (12.5%)
**Multiple pregnancy rate**	3 / 42 (7.14%)
**Pregnancy Loss**	
Miscarriage Rate	12 /42 (28.6%)
Ectopic pregnancy	1 /42 (0.3%)
**Mode of delivery**	29 deliveries

C section	12 (41.4%)
Vaginal	17 (58.6%)
**Gender of the baby**	
Male	10 (34.5%)
Female	19 (65.5%)
**Birth weight (g)** (median, IC)	3200 [Q1:2950 Q3 : 3560]
**Early neonatal death rate (%)**	2 /29 (6.9%)

*OHSS : Ovarian Hyperstimulation Syndrome

Characteristics of IUI cycles

**Intrauterine insemination cycles N**	**29**
Initiated n/N (%)	29 (100%)
Cancelled n/N (%)	2 /29 (6.9%)
**Stimulation protocol n/N (%)**	
Stimulation with clomiphene	15 (51.7%)
Stimulation with gonadotropins	14 (48.3%)
Total dose of gonadotrophins administered (ui) (median, IC)	525 [Q1 :500 , Q3 :750]
**Ovulation Trigger**	HCG
**Cause of cancellation**	Other medical reason
**Clinical pregnancies n/N (%)**	1 (3.7%)
**Gender of the baby**	Female
**Birth weight (g)**	3200
**Number of newborns n/N (%)**	Singleton
