# Supplementary material for: A realist evaluation of the development, implementation and outcomes of the first public ART Centre in Morocco
Source: PLOS Glob Public Health. 2026 Apr 20;6(4):e0005318. doi: 10.1371/journal.pgph.0005318 (PMC13094999; doi:10.1371/journal.pgph.0005318)
Supplement: S2 Data — (ZIP) [file pgph.0005318.s013.zip › S2_Data_Transcriptions_in _English/C10.pdf]

Interview for Men and Women with Infertility

Participant Code NUMBER: _________C10

2. Experience with infertility prior to coming to this ART Center
Now, I would like to ask you a few questions about your experience with infertility before you came to this center.

Being an infertile couple in Morocco means being subjected to all kinds of stigmatization, especially for the woman. Society considers you disabled, incomplete, the target of discussion. It's a source of social isolation, constantly traumatized at every encounter with family or friends by questions about pregnancy and the reasons for delays, and the forced proposals for remedies, treatments, doctors, and recipes, without any respect for my privacy or intimacy. I decided to no longer participate in or respond to invitations. I prefer to remain in my quiet corner; society is difficult and people are too aggressive. I have suffered from depression for a long time.

3. Help seeking and first impressions
We saw the center's inauguration on television and thought, why not? It's an opportunity we shouldn't miss, especially since the price offered by the center is significantly lower than that of private clinics. Private clinics make IVF impossible for us because of the exorbitant prices that far exceed our financial means, with a considerable risk of failure.

4. Experiences of accessing care at the ART Center
In all honesty, the center's team surprised us with their professionalism, presence, and empathy. We've never had such a relationship with healthcare professionals in the private sector, unfortunately. Listening and trust play a crucial role in infertility treatment; infertile couples are deeply traumatized and psychologically and financially vulnerable. At the center, I feel they take this into account, and it influences the results.

4.3. Are you satisfied with the quality of your care at this public center:

• Information : YES

• Communication: YES

• Health professionnal support : YES

• Medical care: YES

• Financial accessibility : YES

4.4. Was the nursing consultation beneficial for you?

Yes

4.5. Why?

Even though we had undergone treatment several times before arriving at the center, I think the discussion with the nurse was very helpful. Her explanations were very simple and appropriate. She listened and answered our questions.

The checks for understanding, the fluidity of communication, and the psychological support—these elements marked my experience at the center, which, thankfully, was a success with the arrival of my little one.

4.6. Have you at any point in time considered stopping treatment from this center? Why?

No, never, despite all the suffering, did I cling to the hope of having a baby.

4.7. How much money have you already spent on diagnosis and treatment? Where did you obtain those funds from? What helped you to cope with the financial pressures?

We have spent millions on consultations, tests, and medications, which remain too expensive and are not reimbursed. We have repeatedly had to stop follow-up with our primary care physician because of the expense of all our money and the need to seek other sources to start again—selling assets or borrowing from a relative.

5. Benefits of a public ART Center

5.1. Had you attended a private clinic prior to coming to this ART center?

Yes

Before arriving at the center, we consulted several doctors and private clinics. Unfortunately, the private sector's interest is purely financial; we didn't support each other. That's why I kept changing doctors, and when a woman suggested a doctor to me...

However, at the center, we feel well cared for, with special attention given to each couple and close follow-up by phone and email. We feel important and that our problem matters to the team.

In addition, the cost of care is affordable, and the center's doctor is truly very empathetic and attentive.

I recommend the center to any woman suffering from this problem. Because here, she will receive the correct information and appropriate, high-quality care.

Thank you very much, that is the end of the interview. I will stop the recording now
