# Supplementary material for: A realist evaluation of the development, implementation and outcomes of the first public ART Centre in Morocco
Source: PLOS Glob Public Health. 2026 Apr 20;6(4):e0005318. doi: 10.1371/journal.pgph.0005318 (PMC13094999; doi:10.1371/journal.pgph.0005318)
Supplement: S2 Data — (ZIP) [file pgph.0005318.s013.zip › S2_Data_Transcriptions_in _English/C11.pdf]

Interviewor Men and Women with Infertility

Participant Code NUMBER: ___________C11

1. Do you have health insurance? Have you completed the CNSS procedures? What was the reason for the refusal?

Man said:

No, when I went to the administration to apply for social security because I had health coverage, RAMED. The financial officer refused to enroll me in social security, even though I met all the requirements, claiming that he hadn't received adequate instructions from the relevant authorities. Thank you, Madam, for raising this issue, since the state has given us this opportunity, but we encounter disruptive obstacles. The majority of people don't have the means to pay the treatment fees. It is absolutely essential that infertility be included as a disease covered by social security. It is a silent suffering!

3. When did you get married? Before coming to the center, had you already seen a doctor? Man said:

We went to see many doctors in Beni Mellal, Khouribga, and Oued Zem until people recommended this center to me.

4. How many doctors did you visit? The first doctor? What was his diagnosis?

Wife said:

No, I don't remember.

Man said:

The first doctor was in Oued Zem, and the treatment lasted a year without a diagnosis of my wife's condition: hormonal dysfunction and a lack of ovarian reserve. After some time, we saw another doctor in Beni Mellal, who also stayed for a year, then we came to Rabat.

5. What type of medication? Was it effective? Did you take any other medications like injections?

Wife said:

Just the tablets, but without any results, until I decided to change doctors again.

Man said:

We felt frustrated and decided to change doctors again.

6. How did you come to this center? Who recommended it to you?

Man said:

A friend told me about this center.

7. How did you make an appointment?

Man said:

The first time I came to the center to make an appointment, and about a year later, I was contacted. Now, six months of treatment have passed, and these are the final stages.

8. How have you experienced these years of treatment psychologically? How does the community perceive infertility?

Man says:

Personally, I don't see late pregnancy as a problem in my life, and it doesn't hurt me as much as it does my wife. Since the majority of society is unaware of these sophisticated and modern ways to help couples have children, I decided we wouldn't discuss this process with anyone to avoid any embarrassment or overwhelming questions, the first of which will be how do you know it's your baby?

Wife says:

As a woman, I feel embarrassed when people ask me why my pregnancy has been delayed until now. The problem is that people who aren't close to me ask me questions, regardless of their intentions, while my relatives and my husband have never asked me about it. It's God's will.

9. How did you manage financially during this time?

Man said:

As I said earlier, it's a long process, six years of treatment, six years of medication. We would stop from time to time until we could save some money or borrow it.

10. In your opinion, how can the state help?

Man said:

The state can help us with health coverage and by creating many centers in many cities, like in the rest of the country. In our case, we discovered this center after a long struggle with treatment because the prevalence of information about this center is very low, which leads many people to seek treatment in private clinics despite the cost.

In Oued Zem, a clinic was opened, and despite the cost, most people prefer treatment there because of its good conditions and the doctor's constant presence. I believe it is the state's responsibility to provide many of these centers and support them all.

11. Are you satisfied with the quality of your care at this public center?

• Information : YES

• Communication: YES

- Health professionnal support : YES
- Medical care: YES
- Financial accessibility : YES

Thank you very much, that's the end of the interview. I'm going to stop recording now.
