# Supplementary material for: A realist evaluation of the development, implementation and outcomes of the first public ART Centre in Morocco
Source: PLOS Glob Public Health. 2026 Apr 20;6(4):e0005318. doi: 10.1371/journal.pgph.0005318 (PMC13094999; doi:10.1371/journal.pgph.0005318)
Supplement: S2 Data — (ZIP) [file pgph.0005318.s013.zip › S2_Data_Transcriptions_in _English/C12.pdf]

Interview for Men and Women with Infertility

Participant Code NUMBER: ——————C12

2. How long have you wanted to have a baby? Did you go to a private clinic (office) before coming to this center?

Woman said:

Two years of therapy since our marriage. Before starting treatment, I had a spontaneous pregnancy in the first few weeks, I had an abortion, then I went to a private doctor. The first was a general practitioner for six months of treatment, but without results. The second was a gynecologist; the treatment lasted almost a year.

Man said:

A family friend told us about this center. No doctor referred us to this center.

3. When did you come to the center? How long was it between registering at the center and starting treatment?

Woman said:

We arrived in August, and the waiting period between registration and treatment was one month. We have now completed six months of treatment. It is a good center, and the medical staff always gives me hope for the treatment. But only the staff at this center are pleasant, friendly, and patient.

4. What was your financial and psychological experience during your treatment period?

Woman says:

The financial aspect is the first obstacle to treatment, especially since treatment has

always been unsuccessful. Until I came to this center, I felt reassured and comfortable, even though I had already refused treatment at public centers because of the lack of appropriate conditions. Until I changed my mind after visiting this center and seeing how they gave us enough time to explain and simplify things.

Psychological support is crucial for couples; I would say it's everything!

5. How did you experience these years of treatment psychologically? How did the community view you?

Woman says:

People always ask about the reasons for the delayed pregnancy, which negatively affects me, bothers me, and hurts me. My mother tells me she cries every day for me and prays to God to give me children. I feel stuck in the treatment. In fact, in our Moroccan society, the emphasis is always placed on women as if they were responsible for delayed pregnancies and infertility. Society puts constant pressure on you; at every

encounter, at every contact, the same question: Are you pregnant?! Why aren't you pregnant?! It depresses me!

Man says:

We decided not to tell the family. I always try to ignore people's curiosity; they always ask you an embarrassing question about the delayed pregnancy. In my opinion, everyone should focus on their own life and not interfere in other people's private lives. A friend who got married after me still asks me the same question! Men suffer too, but in silence!

Society doesn't consider the suffering of couples; on the contrary, they make our lives even more difficult and negatively impact our well-being! And they always steer you towards traditional treatments.

6. Have you ever used traditional methods to get pregnant? Will you repeat this experience?

Woman says:

Yes, I have used traditional methods before and I drank herbs to help with pregnancy, but without success. I stopped drinking them because I had stomach problems. I will never repeat this experience.

7. Are you satisfied with the quality of your care at this public center?

The doctor and the team spend over an hour explaining and re-explaining, and asking us if we understood. This has never been the case in private consultations.

- Information : YES
- Communication: YES
- Health professionnal support : YES
- Medical care: YES
- Financial accessibility : YES

8. Was the nurse consultation beneficial to you?

Woman said:

Yes, frankly, this center provides all the necessary help and telephone follow-up to facilitate and streamline all stages of treatment. At first, I was surprised to see the women being treated with such a friendly relationship with the center's staff. I am surprised by the quality of care in the public sector!

9. How much money have you already spent on diagnosis and treatment? Where did you get these funds? What helped you cope with the financial pressures?

Woman said:

We borrowed money because, although the treatment is less expensive in the public sector compared to the private sector, it is still costly and exceeds our financial means.

There are too many expenses, especially since the CNSS does not cover all the treatment costs.

10. In your opinion, do you think the IVF center has an effect? What is it?

Woman said:

Yes, the center has a positive effect on the patient's psychology, and the friendly and approachable staff, especially our attending physician, make me feel comfortable and ready for treatment and to follow all its stages.

11. Would you recommend the Center to your family and acquaintances? Why?

Woman said:

I will share information about this center with all my friends and family because it is a special, first-class center.

Thank you very much, this concludes the interview. I will stop recording now.
