# Supplementary material for: A realist evaluation of the development, implementation and outcomes of the first public ART Centre in Morocco
Source: PLOS Glob Public Health. 2026 Apr 20;6(4):e0005318. doi: 10.1371/journal.pgph.0005318 (PMC13094999; doi:10.1371/journal.pgph.0005318)
Supplement: S2 Data — (ZIP) [file pgph.0005318.s013.zip › S2_Data_Transcriptions_in _English/C13.pdf]

Interview for Men and Women with Infertility

Participant Code NUMBER: ——————C13

2. Experience with infertility before coming to this fertility clinic

Woman says:

At first, getting pregnant wasn't my problem; the problem was that I was having repeated miscarriages. After a year of marriage, I stopped taking the pill in an attempt to conceive. My pregnancy was normal without any treatment, and after each attempt, I had a miscarriage at the very beginning of the pregnancy. Then I went to see more than one doctor to diagnose my situation, and I couldn't understand the reasons for the miscarriages. In fact, I had seven consecutive miscarriages, and I can't describe to you how difficult and painful it was!

3. How did you experience these years of treatment psychologically? What is society's perception of infertility?

Woman said:

At first, I wasn't affected and I was optimistic because I was younger and fully supported by my environment. Everything changed after the ectopic pregnancy, then the ovary removal, and I had to undergo treatment to get pregnant, but it was still without success.

So then! I started to get frustrated, and I see the years going by without children, especially since the community's perspective is always focused on the woman, so she is primarily responsible for the delay in her pregnancy.

Man said:

I see these things as God's will, and I don't allow anyone to interfere with my affairs and my private life.

4. Did you go to a private clinic before coming to this center?

Woman said:

Yes, I went to see more than one doctor in the private sector. When I didn't see results, I went to see another, and when I heard about a doctor who was capable in situations like mine, I went to see him immediately.

5. How much money have you already spent on diagnosis and treatment?

Man said:

Currently, we borrowed money to get the tests done and pay for the treatment. The cost is very high, and I think these public centers should be present in most cities so that everyone can benefit from treatment because some people move from cities that are very far away; we also travel from Casablanca to Rabat. Basically, there's the financial aspect of the treatment, and then the travel costs add to it, making it more complicated.

Woman said:

The financial cost is high, especially because at the beginning of my treatment, my husband and I didn't have health insurance, so we paid for all the procedures. Now, we both have CNSS coverage, but most of the medications are not covered. Consultations cost between 500 and 600 DH, of which only 100 DH is covered, and the injections are expensive and not covered.

6. How did you come to this center? Who recommended it to you?

Man said:

I knew about this center through the internet, when I was randomly searching for more information on this topic.

Woman says:

My husband learned about this center online, and we went there with hope because in all honesty, it's less expensive than the private sector, especially since most doctors suggested that the last part of the treatment would be IVF, which is really expensive for me. During our first visit, we scheduled an appointment very close to the week before, considering the age factor.

7. What was your experience during your treatment at the center? Were your expectations met? How?

Man says:

We feel very comfortable here, and our doctor is very good and empathetic, which helps with the psychological aspect of the treatment. However, we still have the problem of tests and non-reimbursed medications.

Wife says:

The center is very good, much better than private practices. The cost is a bit high, but less than them. There are also transportation and travel costs; if a center like this exists in my city, we will save on mobility costs.

8. Have you shared your treatment at this center with your family and friends?

Woman said:

We don't share this information to protect our privacy and avoid embarrassing questions and explanations from people. Given the sensitivity of this subject, I haven't said anything, not even to my mother.

9. Was the nursing consultation beneficial to you? What is your opinion on the care you receive at the Center?

Yes, the nursing consultation was beneficial. I have only one comment about how I communicate with the center. Sometimes I need simple information, and because I can't contact them, I have to travel from Casablanca to Rabat to get the information, whereas I can only get it by phone. I would like to see an easier method of communication than a mobile phone to facilitate receiving treatment information for patients.

10. How do you get to the center? Do you always come together?

Woman said:

I often come by train; I take the 6:00 AM train so I can be on time.

11. Are you satisfied with the quality of your care at this public center?

• Information : YES

• Communication: YES

• Health professionnal support : YES

• Medical care: YES

• Financial accessibility : YES

12. Would you recommend the Center to your family and acquaintances? Why?

I have already recommended this center to women with the same condition because state centers are much better than the private sector and more trustworthy. The private sector is questionable, and there is no transparency in its treatment methods, which are illegal and not religious.

Thank you very much, this concludes the interview. I will stop recording now.
