# Supplementary material for: A realist evaluation of the development, implementation and outcomes of the first public ART Centre in Morocco
Source: PLOS Glob Public Health. 2026 Apr 20;6(4):e0005318. doi: 10.1371/journal.pgph.0005318 (PMC13094999; doi:10.1371/journal.pgph.0005318)
Supplement: S2 Data — (ZIP) [file pgph.0005318.s013.zip › S2_Data_Transcriptions_in _English/C14.pdf]

Interview for Men and Women with Infertility

Participant Code NUMBER: ——————-C14

1. What was your experience with infertility before coming to this fertility center? Did you go to a private clinic (office) before coming to this center?

Woman says:

I went to quite a few gynecologists as well as general practitioners. I saw more than four private gynecologists, changing doctors each time to get results. In fact, because of a misdiagnosis by one of the doctors, we had insemination done twice without first having an X-ray of the fallopian tubes. As a result, it didn't work. While I had a complete blockage of both fallopian tubes, which I discovered after another diagnosis from a different general practitioner with whom I continued treatment until we reached the IVF stage, we unfortunately didn't have it done due to our financial situation.

Man said:

Our experience with treatment in the private sector was long and expensive, especially since we don't have health insurance or medical coverage because I've been living in Spain for five years and my wife is in Morocco.

3. How much money have you already spent on diagnosis and treatment? Where did you get these funds?

Wife said:

The treatment is very expensive. There are tests and medications, and we don't have health insurance, so we pay for everything without any reimbursement.

In fact, we borrowed money for the treatment; insemination alone cost me 3,000 dirhams each time, and we couldn't risk a large sum for IVF for fear of failure and running out of money. Imagine the feelings of sadness and great disappointment; we're losing money, health, time, and the chance to have children because of a misdiagnosis.

4. How did you experience these years of treatment psychologically? How did the community view you?

Woman says:

My mental state was at its lowest. I'm very sensitive about this. Luckily, my husband's family supports me and they don't ask him to marry another woman. Because in Moroccan society, people get involved in these things, even if they are personal and sensitive. They always portray a woman who is late in getting pregnant as imperfect, incomplete without children, and she is blamed.

5. How did you decide to come to this center? Who recommended it to you?

Woman said:

My friend from Fez recommended this center to me. She had treatment here and had the operation, and it was successful. Now she's a mother.

6. How did you make the appointment?

Woman said:

My friend and I came to make an appointment because she was being treated here, and after a two-year wait, they called me to schedule it.

Man said:

When my wife came to the center before me, the doctor gave her all the necessary tests, which we had done. During our meeting with the doctor, she emphasized the time factor, and as you know, my stability in Spain has always been a time constraint between us.

7. Was the consultation with the nurse helpful? Did she explain all the financial details to you?

Woman:

Yes, the consultation was very helpful. Thanks to my girlfriend, I already had an idea of the costs, and I find it reasonable compared to a private clinic.

8. Did you take the time to convince your husband?

Wife says:

On the contrary, my husband has always supported me. It was I who suggested he marry someone else to have children, and he didn't agree.

9. Did you follow the same treatment as the clinic before?

No, IVF was a suggestion from my doctor, and we didn't have the money for it.

10. What is your opinion of the care you receive at the Centre?

The centre is excellent in every respect. They provide me with all the necessary conditions for treatment. The doctor explains to me in simple terms how to use the medication and the nurse repeats the explanation to me again. Whenever I need information, I call them and they answer by phone.

11. Are you satisfied with the quality of your care at this public centre?

• Information : YES

• Communication: YES

• Health professionnal support : YES

• Medical care: YES

• Financial accessibility : YES

12. Have you noticed any differences between the public IVF center and private centers?

Woman says:

The difference is enormous. There are many differences compared to my experience. The private sector is mainly interested in profit margins.

This center has great credibility; their primary concern is to help couples have children in various ways, with simplified information, sufficient time to explain and listen, and follow-up and support.

13. In your opinion, how can the State provide you with assistance?

Woman said:

The state needs to focus on this sector and support it; it's marginalized.

Man said:

The state should establish such centers in several cities so that everyone can benefit from treatment rather than spending money on transportation, especially since time is a crucial factor in the treatment process.

Thank you very much, that's the end of the interview. I'm going to stop recording now.
