# Supplementary material for: A realist evaluation of the development, implementation and outcomes of the first public ART Centre in Morocco
Source: PLOS Glob Public Health. 2026 Apr 20;6(4):e0005318. doi: 10.1371/journal.pgph.0005318 (PMC13094999; doi:10.1371/journal.pgph.0005318)
Supplement: S2 Data — (ZIP) [file pgph.0005318.s013.zip › S2_Data_Transcriptions_in _English/C1H.pdf]

Interview for Men and Women with Infertility

Participant Code NUMBER: __________C1H

2. Experience with infertility prior to coming to this ART Center

Now, I would like to ask you a few questions about your experience with infertility before you came to this center.

2.1. What is it like to have infertility in Morocco?*[Researcher: Probe Context]*

For me, it takes several steps to find the right one, and the process is very long; you get lost in the system, and the information available at the public center is insufficient, and many couples can't accept the problem.

2.2. How did you experience your infertility before your consultation in this center?

We can't understand why we have this infertility problem since I myself already had children from my first marriage and the same thing for my second wife.

2.3. At psychological level?*[researcher to probe stigma, mental health, anxiety, mood]*

It causes me a lot of pressure and anger

2.4. At economic level?*[researcher to probe effect on finances, household savings, loans]*

Yes, it's difficult and very expensive.

2.5. At the family level?*[researcher to probe effect on relations with spouse, in-laws]*

Yes, there is family pressure since we are married and many couples in the family got married after us and had children.

2.6. At the Social level?*[researcher to probe stigma, discrimination, exclusion, etc]*

For me, what the other person thinks isn't important. In the general mentality of society, children are the pillar of the family in our Moroccan context, and every marriage should result in children.

3. Help seeking and first impressions

3.1. How did you come into contact with this ART Center? *[researcher to probe: How did the participant obtain information about this Center? Did they consult any friends or relatives or professionals and asked for their recommendations?]*

It was through my wife's gynecologist who advised her to consult at this center.

3.2. What were your impressions and feelings the first time you learned about the possibility to visit this ART center?

My wife and I were very relieved when we learned that this center exists. Personally, I believe in the good quality of services provided by public hospitals because they are non-profit, serving the common social and humanitarian good; therefore, medical decisions are well-deliberated and carefully considered.

3.3. What were your expectations before starting your care at this center?

To achieve a good result and a successful operation, and to ensure a pleasant environment that allows the patient to maintain good health.

4. Experiences of accessing care at the ART Center

4.1. What was your experience during your treatment at the center? Were your expectations met? How so?

Yes, my expectations are very much met.

4.2. What is your opinion about the care that you are receiving at the Center?

It was a one-time test; the environment provides quality service and good conditions to meet the patient's needs.

4.3. Are you satisfied with the quality of your care at this public center:

• Information : YES

• Communication: YES

• Health professionnal support : YES

• Medical care: YES

• Financial accessibility : YES

4.4. Was the nursing consultation beneficial for you?

Yes

4.5. Why?

Given the criteria already mentioned.

4.6. Have you at any point in time considered stopping treatment from this center? Why?

Not included.

4.7. How much money have you already spent on diagnosis and treatment? Where did you obtain those funds from? What helped you to cope with the financial pressures?

At the moment I have no idea about the exact amount of care provided at the center, and to pay for this care I will rely on my own resources.

5. Benefits of a public ART Center

5.1. Had you attended a private clinic prior to coming to this ART center?

Yes

5.2. If so, were there any differences you noticed between the public ART Center and the private ART Centers? If yes, what were they?

In my case, for my wife who has tubal obstruction, they initially offered me a tubal plasty other than IVF, which is very expensive and doesn't have as many chances of success as IVF, which led us to decide to consult the center to do IVF.

5.3. In your opinion, do you think that the ART centre is having an effect? Which one?

Yes of course.

5.4. Would you recommend the Center to your family and acquaintances? why?

Yes, we recommend it; that's what we hope for in the future in terms of the end of our treatment.

5.5. What kind of people do you think would benefit most from a public ART Center and why?

I believe the low and middle classes.

5.6. In your view, which factors are contributing to the Center having an impact? How do these factors cause the Centre to have an effect? In what way? [Probe Mechanisms]

The first thing is to raise awareness in the media for all couples in Morocco and also to ensure free access and geographical accessibility.

5.7. What do you think are the reasons why people could be coming or failing to come to this ART Center?

Establish a free system and ensure that the state prioritizes couples suffering from infertility.

5.8. How can this center improve its services to other people in Morocco?

Guest houses should be planned near the center to improve geographical accessibility to the center, as well as hotels at symbolic cost, and the number of public IVF centers should be increased throughout the Kingdom.

5.9. Do you think that people in other countries should have a Centre such as this and why?

I have heard many testimonials from people living abroad regarding the good reputation of this first public IVF center in Morocco, which brings honor to our country for its healthcare services in this area of reproduction. I agree that all couples across all countries should have access to the services of such public IVF centers.

Thank you very much, that is the end of the interview. I will stop the recording now.
