# Supplementary material for: A realist evaluation of the development, implementation and outcomes of the first public ART Centre in Morocco
Source: PLOS Glob Public Health. 2026 Apr 20;6(4):e0005318. doi: 10.1371/journal.pgph.0005318 (PMC13094999; doi:10.1371/journal.pgph.0005318)
Supplement: S2 Data — (ZIP) [file pgph.0005318.s013.zip › S2_Data_Transcriptions_in _English/C1W.pdf]

Interview for Men and Women with Infertility

Participant Code NUMBER: __________C1W

2. Experience with infertility prior to coming to this ART Center

Now, I would like to ask you a few questions about your experience with infertility before you came to this center.

2.1. What is it like to have infertility in Morocco?*[Researcher: Probe Context]*

It's a very difficult problem on all levels, a long road to get the exact diagnosis, and lost in the process.

2.2. How did you experience your infertility before your consultation in this center?

2.3. At psychological level?*[researcher to probe stigma, mental health, anxiety, mood]*

I suffered a lot psychologically, of all kinds: stress, anger, etc.

2.4. At economic level?*[researcher to probe effect on finances, household savings, loans]*

Very expensive

2.5. At the family level?*[researcher to probe effect on relations with spouse, in-laws]*

I encountered many problems with my spouse and in-laws.

2.6. At the Social level?*[researcher to probe stigma, discrimination, exclusion, etc]*

There is a lot of stigma surrounding this problem in our society.

3. Help seeking and first impressions

3.1. How did you come into contact with this ART Center? *[researcher to probe: How did the participant obtain information about this Center? Did they consult any friends or relatives or professionals and asked for their recommendations?]*

Friends told me about this center and I made an appointment.

3.2. What were your impressions and feelings the first time you learned about the possibility to visit this ART center?

I was very happy, full of hope.

3.3. What were your expectations before starting your care at this center?

I expect good quality care and a good result.

4. Experiences of accessing care at the ART Center

4.1. What was your experience during your treatment at the center? Were your expectations met? How so?

Not included

4.2. What is your opinion about the care that you are receiving at the Center?

Very good, good driving and good listening skills, explanations provided

4.3. Are you satisfied with the quality of your care at this public center:

• Information : YES

• Communication: YES

• Health professionnal support : YES

• Medical care: YES

• Financial accessibility : YES

4.4. Was the nursing consultation beneficial for you?

Yes

4.5. Why?

Good communication, good support by phone, good driving, they treated me well and kindly.

4.6. Have you at any point in time considered stopping treatment from this center? Why?

Not included

4.7. How much money have you already spent on diagnosis and treatment? Where did you obtain those funds from? What helped you to cope with the financial pressures?

A lot of money. Financial support from the family

5. Benefits of a public ART Center

5.1. Had you attended a private clinic prior to coming to this ART center?

Yes

5.2. If so, were there any differences you noticed between the public ART Center and the private ART Centers? If yes, what were they?

A big difference, high competence, lower cost, good behavior compared to the private sector.

5.3. In your opinion, do you think that the ART centre is having an effect? Which one?

Yes

5.4. Would you recommend the Center to your family and acquaintances? why?

Yes, I've already done it for many friends because of its numerous advantages.

5.5. What kind of people do you think would benefit most from a public ART Center and why?

Yes, because it provides a good welcome, good behavior, and is less expensive.

5.6. In your view, which factors are contributing to the Center having an impact? How do these factors cause the Centre to have an effect? In what way? [Probe Mechanisms]

Yes, much less expensive, to solve the infertility problem.

5.7. What do you think are the reasons why people could be coming or failing to come to this ART Center?

For all the advantages already mentioned, good preparation, and many benefits

5.8. How can this center improve its services to other people in Morocco?

Many human resources, limiting the waiting list, I can't tell you more as I'm at the beginning of the process.

5.9. Do you think that people in other countries should have a Centre such as this and why?

Yes, it's to help people solve their infertility problems at a lower cost.

Thank you very much, that is the end of the interview. I will stop the recording now.
