# Supplementary material for: A realist evaluation of the development, implementation and outcomes of the first public ART Centre in Morocco
Source: PLOS Glob Public Health. 2026 Apr 20;6(4):e0005318. doi: 10.1371/journal.pgph.0005318 (PMC13094999; doi:10.1371/journal.pgph.0005318)
Supplement: S2 Data — (ZIP) [file pgph.0005318.s013.zip › S2_Data_Transcriptions_in _English/C2H.pdf]

Interview for Men and Women with Infertility

Participant Code NUMBER: ________C2H

2. Experience with infertility prior to coming to this ART Center

Now, I would like to ask you a few questions about your experience with infertility before you came to this center.

2.1. What is it like to have infertility in Morocco?*[Researcher: Probe Context]*

It's a very difficult problem; the hope of every married couple is to have children.

2.2. How did you experience your infertility before your consultation in this center?

2.3. At psychological level?*[researcher to probe stigma, mental health, anxiety, mood]*

Long process, several consultations.

2.4. At economic level?*[researcher to probe effect on finances, household savings, loans]*

Very expensive without medical coverage.

2.5. At the family level?*[researcher to probe effect on relations with spouse, in-laws]*

For my family, it represents their hope of having their grandchildren.

2.6. At the Social level?*[researcher to probe stigma, discrimination, exclusion, etc]*

It remains taboo for uneducated people and, for the majority, stigmatized.

3. Help seeking and first impressions

3.1. How did you come into contact with this ART Center? *[researcher to probe: How did the participant obtain information about this Center? Did they consult any friends or relatives or professionals and asked for their recommendations?]*

Through a referral from a doctor who had worked at that maternity ward.

3.2. What were your impressions and feelings the first time you learned about the possibility to visit this ART center?

Full of hope

3.3. What were your expectations before starting your care at this center?

Finding a good outcome and having children.

4. Experiences of accessing care at the ART Center

4.1. What was your experience during your treatment at the center? Were your expectations met? How so?

We received a good listening ear from the doctor, a good understanding, the explanation was clear, which gives us hope.

4.2. What is your opinion about the care that you are receiving at the Center?

It was very good.

4.3. Are you satisfied with the quality of your care at this public center:

• Information : YES

• Communication: YES

• Health professionnal support : YES

• Medical care: YES

• Financial accessibility : YES

4.4. Was the nursing consultation beneficial for you?

Yes

4.5. Why?

We received good support and good behavior, explanations were clear, and they treat people well.

4.6. Have you at any point in time considered stopping treatment from this center? Why?

Not included

4.7. How much money have you already spent on diagnosis and treatment? Where did you obtain those funds from? What helped you to cope with the financial pressures?

Savings, not personal funds.

5. Benefits of a public ART Center

5.1. Had you attended a private clinic prior to coming to this ART center?

Yes

5.2. If so, were there any differences you noticed between the public ART Center and the private ART Centers? If yes, what were they?

Yes, financially, it is cheaper than private.

5.3. In your opinion, do you think that the ART centre is having an effect? Which one?

It has a positive effect, especially on the cost.

5.4. Would you recommend the Center to your family and acquaintances? why?

Yes, for its advantages, they dedicate a lot of time to listening to patients

5.5. What kind of people do you think would benefit most from a public ART Center and why?

Every category, and every social class.

5.6. In your view, which factors are contributing to the Center having an impact? How do these factors cause the Centre to have an effect? In what way? [Probe Mechanisms]

Further cost reduction, good results, more human resources (doctors and nurses).

5.7. What do you think are the reasons why people could be coming or failing to come to this ART Center?

More human resources: doctors and nurses

5.8. How can this center improve its services to other people in Morocco?

For the reasons already mentioned, and also to provide for the reimbursement of medications, the medical coverage.

5.9. Do you think that people in other countries should have a Centre such as this and why?

Yes, because it's a global problem

Thank you very much, that is the end of the interview. I will stop the recording now.
