# Supplementary material for: A realist evaluation of the development, implementation and outcomes of the first public ART Centre in Morocco
Source: PLOS Glob Public Health. 2026 Apr 20;6(4):e0005318. doi: 10.1371/journal.pgph.0005318 (PMC13094999; doi:10.1371/journal.pgph.0005318)
Supplement: S2 Data — (ZIP) [file pgph.0005318.s013.zip › S2_Data_Transcriptions_in _English/C2W.pdf]

Interview Guide for Men and Women with Infertility

Participant Code NUMBER: ________C2W

2. Experience with infertility prior to coming to this ART Center

Now, I would like to ask you a few questions about your experience with infertility before you came to this center.

2.1. What is it like to have infertility in Morocco?*[Researcher: Probe Context]*

It is a difficult experience for most couples; for me, the process was very long and lost. I spent five years trying to get an accurate diagnosis of my infertility.

2.2. How did you experience your infertility before your consultation in this center?

This is a very difficult problem

2.3. At psychological level?*[researcher to probe stigma, mental health, anxiety, mood]*

Every time I think about it, I feel a lot of anxiety, stress, and anger.

2.4. At economic level?*[researcher to probe effect on finances, household savings, loans]*

Very expensive

2.5. At the family level?*[researcher to probe effect on relations with spouse, in-laws]*

Good relationship with my partner, but the in-laws always want to have their grandchildren.

2.6. At the Social level?*[researcher to probe stigma, discrimination, exclusion, etc]*

Discrimination, stigmatization, society always blames women, especially, the feeling of pitying infertile couples

3. Help seeking and first impressions

3.1. How did you come into contact with this ART Center? *[researcher to probe: How did the participant obtain information about this Center? Did they consult any friends or relatives or professionals and asked for their recommendations?]*

Through an acquaintance of mine who advised me to consult at this public center.

3.2. What were your impressions and feelings the first time you learned about the possibility to visit this ART center?

I was very happy and full of hope.

3.3. What were your expectations before starting your care at this center?

I expected a warm welcome, explanations, and a good result.

4. Experiences of accessing care at the ART Center

4.1. What was your experience during your treatment at the center? Were your expectations met? How so?

4.2. What is your opinion about the care that you are receiving at the Center?

The doctor's explanation was very clear and simplified; he listened well.

4.3. Are you satisfied with the quality of your care at this public center:

• Information : YES

• Communication: YES

• Health professionnal support : YES

• Medical care: YES

• Financial accessibility : YES

4.4. Was the nursing consultation beneficial for you?

Yes

4.5. Why?

Support, very good behavior.

4.6. Have you at any point in time considered stopping treatment from this center? Why?

Not included.

4.7. How much money have you already spent on diagnosis and treatment? Where did you obtain those funds from? What helped you to cope with the financial pressures?

I rely on my own resources and with financial help from the family.

5. Benefits of a public ART Center

5.1. Had you attended a private clinic prior to coming to this ART center?

Yes

5.2. If so, were there any differences you noticed between the public ART Center and the private ART Centers? If yes, what were they?

Yes, for example, the lower cost

5.3. In your opinion, do you think that the ART centre is having an effect? Which one? 5.4. Would you recommend the Center to your family and acquaintances? why?

Yes, and above all, the cost is lower.

5.5. What kind of people do you think would benefit most from a public ART Center and why?

People with limited resources

5.6. In your view, which factors are contributing to the Center having an impact? How do these factors cause the Centre to have an effect? In what way? [Probe Mechanisms]

Shorter waiting lists, lower costs, and a simpler medication process

5.7. What do you think are the reasons why people could be coming or failing to come to this ART Center?

Reduced waiting lists, good results and excellent treatment for couples, medical coverage, easy medication process with medications included in the cost

5.8. How can this center improve its services to other people in Morocco?

Reimbursement for medications, other centers in other cities, e.g., Tangier

5.9. Do you think that people in other countries should have a Centre such as this and why?

Yes, and it's to reduce the cost to suffering couples at a lower cost.

Thank you very much, that is the end of the interview. I will stop the recording now.
