# Supplementary material for: A realist evaluation of the development, implementation and outcomes of the first public ART Centre in Morocco
Source: PLOS Glob Public Health. 2026 Apr 20;6(4):e0005318. doi: 10.1371/journal.pgph.0005318 (PMC13094999; doi:10.1371/journal.pgph.0005318)
Supplement: S2 Data — (ZIP) [file pgph.0005318.s013.zip › S2_Data_Transcriptions_in _English/C3H.pdf]

Interview Guide for Men and Women with Infertility

Participant Code NUMBER: ____________C3H

2. Experience with infertility prior to coming to this ART Center

Now, I would like to ask you a few questions about your experience with infertility before you came to this center.

2.1. What is it like to have infertility in Morocco?*[Researcher: Probe Context]*

There is only one national center in Morocco, and it exists only in Rabat; this is insufficient.

2.2. How did you experience your infertility before your consultation in this center?

2.3. At psychological level?*[researcher to probe stigma, mental health, anxiety, mood]*

No problem, we have faith in God.

2.4. At economic level?*[researcher to probe effect on finances, household savings, loans]*

Of course, it remains expensive in the private sector.

2.5. At the family level?*[researcher to probe effect on relations with spouse, in-laws]*

This is perfectly normal in our relationship as a couple, as well as within the family.

2.6. At the Social level?*[researcher to probe stigma, discrimination, exclusion, etc]*

I don't see a problem

3. Help seeking and first impressions

3.1. How did you come into contact with this ART Center? *[researcher to probe: How did the participant obtain information about this Center? Did they consult any friends or relatives or professionals and asked for their recommendations?]*

Through a friend of my wife who advised her

3.2. What were your impressions and feelings the first time you learned about the possibility to visit this ART center?

Very happy, it went well

3.3. What were your expectations before starting your care at this center?

He gave us help, rescheduled the appointment, and arranged a suitable appointment; it was very good and yielded a positive result.

4. Experiences of accessing care at the ART Center

4.1. What was your experience during your treatment at the center? Were your expectations met? How so?

The doctor gave us enough time, ordered some tests, and it went well and was very satisfactory.

4.2. What is your opinion about the care that you are receiving at the Center?

A high-quality welcome and it went very well.

4.3. Are you satisfied with the quality of your care at this public center:

• Information : YES

• Communication: YES

• Health professionnal support : YES

• Medical care: YES

• Financial accessibility : YES

4.4. Was the nursing consultation beneficial for you?

Yes

4.5. Why?

A warm welcome, a good explanation, and satisfactory support.

4.6. Have you at any point in time considered stopping treatment from this center? Why?

Not included

4.7. How much money have you already spent on diagnosis and treatment? Where did you obtain those funds from? What helped you to cope with the financial pressures?

We have saved some money.

5. Benefits of a public ART Center

5.1. Had you attended a private clinic prior to coming to this ART center?

Yes

5.2. If so, were there any differences you noticed between the public ART Center and the private ART Centers? If yes, what were they?

Yes, of course, the cost is lower in the public center compared to the private one.

5.3. In your opinion, do you think that the ART centre is having an effect? Which one?

Yes, a low-cost option.

5.4. Would you recommend the Center to your family and acquaintances? why?

Yes, affordable price.

5.5. What kind of people do you think would benefit most from a public ART Center and why?

For everyone, the cost is affordable.

5.6. In your view, which factors are contributing to the Center having an impact? How do these factors cause the Centre to have an effect? In what way? [Probe Mechanisms]

Yes, no difficulties in the process, it was easy.

5.7. What do you think are the reasons why people could be coming or failing to come to this ART Center?

The cost is cheaper.

5.8. How can this center improve its services to other people in Morocco?

Increase the number of similar centers and improve geographical accessibility for other regions and several cities to limit travel for people residing in distant cities.

5.9. Do you think that people in other countries should have a Centre such as this and why?

Yes, it can be generalized in several countries, and the top officials in those countries are best placed to decide.

Thank you very much, that is the end of the interview. I will stop the recording now.
