# Supplementary material for: A realist evaluation of the development, implementation and outcomes of the first public ART Centre in Morocco
Source: PLOS Glob Public Health. 2026 Apr 20;6(4):e0005318. doi: 10.1371/journal.pgph.0005318 (PMC13094999; doi:10.1371/journal.pgph.0005318)
Supplement: S2 Data — (ZIP) [file pgph.0005318.s013.zip › S2_Data_Transcriptions_in _English/C3W.pdf]

Interview Guide for Men and Women with Infertility

Participant Code NUMBER: __________C3W

2. Experience with infertility prior to coming to this ART Center

Now, I would like to ask you a few questions about your experience with infertility before you came to this center.

2.1. What is it like to have infertility in Morocco?*[Researcher: Probe Context]*

It's very difficult; many couples suffer from it, more than half

2.2. How did you experience your infertility before your consultation in this center?

I suffered a lot, I consulted private doctors a lot, I spent all my money on private doctor consultations

2.3. At psychological level?*[researcher to probe stigma, mental health, anxiety, mood]*

It causes me a lot of stress; isolation, tension, bad mood

2.4. At economic level?*[researcher to probe effect on finances, household savings, loans]*

I have no resources, but I started working just to save up enough money to consult and undergo IVF.

2.5. At the family level?*[researcher to probe effect on relations with spouse, in-laws]*

I avoid visiting family, especially for happy occasions like weddings or baptisms. I prefer to stay home alone to avoid questions from family or acquaintances on the subject.

2.6. At the Social level?*[researcher to probe stigma, discrimination, exclusion, etc]*

There's a lot of stigma and discrimination; I stay completely alone to avoid questions from relatives on the subject.

3. Help seeking and first impressions

3.1. How did you come into contact with this ART Center? *[researcher to probe: How did the participant obtain information about this Center? Did they consult any friends or relatives or professionals and asked for their recommendations?]*

By chance, a woman I met recommended it to me

3.2. What were your impressions and feelings the first time you learned about the possibility to visit this ART center?

I was very happy, it gave me great hope

3.3. What were your expectations before starting your care at this center?

My expectations are to be successful and have a child.

4. Experiences of accessing care at the ART Center

4.1. What was your experience during your treatment at the center? Were your expectations met? How so?

A smile, good behavior, listening skills, and understanding, whether with a doctor or staff member.

4.2. What is your opinion about the care that you are receiving at the Center?

Quality care

4.3. Are you satisfied with the quality of your care at this public center:

• Information : YES

• Communication: YES

• Health professionnal support : YES

• Medical care: YES

• Financial accessibility : YES

4.4. Was the nursing consultation beneficial for you?

Yes

4.5. Why?

Quality reception, information, support, listening.

4.6. Have you at any point in time considered stopping treatment from this center? Why?

Not included.

4.7. How much money have you already spent on diagnosis and treatment? Where did you obtain those funds from? What helped you to cope with the financial pressures?

Family help, our own resources

5. Benefits of a public ART Center

5.1. Had you attended a private clinic prior to coming to this ART center?

Yes

5.2. If so, were there any differences you noticed between the public ART Center and the private ART Centers? If yes, what were they?

Less expensive, good service, welcoming atmosphere, information, quality care

5.3. In your opinion, do you think that the ART centre is having an effect? Which one?

Yes, of course it has a great effect, it's less expensive and the care is of high quality.

5.4. Would you recommend the Center to your family and acquaintances? why?

Yes of course

5.5. What kind of people do you think would benefit most from a public ART Center and why?

People who have no resources

5.6. In your view, which factors are contributing to the Center having an impact? How do these factors cause the Centre to have an effect? In what way? [Probe Mechanisms]

Good results, more Human Resources +++

5.7. What do you think are the reasons why people could be coming or failing to come to this ART Center?

5.8. How can this center improve its services to other people in Morocco?

Several centers in Morocco and many human resources in the center, the good results, also it must include laboratories.

5.9. Do you think that people in other countries should have a Centre such as this and why?

No response

Thank you very much, that is the end of the interview. I will stop the recording now.
