# Supplementary material for: A realist evaluation of the development, implementation and outcomes of the first public ART Centre in Morocco
Source: PLOS Glob Public Health. 2026 Apr 20;6(4):e0005318. doi: 10.1371/journal.pgph.0005318 (PMC13094999; doi:10.1371/journal.pgph.0005318)
Supplement: S2 Data — (ZIP) [file pgph.0005318.s013.zip › S2_Data_Transcriptions_in _English/C4 W.pdf]

Interview for Men and Women with Infertility

Participant Code NUMBER: ______C4W

2. Experience with infertility prior to coming to this ART Center

Now, I would like to ask you a few questions about your experience with infertility before you came to this center.

2.1. What is it like to have infertility in Morocco?*[Researcher: Probe Context]*

During my consultations in private practices, I observed that this problem of infertility among couples is very widespread in Morocco, especially in recent years; many people suffer greatly from it.

2.2. How did you experience your infertility before your consultation in this center?

I consulted many private clinics in search of the correct diagnosis, after several visits to various gynecologists. Finally, after a long journey, I understood the cause of my infertility, which is tubal obstruction.

2.3. At psychological level?*[researcher to probe stigma, mental health, anxiety, mood]*

It's very difficult, very stressful, especially since time passes quickly and with age the chances of pregnancy decrease. A lot of stress and anxiety.

2.4. At economic level?*[researcher to probe effect on finances, household savings, loans]*

It's very difficult, especially without health insurance. We only have RAMED, which doesn't cover infertility treatment. The cost of medication is very high, and so are the tests. For four years, I had many checkups, three to four per week.

2.5. At the family level?*[researcher to probe effect on relations with spouse, in-laws]*

Many problems arise with the spouse, partly due to their in-laws

2.6. At the Social level?*[researcher to probe stigma, discrimination, exclusion, etc]*

Many people constantly ask me questions like, "Why haven't you had children until now?" Some recommend I see this or that gynecologist, and I always feel like they pity me.

3. Help seeking and first impressions

3.1. How did you come into contact with this ART Center? *[researcher to probe: How did the participant obtain information about this Center? Did they consult any friends or relatives or professionals and asked for their recommendations?]*

Through a friend who gave birth at the maternity ward.

3.2. What were your impressions and feelings the first time you learned about the possibility to visit this ART center?

There's no comparison; the cost is significantly lower compared to the very high cost in the private sector, as a private doctor quoted me a very high price. I felt that a lot of hope was opening up.

3.3. What were your expectations before starting your care at this center?

That the operation will be successful and that there will be success and that I will become pregnant.

4. Experiences of accessing care at the ART Center

4.1. What was your experience during your treatment at the center? Were your expectations met? How so?

My experience is very good; the doctor dedicates sufficient time to us.

4.2. What is your opinion about the care that you are receiving at the Center?

Good

4.3. Are you satisfied with the quality of your care at this public center:

• Information : YES

• Communication: YES

• Health professionnal support : YES

• Medical care: YES

• Financial accessibility : YES

4.4. Was the nursing consultation beneficial for you?

Yes

4.5. Why?

They answer my questions, good conduct

4.6. Have you at any point in time considered stopping treatment from this center? Why?

(Not included)

4.7. How much money have you already spent on diagnosis and treatment? Where did you obtain those funds from? What helped you to cope with the financial pressures?

That's a reasonable amount, a little support from the family at least it's an affordable amount.

5. Benefits of a public ART Center

5.1. Had you attended a private clinic prior to coming to this ART center?

Yes

5.2. If so, were there any differences you noticed between the public ART Center and the private ART Centers? If yes, what were they?

It's about competence, the reasonable cost, and quality care.

5.3. In your opinion, do you think that the ART centre is having an effect? Which one?

Yes

5.4. Would you recommend the Center to your family and acquaintances? why?

Yes, because it's an opportunity that everyone like me should benefit from.

5.5. What kind of people do you think would benefit most from a public ART Center and why?

People who lack the resources, and those seeking to save time to shorten the process

5.6. In your view, which factors are contributing to the Center having an impact? How do these factors cause the Centre to have an effect? In what way? [Probe Mechanisms]

Reducing waiting times; medical coverage in the management of illness and reimbursement of medications.

5.7. What do you think are the reasons why people could be coming or failing to come to this ART Center?

The cost is affordable; the other reasons have been mentioned above.

5.8. How can this center improve its services to other people in Morocco?

Increase the number of HR staff at the center; still a lot of awareness-raising work to be done regarding the center's media coverage.

5.9. Do you think that people in other countries should have a Centre such as this and why?

No answer.

Thank you very much, that is the end of the interview. I will stop the recording now.
