# Supplementary material for: A realist evaluation of the development, implementation and outcomes of the first public ART Centre in Morocco
Source: PLOS Glob Public Health. 2026 Apr 20;6(4):e0005318. doi: 10.1371/journal.pgph.0005318 (PMC13094999; doi:10.1371/journal.pgph.0005318)
Supplement: S2 Data — (ZIP) [file pgph.0005318.s013.zip › S2_Data_Transcriptions_in _English/C4H.pdf]

Guide d'entretien pour les hommes et les femmes infertiles

NUMÉRO de code de participant : _____C4 H

2. Experience with infertility prior to coming to this ART Center

Now, I would like to ask you a few questions about your experience with infertility before you came to this center.

2.1. What is it like to have infertility in Morocco?*[Researcher: Probe Context]*

During my visits to gynecologists, I noticed that the number of infertile couples seeking consultation is very considerable; the problem is widespread in Morocco, as in other countries.

2.2. How did you experience your infertility before your consultation in this center?

My wife and I consulted many doctors, but in vain. We spent a lot of time consulting privately.

2.3. At psychological level?*[researcher to probe stigma, mental health, anxiety, mood]*

Of course, I felt a lot of suffering and stress due to the lack of good results.

2.4. At economic level?*[researcher to probe effect on finances, household savings, loans]*

We spent a lot of money.

2.5. At the family level?*[researcher to probe effect on relations with spouse, in-laws]*

I received some psychological support from my family.

2.6. At the Social level?*[researcher to probe stigma, discrimination, exclusion, etc]*

At every family event or gathering, the idea of having children comes up for us, and it upsets us a great deal.

3. Help seeking and first impressions

3.1. How did you come into contact with this ART Center? *[researcher to probe: How did the participant obtain information about this Center? Did they consult any friends or relatives or professionals and asked for their recommendations?]*

Through a friend of my wife who gave birth at the maternity ward, and it was she who showed her the fertility center.

3.2. What were your impressions and feelings the first time you learned about the possibility to visit this ART center?

We immediately thought that the cost of treatment would be much less expensive than private treatment; it was a great hope that opened up for us.

3.3. What were your expectations before starting your care at this center?
My ultimate hope is that I will have a good result and have a baby, whether it's a boy or a girl, God willing.

4. Experiences of accessing care at the ART Center

4.1. What was your experience during your treatment at the center? Were your expectations met? How so?
Yes, it went very well.

4.2. What is your opinion about the care that you are receiving at the Center?

A very warm welcome, on the day of the appointment, you are seen without too much waiting, everything goes smoothly.

4.3. Are you satisfied with the quality of your care at this public center:

• Information : YES

• Communication: YES

• Health professionnal support : YES

• Medical care: YES

• Financial accessibility : YES

4.4. Was the nursing consultation beneficial for you?

Yes

4.5. Why?

Very beneficial. The whole team does what is necessary to listen to us and answer our questions and clarify the process.

4.6. Did you at any point consider stopping treatment at this center? Why?

Not included for waiting list, no start of treatment.

4.7. How much money have you already spent on diagnosis and treatment? Where did you get these funds? What helped you cope with the financial pressures?

In total, it will cost us 22,000 dh, which is much less expensive than the private option, which costs 40,000 dh. We will undertake the treatment using our own resources and funds.

5. Benefits of a public ART Center

5.1. Had you attended a private clinic prior to coming to this ART center?

Yes

5.2. If so, were there any differences you noticed between the public ART Center and the private ART Centers? If yes, what were they?
Yes, there's a big difference. Even in the private sector, they asked for a fee of 40,000 dirhams, which is very expensive compared to the cost of treatment at this center.

Furthermore, medical consultations in the private sector don't exceed 10 minutes, whereas the doctor at this center takes more than half an hour to listen to us and explain the process, as does the nursing team.

5.3. In your opinion, do you think that the ART centre is having an effect? Which one?

Yes, of course, the cost is lower than in the private sector, and the staff is very helpful.

5.4. Would you recommend the Center to your family and acquaintances? why?

Of course, I recommend it for its advantages.

5.5. What kind of people do you think would benefit most from a public ART Center and why?

The middle class, especially, will benefit.

5.6. In your view, which factors are contributing to the Center having an impact? How do these factors cause the Centre to have an effect? In what way? [Probe Mechanisms]

[Probing mechanisms] The waiting time needs to be reduced for greater satisfaction, and the center needs to be publicized to raise awareness.

5.7. What do you think are the reasons why people could be coming or failing to come to this ART Center?

The reduced cost and the competence of its staff.

5.8. How can this center improve its services to other people in Morocco?
Recruit more human resources, establish and expand medical coverage in the field of assisted reproductive technology (ART), especially for couples with limited resources.

5.9. Do you think that people in other countries should have a Centre such as this and why?

[No response]

Thank you very much, that's the end of the interview. I'm going to stop recording now.
