# Supplementary material for: A realist evaluation of the development, implementation and outcomes of the first public ART Centre in Morocco
Source: PLOS Glob Public Health. 2026 Apr 20;6(4):e0005318. doi: 10.1371/journal.pgph.0005318 (PMC13094999; doi:10.1371/journal.pgph.0005318)
Supplement: S2 Data — (ZIP) [file pgph.0005318.s013.zip › S2_Data_Transcriptions_in _English/C5.pdf]

Interview for Men and Women with Infertility

Participant Code NUMBER: ___________C5

Today we are here to evaluate the first Moroccan center for ART to see how this center has operated, what its strengths and weaknesses are, and how we can improve upon them so that this center can become a national model. What is the significance of this center? What results has it achieved over the years? The best people to judge this center are those who have benefited from its services, in partnership with the management, of course, so that we can help others. As you know, it's a difficult experience. So, since you have agreed to share your experience of having a baby with us, this discussion will be anonymous. Madam, how long have you been trying to get pregnant and have a baby?

I got married and before that I worked far from my city in the mountains. So I only came back for holidays for about 10 years, during which I never got pregnant.

Later, when I wanted to have a baby, I started consulting with specialist doctors and also tried natural remedies. I had all the tests done; our scans were always normal. I always go when someone recommends a gynecologist or when I hear about a recommended doctor, and always, towards the end of the consultation, I ask the doctor why they didn't give me any treatment to follow, and I always get the same answer: "Why would I prescribe you treatment when you have nothing wrong? Everything is normal; your examination is unremarkable." "You can have a baby."

"Once I tried a natural remedy, and my husband and I lost consciousness from 6 PM until 6 AM. From that moment on, I decided to stop and follow the doctors' advice."

Did you buy the natural remedy or make it yourself?

"No, I bought it."

"Who recommended you use natural remedies?"

"Either my mother's friends or I searched on the internet. And honestly, we weren't in a rush to have a baby. We were understanding and reconciled."

"Didn't you experience any pressure during those years, whether from your husband's family, your family, friends, or society in general? And how do you feel when you receive questions about it, and how do you think society sees you as a couple who have been together for a long time but haven't had a baby?"

Not at all. My husband's family and he himself have been very understanding, but

sometimes I get questions like, "You haven't gotten pregnant yet, aren't you thinking about having a baby?" and I feel very bad because they think I can, but it's me who doesn't want a baby, and I've become very sensitive about this subject, and I will always try to change the subject. But society still considers the woman to be at fault in the

relationship, even if the problem comes from the man. And I know people who have had a psychological impact on them.

From a financial or material point of view, did the frequent or repeated medical consultations and treatments affect you?

No, I had supplemental health insurance and was reimbursed for all consultations; I never had any treatment. And they told me that just because you're not yet stable in your relationship with your husband, even though I was with him for three years and I didn't get pregnant. One day I heard the name of a highly recommended doctor, so I went to see him and brought my medical file with me. I felt very comfortable with him and told him my whole story, and he referred me to another doctor for assisted reproductive technology (ART). One day, a friend told me about a fertility center that had recently opened, and since my friends had always encouraged me to have children, they recommended it. Because I had a lot of work, I gave up. One day she asked me if I was leaving and I said no, so she scolded me. At that point, I decided to leave, and that's when I discovered there was an assisted reproductive technology (ART) center. There, I felt very comfortable with the doctors, nurses, and the entire team thanks to their kindness.

How were you treated?

I took my complete file with me, and when I met up with the entire female team, I felt more at ease. I explained my case again, and she gave my husband and me some tests to do. After that, we started the IVF procedures.

What are your opinions and observations regarding your experience with this state-run center? And what about your previous private consultations?

I was very satisfied and comfortable from the first time I went there with the team and especially with the doctor who treated me like a sister in her own way, the way she spoke to me and explained things to me. Their approach was excellent, so I decided to stay with them until the end.

How many attempts did you have?

The doctor explained that there are two ways: simulation and IVF. But since she told me that the results of IVF are more guaranteed, I preferred to do it to avoid wasting my money on simulation. We had the requested tests done and we got to work.

So, how was this experience for you, and was it successful or not, and when?

At first, I didn't tell anyone I was going to do IVF because I wanted to see how it would go first, and it wasn't quick. She gave me hormones to take.

Did you buy them, and were you reimbursed for them?

No.

Did this affect you financially?

Since I was eager to have a baby, if I didn't have money, I borrowed it from my friends. So I started taking hormones and learned how to inject them myself.

Who taught you the injection technique?

The doctor and the nurses, and they also taught my husband because I was afraid to inject myself. At first, we went to the doctor's office, then we started doing it at home.

And during this time, did your husband always accompany you?

I would go alone, and when they needed him, we would go together. And when I take the hormones for a period of time afterward, we do the tests early in the morning.

And your job didn't cause you any problems?

My principal was understanding and knew that I would be doing tests without knowing what they were for, and he supervises the students until I get to class. One day I received a call from the center and they told me I was ready for the egg retrieval. My husband and I filled out the paperwork. The procedure was very difficult. After a while, they injected my husband's sperm into the egg. After 15 days, I started to feel pain, so I went to them, and they reassured me every time. They were like family to me because I was obsessed. A few days later, the doctor gave me an ultrasound, and we heard the fetal heartbeat. We were so happy, me and the whole team, and I was the first successful case.

Was this your first IVF experience?

Yes, it was my first.

And she was the one who followed the pregnancy with you?

No, I continued my prenatal care at the Orangers Hospital.

Was this your first pregnancy, and did you give birth there at the maternity ward?

Yes, exactly at the maternity ward, and after the birth, the entire team from the center came to see me at the hospital—the nurses, the doctor…

So, was the communication with them pleasant?

Yes, it was extraordinary. The doctor was compassionate and modest.

What is the sex of your child?

It was a boy, and right after my period, I got pregnant naturally—a girl.

So, what is your opinion about these assisted reproductive technology centers and the private sector?

A friend of mine had three attempts in the private sector, and she complains about the poor, inhumane treatment. Each attempt cost 30,000 DH with no results. I only had one attempt, and it cost me 14,000 DH, with very good treatment. Because women in this situation are very sensitive and fragile, they need moral and psychological support.

In your opinion, what factors contribute to the success of state-run centers?

First, punctuality; second, the welcome and the way they speak and behave with patients, as if they were receiving a family member; and finally, the good price, but it would be desirable if there were reimbursement from health insurance companies, because there are people who cannot afford it, because these things are not secondary; there are people who are eager to have a baby.

And what negative points did you experience at this center that could be improved over time?

For me, I didn't notice anything negative; everything was excellent from the moment I entered the center until the end. They were professional, punctual, and very disciplined, and I hope that such centers will be established in all regions of Morocco.
