# Supplementary material for: A realist evaluation of the development, implementation and outcomes of the first public ART Centre in Morocco
Source: PLOS Glob Public Health. 2026 Apr 20;6(4):e0005318. doi: 10.1371/journal.pgph.0005318 (PMC13094999; doi:10.1371/journal.pgph.0005318)
Supplement: S2 Data — (ZIP) [file pgph.0005318.s013.zip › S2_Data_Transcriptions_in _English/C6.pdf]

Interview Guide for Men and Women with Infertility

Participant Code NUMBER: _________ C6

2. Experience with infertility prior to coming to this ART Center

I don't usually pay attention to society. But honestly, the experience of an infertile woman is hard; women are called "infertile," "unable to have children"... It affects you deeply, but I tried to resist people's comments. But it's too difficult, always anxious, sad, and burdened by my condition. The disappointment after several treatments without results. And financially, it's just too hard!

3. Help seeking and first impressions and 4. Experiences of accessing care at the ART Center

4.1. What was your experience during your treatment at the center? Were your expectations met? How so?

I was being treated privately by a gynecologist in Casablanca. I had PCOS and had two surgeries for ovarian cyst removal. After that, I had a laparoscopy. Then, I underwent treatment for tuberculosis. After polyp removal and several other procedures and treatments, I had follow-up care with a gynecologist in Rabat. After that, I came to the fertility center after the gynecologist who referred me there for IVF.

4.2. What is your opinion about the care that you are receiving at the Center?

I was surprised by the quality of service at the center; the OBGyn is lovely, as is the entire center team. Compared to the private sector, my experience was very positive. When I call, they answer and provide me with the information I need. And financially, it's incomparable. I underwent IVF, and it was successful; I had my son, thank God.

The only problem is that it's the only center in Morocco; there should be at least one per region. There are many women who suffer from this problem and need these services. The cost of IVF in the private sector is impossible for us and other women I've spoken with.

4.3. Are you satisfied with the quality of your care at this public center:

• Information : YES

• Communication: YES

• Health professionnal support : YES

• Medical care: YES

• Financial accessibility : YES

4.4. Was the nursing consultation beneficial for you?

Yes

4.5. Why?

The enhanced explanation, with a more emphatic style, answering the phone, following up, it's really very important and appreciated.

4.6. Have you at any point in time considered stopping treatment from this center? Why?

Not at all, and thank God it worked on the first try, and I plan to repeat it, God willing.

4.7. How much money have you already spent on diagnosis and treatment? Where did you obtain those funds from? What helped you to cope with the financial pressures?

I can't even begin to count the money we've spent over the past six years. Between the surgeries, treatments, and physical and psychological suffering, it's incredibly expensive and difficult to manage. But thankfully, my husband supported me; he paid for everything.

5. Benefits of a public ART Center

5.1. Had you attended a private clinic prior to coming to this ART center?

Yes

5.2. If so, were there any differences you noticed between the public ART Center and the private ART Centers? If yes, what were they?

There's a big difference in the care provided on all levels; First, the relationship: the OBGyn and team are very empathetic and communicate very well. The care is competent and seamless. Now I have my little one.

5.3. In your opinion, do you think that the ART centre is having an effect? Which one?

Thanks to this center I was able to have IVF and a child; otherwise, I would never have been able to have IVF in the private sector.

5.4. Would you recommend the Center to your family and acquaintances? why?

Absolutely, I recommend it to any woman suffering from this problem who doesn't have the financial means.

5.5. What kind of people do you think would benefit most from a public ART Center and why?

This infertility problem has become more common, and anyone hoping to benefit from quality infertility services and treatment at a lower cost would come to this center without hesitation. However, for those with limited means, it remains very expensive, and the distance also poses a problem.

5.8. How can this center improve its services to other people in Morocco?

The major problem lies in the cost of these services, the lack of information, and appropriate guidance. Few public facilities exist in Morocco. Therefore, we need these

centers throughout the kingdom, and for them to be covered by medical insurance. We also need to communicate about this subject, which remains taboo in society.

5.9. Do you think that people in other countries should have a Centre such as this and why?

Yes, absolutely, I saw people of foreign nationality and Moroccans living abroad at the center. They have more confidence in the Moroccan public service. But I think they also have problems accessing infertility treatment centers; it's absolutely essential to have them everywhere in the world.

Thank you very much, that is the end of the interview. I will stop the recording now.
