# Supplementary material for: A realist evaluation of the development, implementation and outcomes of the first public ART Centre in Morocco
Source: PLOS Glob Public Health. 2026 Apr 20;6(4):e0005318. doi: 10.1371/journal.pgph.0005318 (PMC13094999; doi:10.1371/journal.pgph.0005318)
Supplement: S2 Data — (ZIP) [file pgph.0005318.s013.zip › S2_Data_Transcriptions_in _English/C7.pdf]

Interview Guide for Men and Women with Infertility

Participant Code NUMBER: ________________

2. Experience with infertility prior to coming to this ART Center

The experience of an infertile woman is difficult; she is called "3agra," meaning "incapable of bearing children." Society places the blame squarely on the woman. It's like barren land; without children, the marriage cannot continue. People pay no attention to a woman's feelings, and at every opportunity, they hurt you, whether intentionally or not. I have experienced depression, difficult times, and suffering that I cannot describe in words. Financially, it's incredibly hard, especially for those of us in remote areas.

3. Help seeking and first impressions and 4. Experiences of accessing care at the ART Center

4.1. What was your experience during your treatment at the center? Were your expectations met? How so?

I was being treated privately by a gynecologist in Casablanca. I had PCOS and also problems with my fallopian tubes. Afterwards, I underwent several treatments and several cycles of ovulation induction without success. Then I came to the fertility clinic after a friend referred me to try IVF.

4.2. What is your opinion about the care that you are receiving at the Center?

I was surprised by the quality of the center's reception; the OBGyn is lovely, very empathetic and attentive, as is the entire center team. Compared to the private sector, my experience was very positive. I had easy access and received excellent care. I underwent IVF, and it was successful; I had my little girl, thank God.

The only problem was the distance; my husband and I had to go to great lengths to get there. We didn't have a place to live, nor the means to afford one. This is the only center of its kind in Morocco; there should be at least one per region. Many women suffer from this problem and need these services.

But even so, with the reduced price, we were fortunate enough to have IVF. The cost of IVF in the private sector is impossible for us!

4.3. Are you satisfied with the quality of your care at this public center:

• Information : YES

• Communication: YES

• Health professionnal support : YES

• Medical care: YES

• Financial accessibility : YES

4.4. Was the nursing consultation beneficial for you?

Yes

4.5. Why?

The enhanced explanation, with a more emphatic style, answering the phone, following up, it's really very important and appreciated.

4.6. Have you at any point in time considered stopping treatment from this center? Why?

Not at all, and thank God it worked out, and I was ready to persevere until the end. And I plan to do it again, God willing.

4.7. How much money have you already spent on diagnosis and treatment? Where did you obtain those funds from? What helped you to cope with the financial pressures?

Wow, a fortune! Between the travel, consultations, treatments, and physical and psychological suffering. But what I can say is that it's very expensive and difficult to manage. Thanks to loans and financial support from family and friends, my husband and I were able to pay all these expenses.

5. Benefits of a public ART Center

5.1. Had you attended a private clinic prior to coming to this ART center?

Yes

5.2. If so, were there any differences you noticed between the public ART Center and the private ART Centers? If yes, what were they?

There's a huge difference in the care provided on every level; it's like comparing apples and oranges! First, the relationships are excellent: the OBGyn and team are very empathetic and communicate very well. The competence and fluidity of the care provided are also remarkable. The organization of the facility is exemplary.

5.3. In your opinion, do you think that the ART centre is having an effect? Which one?

This center provides an invaluable service to an underserved population. Thanks to this center, I was able to undergo IVF and have a child; otherwise, I would never have been able to access IVF in the private sector.

5.4. Would you recommend the Center to your family and acquaintances? why?

Absolutely, I recommend it to all women who suffer from this problem and do not have the financial means.

5.5. What kind of people do you think would benefit most from a public ART Center and why?

This infertility problem has become more common, and anyone hoping to benefit from quality services and affordable infertility treatment would readily come to this center.

However, for those with limited means, it remains very expensive, and the distance also poses a problem.

5.8. How can this center improve its services to other people in Morocco?

The major problem lies in the cost of these services, the lack of information, and appropriate guidance. Few public facilities exist in Morocco. Therefore, we need these centers throughout the kingdom, and for them to be covered by medical insurance. We also need to communicate about this subject, which remains taboo in society.

5.9. Do you think that people in other countries should have a Centre such as this and why?

Yes, absolutely, all over the world, couples suffering from this problem should have public infertility treatment centers like any other for other illnesses.

Thank you very much, that is the end of the interview. I will stop the reco
