# Supplementary material for: A realist evaluation of the development, implementation and outcomes of the first public ART Centre in Morocco
Source: PLOS Glob Public Health. 2026 Apr 20;6(4):e0005318. doi: 10.1371/journal.pgph.0005318 (PMC13094999; doi:10.1371/journal.pgph.0005318)
Supplement: S2 Data — (ZIP) [file pgph.0005318.s013.zip › S2_Data_Transcriptions_in _English/C8.pdf]

Interview Guide for Men and Women with Infertility

Participant Code NUMBER: __________C8

2. Experience with infertility prior to coming to this ART Center

Now, I would like to ask you a few questions about your experience with infertility before you came to this center.

Infertility is a major problem in Morocco; it would take me days and hours to explain and describe our suffering. I tried everything, even going to a herbalist out of desperation, which was very expensive. I tried cupping (Hijama) and also witchcraft ("tat9af" and "chaaouada"). And that's not even mentioning the number of general practitioners and 13 specialists and renowned doctors that my husband and I consulted. A long and difficult journey of suffering can't be summarized in a few minutes.

Hormonal treatment affects a woman's psychological state, in addition to the lack of results. This forces me to change doctors every time.

We have marital problems and are close to divorce, and we have agreed to plan for divorce in case of IVF failure.

3. Help seeking and first impressions

I saw a television program about infertility in Morocco and people selling their belongings to pay for services. I heard half the price, and that encouraged us. We went to the center, but I was shocked to find a waiting list with couples from all over Morocco. I waited three years. The pace of work was so slow. I completely forgot about it and changed my approach, focusing on spiritual Ruqyah. I was completely desperate and depressed. At that moment, I received the call of my life. The center called me and my husband to come.

4. Experiences of accessing care at the ART Center

4.1. What was your experience during your treatment at the center? Were your expectations met? How so?

The behavior of the healthcare team is a contributing factor and provides support to these couples who are experiencing psychological problems. But during my treatment, we faced a medication shortage and also a genital infection after taking the treatment, and I had to pay again. My pregnancy coincided with COVID-19 and the lockdown, and I had my follow-up care with a gynecologist in Casablanca. I experienced the entire pregnancy at home, and I tried to connect with another woman in the same situation to support each other.

4.3. Are you satisfied with the quality of your care at this public center:

• Information : YES

• Communication: YES

• Health professionnal support : YES

• Medical care: YES

• Financial accessibility : YES

4.4. Was the nursing consultation beneficial for you?

Yes

4.5. Why?

The treatment isn't easy, and here I learned how to give myself the injection and understand the treatment steps at my own pace. And there's close follow-up afterwards by the center staff.

4.6. Have you at any point in time considered stopping treatment from this center? Why?

No, never, it was a hope of achieving a lifelong dream.

4.7. How much money have you already spent on diagnosis and treatment? Where did you obtain those funds from? What helped you to cope with the financial pressures?

We spent a fortune, a lot of money, sometimes loans, savings.

5. Benefits of a public ART Center

Excellent care, especially the plans, except for the transportation problems in my city. I need to repeat IVF, but I still have to wait; the demand is high. We need to establish several centers; we need them in Casablanca. People come from very far away. We need centers in every city. These couples are suffering, and we shouldn't add to their suffering.

Thank you very much, that is the end of the interview. I will stop the recording now.
