# Supplementary material for: A realist evaluation of the development, implementation and outcomes of the first public ART Centre in Morocco
Source: PLOS Glob Public Health. 2026 Apr 20;6(4):e0005318. doi: 10.1371/journal.pgph.0005318 (PMC13094999; doi:10.1371/journal.pgph.0005318)
Supplement: S2 Data — (ZIP) [file pgph.0005318.s013.zip › S2_Data_Transcriptions_in _English/P1.pdf]

Interview Guide for Healthcare Providers

Participant Code Number: —————P1

I would now like to start recording our conversation.

1. What is your profile?

Physician

2. How many years of experience in this role do you have?

<5 years
5 to 10 years: X
10 years

3. Please tell me about various fertility care services (e.g., preventive, diagnostic, and treatment) available at your clinic/department *[researcher to facilitate the conversation according to the services.]*

We do not have a structured plan. As a state physician, I conduct infertility consultations at the hospital for couples who have no established management plan. They may come for consultation but can then be lost to follow-up; there is no clear pathway. There should be a clear pathway, meaning a strategic management plan. So, only diagnostic services are available.

4. Why are these services important in our setting?

They are important because infertility is something increasingly common, something we encounter frequently, and it is a problem in society that needs to be treated.

5. What is your role in the management of infertile couples within the ART unit?

My role is to refer patients to the ART center.

6. Is there a basic training in the management of infertile couples and ART in Morocco?

No

7. Have you received a training in the management of infertile couples and ART?

No

9. What was the situation like before the ART Center was put in place? How were couples

accessing services? What problem did the ART Center solve?

Private centers handled infertility. The financial burden was the main problem that the public center helped solve.

10. What were your contributions in the implementation of the first public ART Center?

As a physician assigned to a public hospital, my role is simply to refer patients to the center.

11. During the implementation of the ART Center, did you face any challenges?

No participation → question not applicable.

13. What were the achievements of the implementation of the ART Center?

(Not answered)

14. Since the creation of the ART Center, did you contribute in any way in the improvement of the management of infertile couples in Morocco? Yes

If yes, how?
By referring couples to the public center.

16. Do you think that the Centre is having an effect? Which one?

Yes, it has an effect. It provides a solution to infertility. Mainly accessibility—especially in terms of cost—since it is much cheaper.

17. Which people do you think is being affected most (positively or negatively) by the Centre? Why is that? *[Probe Context and Mechanisms]*

Mainly the population with limited financial means. This is my observation, and that is why the waiting list is very long.

18. In your view, which factors are contributing to the Center having an impact? How do these factors cause the Centre to have an effect? In what way? *[Probe Mechanisms]*

The success rate of the center, the results, the availability of qualified human resources through training, cost, accessibility, and medical coverage to facilitate access.

19. Compared to the need, what is your view on the availability of Government hospitals and public ART Centers that can treat couples with infertility? *[To probe further, researcher will point out that most of these services are in large cities and need more services.]*

We only have one public ART center. No, we need several centers, more human resources, a specialized module or subspecialty, and openings for training positions.

20. What else needs to be done to increase couples' access to preventive, diagnostic and therapeutic interventions for infertility?

Resources, qualified human resources through training, cost reduction, accessibility, and medical coverage to facilitate access.

21. What are the three most important lessons you learned from your experience in theimplementation and thereafter the clinical management of the ART Center?

(Not included)

22. According to your experience, what would be your recommendations to other low- and middle-income countries if they want to implement public ART Centers?

Considering the population size, people must have access. There should be cooperation and support from organizations. For sustainability, three essential things are required: more human resources, more means, and more training.

Thank you very much; this is the end of the interview. I will stop the recording now.
