# Supplementary material for: A realist evaluation of the development, implementation and outcomes of the first public ART Centre in Morocco
Source: PLOS Glob Public Health. 2026 Apr 20;6(4):e0005318. doi: 10.1371/journal.pgph.0005318 (PMC13094999; doi:10.1371/journal.pgph.0005318)
Supplement: S2 Data — (ZIP) [file pgph.0005318.s013.zip › S2_Data_Transcriptions_in _English/P2.pdf]

Interview Guide for Healthcare Providers

Participant Code Number: ——————P2

I would now like to start recording our conversation.

1. What is your profile?
Physician

2. How many years of experience in this role do you have?

5 to 10 years: X

3. Please tell me about various fertility care services (e.g., preventive, diagnostic, and treatment) available at your clinic/department *[researcher to facilitate the conversation according to the services.]*

As a state physician, since the opening of the public ART center at the Orangers Maternity Hospital, we no longer face this problem. Thanks to this state-run center, physicians outside Rabat can refer patients there. The patients are well received, and the center does more than necessary to address their infertility problems.

4. Why are these services important in our setting?

They are important because these are couples who come seeking help; they are in demand and they suffer. Now that the center is available and we can relieve them, they are in good hands.

5. What is your role in the management of infertile couples within the ART unit?

My role is to refer couples to the center.

6. Is there a basic training in the management of infertile couples and ART in Morocco?

Now, yes.

7. Have you received a training in the management of infertile couples and ART?

Yes

8. If yes, could you tell me where, for how long, what were the objectives of the training?
(Details not provided)

9. What was the situation like before the ART Center was put in place? How were couples accessing services? What problem did the ART Center solve?

Private centers are very expensive, so patients suffered a lot. We could only tell them to wait, hoping that one day a public center would take their financial situation into account. After the creation of the center—thank God—we overcame this problem. The center makes our work easier because it specializes in infertility; we send patients directly for treatment. And regarding cost, it is much cheaper.

10. What were your contributions in the implementation of the first public ART Center?

Referring and sending patients to the center. I rely on the center.

11. During the implementation of the ART Center, did you face any challenges?

No participation → not applicable.

13. What were the achievements of the implementation of the ART Center?

Reducing the suffering of Moroccan couples, training gynecologists specialized in infertility, training paramedical staff, and acquiring equipment, consumables, and the latest technologies.

14. Since the creation of the ART Center, did you contribute in any way in the improvement of the management of infertile couples in Morocco?

Yes

15. If yes, How?
By referring couples to the public center.

16. Do you think that the Centre is having an effect? Which one?

The center has brought many benefits to infertile couples, whether with primary or secondary infertility. It plays a very important role—not only in receiving patients. Moroccan couples know there is a first public ART center in Morocco, and they feel relieved because they are well received and they can finally resolve their problem. It is accessible and less expensive; it provides financial accessibility because they have limited means.

17. Which people do you think is being affected most (positively or negatively) by the Centre? Why is that? *[Probe Context and Mechanisms]*

All people benefit equally; there is no difference between ethnicities. Any married couple, whether foreign or Moroccan, of any nationality, can access the services and have the same rights as Moroccan couples.

18. In your view, which factors are contributing to the Center having an impact? How do these factors cause the Centre to have an effect? In what way? *[Probe Mechanisms]*

Among the positive factors are the quality of reception—very important because these couples are suffering, and when they are received kindly, they feel satisfied and relieved, and we listen to them. And of course, the low cost.

19. Compared to the need, what is your view on the availability of Government hospitals and public ART Centers that can treat couples with infertility? *[To probe further, researcher will point out that most of these services are in large cities and need more services.]*

The center does not fully meet the needs of couples in Morocco. One center is not enough; it is impossible for a single center to handle all infertile couples. The number of centers is insufficient.

20. What else needs to be done to increase couples' access to preventive, diagnostic and therapeutic interventions for infertility?

Cost remains an issue, even in this first state center, although it is much cheaper. Also, the training of future gynecologists specialized in ART, ensuring sufficient qualified medical personnel, ensuring adequate stock of drugs and consumables, and having enough staff to handle all couples seeking services.

22. According to your experience, what would be your recommendations to other low- and middle-income countries if they want to implement public ART Centers?

Low- and middle-income countries can rely on funding sources that help people in need, and on cooperation between countries.

Thank you very much; this is the end of the interview. I will stop the recording now.
