# Supplementary material for: A realist evaluation of the development, implementation and outcomes of the first public ART Centre in Morocco
Source: PLOS Glob Public Health. 2026 Apr 20;6(4):e0005318. doi: 10.1371/journal.pgph.0005318 (PMC13094999; doi:10.1371/journal.pgph.0005318)
Supplement: S2 Data — (ZIP) [file pgph.0005318.s013.zip › S2_Data_Transcriptions_in _English/P3.pdf]

Interview for HealthCare Providers

Participant Code NUMBER: _________P3

I would now like to start recording our conversation.

Yes, I agree to the recording of this interview.

Have you received any training on assisted reproductive technology (ART) and the center's activities? Please tell me about the different fertility care services (e.g., preventative, diagnostic, and therapeutic) available at your center [researcher to facilitate the conversation, depending on the services].

Actually, during my residency, I did training very sporadically, meaning it was for a few days at a time, sometimes weekly, or a few times a month. I was passionate about the field of assisted reproductive technology. I would go to Dr. E.'s for internships on weekends and holidays. Then I learned that the IVF center was being set up so I tried to get a position there.

After my interview with Mr. B and Mr. E, they agreed to take me on the team, and that's when I went to Belgium for a year, a full-time year practically every day, holidays, weekends…I went to work because, since I was there alone, I had left my little family here, so I naturally thought I would learn as much as possible, so it was a very, very interesting year. In addition, from time to time, I went to have my interviews with Mr. E once a month, and I always scheduled the first appointment for 7:00 a.m., which left me the whole day to work at the Erasmus lab, so I could observe another team; how she works and subsequently, as in Liège, he didn't do freezing by vitrification. He only did slow freezing. We had discussed with Mr. E that I would go to Erasmus Hospital for a month to learn vitrification before coming here, and that's what I did. So during that year, I enrolled in the university diploma program in medically assisted reproduction; it was necessary to take this training so that I could have all the theoretical knowledge.

Did you pay for your diploma yourself?

I paid for the university diploma myself, even the travel expenses. In fact, everything, but it didn't bother me because I had to complete the training anyway. Practical training is never enough.

Are you the only one doing this at the center, or do you have a biologist?

No, I don't have a biologist because the one who was here left.

Did she take the training with you?

Yes, she even left before me. There was the biologist, the gynecologist, and another gynecologist. I think it was two years before, and I was the last person to leave. Actually, I left at the same time, a little before, but during the same period, I met the two lab technicians and the head nurse who is with us. So we left towards the end, and when we came back, the center was being set up, just a little bit after. Although, even when I was there, for example, I was working. We had worked on the plans with the

architect, and then, while I was there, I tried to work on the workbenches, the workbench heights, the outlets for the electrician, or where he should put the outlets... Now, I have no idea, actually, about the standards, if they exist in Morocco regarding that. We work on the standards for each... Every time we're asked to work, for example, 3 times, I had to do some work on my end and check the outlets there and ask why they're plugged into what they should be connected to. In fact, I had to ask everyone who works in that area; check with the lab manager, check with the technical manager, and so on, so I could get as much information as possible. I also had to go and manage things. Sometimes I'd leave at 5 a.m. to see the cleaning lady and how she cleaned the lab, because all of this needed to be known in order to pass on the information to the people so that things run smoothly. Because it's easy when you arrive and work in a department that's already running smoothly, but starting from scratch, I think people don't realize that, and that's the difficulty, and that's also the complexity, because we'll get to the equipment later. You don't learn that in medical school; I didn't even know what a CPS meant, and I didn't know how to write one. So I had to look around and search, and when I called my superiors in Liège, they said, "We can't even tell you what an ICSI microscope is made of."

For them, who takes care of that?

I remember very well my boss in Liège told me, "When I need an ICSI microscope, I call the sales rep who goes to the company, and he brings me the microscope."

I think things must have changed recently because when I ask them when we meet at conferences, or when I went there recently, people say things must have changed a bit, but not with this level of complexity. I'll give you an example: I had a problem only once at the beginning, but it was a good lesson. It cost me 15,000 dirhams, which I never forgave myself for afterward because, for me, it was a waste of money. The supplier, and in fact, he was the only supplier who understood what I was talking about. I was saying I wanted to buy the equipment at a certain point. I tried calling company suppliers because I'd never had any contact with those people. I didn't even know the companies, and I spent a whole day, from 8 a.m. to 6 p.m., explaining to them what I needed.

Is the equipment available in Morocco?

I'll explain how private labs operate. There's an international congress, a European one, that takes place every year in July, and it's held in a different European country. People from the private sector go to this congress, and aside from the scientific aspects and the conferences—there are dozens of conferences at the same time—there's a whole trade fair for equipment, reagents, and everything related to assisted reproductive technology and reproductive genetics. These people from the private sector go there and do business with the suppliers there. If they need small equipment, they can buy it and pack it in their suitcase, and when it comes to large equipment, they process and ship it. This wasn't a problem for the private sector.

Do you attend these conferences?

Well, when I first arrived, I left in June. I arrived in early July. The conference was there, and the team wasn't going to invest in me since I had just arrived. I hadn't yet proven myself, and then there's the fact that you have to register for the conference months in advance, so I couldn't go that year. The following year I had just returned,

so I spent a lot of money. It wasn't possible for me to pay for a conference, but at the time, since we didn't have any way to contact anyone, especially the pharmaceutical companies that supply medications, it just wasn't possible anyway. This is a problem that remains

to this day because gynecologists go always to conferences, but biologists aren't, even though we're normally a team. The problem is that the funding is always given to the gynecologist, but not to us. In fact, the gynecologist I work with sometimes couldn't get funding and had to pay out of pocket. Getting back to the equipment, there was a company that actually understood what I was talking about.

For the ICSI microscope, they showed it to me, and we tried to establish the CPS based on what I knew, but there was one tiny detail.

It was a new experience; you were the first. Did you receive support from the Ministry of Health or the University Hospital?

They didn't know what we were talking about either; it was new for the administration, the supply department. Actually, when I look back, I say we've come a long way, all things considered. The problem is that the lab is the cash cow because, at best, the clinical side has an ultrasound machine that costs 300,000 or even 400,000 dirhams, and they have a table for the puncture and a suction pump; that's all they need. But in the lab, you need an ICSI microscope, a centrifuge, welding machines… a lot of things, so that's really where the big expenses of the funds come in, and that's where you have to know how to describe your equipment without any small flaws. Suppliers are people who do business, and I was still a novice in that sense because I didn't have experience. So I had a big package, I inherited a big package, but I don't regret it. On the contrary, it was a learning experience. Working with Mr. B was a real learning experience. At a certain point, I felt tortured. I would lose track of the summary at 2 a.m. because my mind was thinking in every direction. But it was a very, very good training. There was a lot of things to learn, a lot of sacrifices to make, of course, and you had to work on a lot of areas, so it was quite intensive.

You were able to set everything up completely after all these experiences acquiring equipment. When did you start the center's operations? How long did this preparation phase take?

So after my return, I tried to plan ahead, even though sometimes people said no, it's still too early to do it, but I always think too far ahead. So I told myself I wasn't going to leave things until the last minute, and anyway, it took time. The proof is that I came back in June 2012 and we only started operations in February 2014. It took time because the premises weren't finished yet. The premises had to be properly finished, and then we had to establish the CPS, place orders, receive the equipment, work on the consumables, and then we had to work on the media. When we had to find the media,

we had to see what media we had in Morocco. We had to go see who the supplier was because at the time there was only one, and only one wasn't in Morocco; he was in France, and he came once a month. It was necessary to get in touch with people who would be willing to open doors for you. Even though the private sector sometimes fears having people from the public sector and thinks we're going to steal their patients, we don't have the same objectives at all, we don't have the same clientele because we target the population that can't afford the luxury of private services, so we had to find those people. And actually, finding the facilities wasn't a big problem because we just had to ask the person and receive the facilities that everyone works with. It's only recently that the suppliers have started to multiply. Except that there was quickly the problem of accreditations, and the supplier who brought us the facilities passed away, so he left someone who was shuttling back and forth but who didn't keep up at the right pace. Sometimes he answered, sometimes he didn't, sometimes he came, sometimes he didn't, and I don't know why there's a problem delivering to the public. Before, I understand it's because payments weren't processed quickly, but now we have an extraordinary person in the purchasing department, and she really works miracles because she does everything necessary to ensure that the people who deliver get paid very very quickly, so that's no longer a problem. But now our big problem is the approvals because, for example, last year I lost 1,500,000 dirhams of budget because I had requested a high-efficiency incubator and a benchtop incubator, a multi-chamber incubator, and on the day the tender was to be opened, the suppliers had submitted their bids, but they didn't have the approvals.

Yes, it's the Directorate of Medicines that issues the authorizations; that's for everything. The 1,500,000 was for the major equipment. I needed a high-efficiency incubator and a Multi-chamber incubator. So, in fact, the money was gone. I renewed the contract the following year, and there was a supplier who was able to get approval for a single piece of equipment, but not for the multi-chamber incubator. So, for the multi-chamber incubator, and to avoid losing the money again, I tried to use it for 7 other things that also didn't have approval from suppliers, in order to buy a microscope to set up a cytogenetics structure.

Because these are new companies setting up?

Even if they're established, because, in fact, look, there are the renewals of staff that should happen after a year for major equipment. A year goes by quickly, and they don't even give them a timely response. By the time things are running, in fact, it's already over. And then, for us, the contracts take time to be processed at the level of the university hospital's management, at our hospital's level, and so on. The embryos are still the biggest problem because, in fact, we have suppliers with good culture media that give very good pregnancy results, but no accreditation. There's only one who has their accreditation, but unfortunately, when we worked with about thirty IVF cycles and thawing and transfers, we had zero pregnancies. So we quickly stopped! so what the gynecologist and I did was we bought the culture media from a supplier with our own money. They didn't have the necessary certifications, but they gave us a good pregnancy rate. So that we could continue working, but after a while we started to ask ourselves questions. In fact, I started to doubt myself and everyone on the team

because I wondered if people's habits were changing? I stopped my classes here for two weeks and went to work there. So I thought, well, I'll take advantage of the opportunity while I know what I'm doing. I'll buy the medium myself. And that's what I did. My gynecologist and I pooled our money, and we bought the medium we'd used for good pregnancy rates. We had pregnancies, and that's when we realized there were problems with the old culture medium we'd been using. So that's precisely one of the problems: we're not in a sector where we can work with the lowest bidder!

So what are we lacking to develop infertility services in Morocco?

We're lacking in human resources; it's a real problem!

Now, if we want to move towards the development of these centers, their expansion throughout the Kingdom, tell me, based on your experience, what needs to be done? How can we truly ensure these are successful experiences?

First of all, we need to establish work schedules; what each person should do and what tasks fall under their responsibility. Second, from time to time we need to work on human resources because, especially in the IVF team, I don't have any problems with the clinical side; with the gynecologists, things are going wonderfully well. In fact, that's one of the things that keeps people, me staying at this center, even though it's not something that's common. Often at conferences, you hear people say, "Oh, the gynecologist says it's because of the biologist that there weren't good pregnancy rates." We don't have that problem, but there needs to be work done on the team, the whole team. And there must be a job description system, especially in the lab, because in fact in the private sector the problem doesn't arise because there's a biologist who has their own biology lab and who oversees the assisted reproductive technology (ART) lab, and they have technicians they pay, so it's them who decides what time you come in, what time you leave, what time you do this, you do that, you do that!

In terms of human resource training to have generations who can fill this position and do the work you do today, you see the number of staff trained each year, will it meet the need to have several centers and be able to retain them in the public sector?

Well, the duration can vary because it all depends on the person, whether they are involved or not. I remember when I was in Liège, when there were three egg retrievals, I told them, "You leave the lab and let me work alone so I can see if I can manage the activity if I'm alone in Morocco." Now the technician tells me, "No, I can't manage IVF alone." It's not a big deal; everyone has their own abilities, but what we need to do is at least a minimum of six months. Of course, when you do training, they're not going to give you advanced techniques right from the start.

Does the training of laboratory technicians in Morocco allow them to work at IVF centers?

Yes, no problem. The technician who arrived here was trained on the job. The residents are also trained on the job, and now what I've tried to implement is that I give them mannequin beads, which we call mannequins, and they're like embryos, except they're just plastic and little plastic bubbles.

Do you have the necessary equipment for simulations?

Actually, no! I haven't been able to buy it. When someone comes to show us, for example, to give us a workshop on freezing, I politely ask them.

Here at the university, you don't have a simulation center?

Not for ART.

And how are these people going to learn? Do they have to come to you?

Yes, they're recruited. Actually, I have a resident from Fez in his third year specializing in histology, and we have a first-year resident from the Rabat residency program.

It would be good to have a simulation center that incorporates this component of ART techniques.

Yes, but you also need the money to buy all that. You know how much it costs. An ART lab costs a fortune! So mistakes aren't allowed, but we have a lot of training that must be followed. So, what do we do? We used to have the equipment, but it's only recently that we've been able to get it. In fact, when I first arrived here, but even before or even now, what I ask the residents is to use the discarded embryos, that is to say, the embryos that are not viable, that are not good for transfer or freezing. They can use them, sure, but before moving on to that, I advised them to go through the model beads. Then, in the training process, what we do with the residents is, when we tell them, "You do a certain number, for example, a hundred times," you practice it, and when I see that everything is going well, with the right timing, then you move on to the patient's real embryos. This means there's a certain number of repetitions that must be respected depending on the technique. There are other techniques that require 100 repetitions. There are techniques for people who don't have a natural talent for it; even if they have 100 repetitions, they still won't be effective. So we'll tell them, "Well, you think so? You'll still have to improve." But in teaching, you have to adapt to the learner. The essential thing is to let them feel comfortable, not to stress them out. But they have to make an effort on their end. We can't just be lazy all the time. But generally, things progress gradually when you value people, when you trust them, when you encourage them all the time. I think things go rather well.

What are your recommendations for the development of infertility services in Morocco?

I don't know how to proceed with the DMP approvals, because it is a huge obstacle for me and a huge waste of time because we're constantly working, reworking, and relaunching contracts! In fact, now I'm not really doing any work; I'm just doing administrative tasks. All day long, I'm just ordering the media that will arrive next month because the media are made to order, and their validity period is generally two months maximum, between six and eight weeks. So, when you know how many patients you're going to see next month, you calculate how many of these media you'll need to order, and you have about twenty to order, which requires a lot of work. Secondly, the delivery

of each item requires signing delivery notes, purchase orders, and receiving slips… and all of that is very restrictive, too much paperwork. For each item, you have to sign about twenty sheets of paper, and then when you launch the tenders and the tender doesn't go through, you have to relaunch it and relaunch it, so you have to rework it or you have to review who went through and who didn't, make purchase orders for what didn't go through, and in fact, you start juggling things. I'm not a secretary, in fact, I only do secretarial work; we don't have a secretary at the center.

You accompanied the two other public IVF centers that are starting up in Marrakech and Oujda?

So the people from Marrakech came to us, and I shared everything I could at the time. That was 4 or 5 years ago already! Also the center in Oujda. They made visits to the center and whenever they need something, they contact us.

Thank you very much, that's the end of the interview. I'm going to stop recording now.
