# Supplementary material for: A realist evaluation of the development, implementation and outcomes of the first public ART Centre in Morocco
Source: PLOS Glob Public Health. 2026 Apr 20;6(4):e0005318. doi: 10.1371/journal.pgph.0005318 (PMC13094999; doi:10.1371/journal.pgph.0005318)
Supplement: S2 Data — (ZIP) [file pgph.0005318.s013.zip › S2_Data_Transcriptions_in _English/P4.pdf]

Interview Guide for HealthCare Providers

Participant Code NUMBER: ________P4

1. Can you tell us about your experience at the center, the recruitment process, the start-up, and the training?

For recruitment, we had an interview with Professor B, who was in charge of the university hospital. He approached us and told us about the center and the project, and asked interested people to apply. We had the interview and were selected: two lab technicians and two biologists, a pharmacist-biologist, and the cytogeneticist. So, we went to Belgium from September 2011 to February 2012 for six months of training. The biologists had a year of training. I tried to gather as much information as possible; it was a very, very instructive experience.

Were there internships? Daily practical sessions with the team, and courses?

We started at 8:30 am and worked until 4 pm, so I always arrived at 8 am and was always the first one there. So I did what was necessary at the beginning; I started my andrology training. When they saw that I could perform sperm analyses, morphology tests, and all that, I moved to the IVF side because there were two separate units: andrology and the IVF lab.

For the IVF lab, they had implemented a training plan. The first week, you spend the day observing the team, how they work at each workstation, what they do (preparing the media, ICSI, quality control, etc.). After the first week, I moved on to performing the procedure under supervision, but there was a margin of error; for example, you couldn't exceed five or six uncollected samples. After that, I started working on my own, handling the sample collection, preparing the media, and doing the quality control myself.

How was the start-up at the center in Morocco?

There were some minor problems at the beginning; especially with the administrative staff, for example, the equipment descriptions had to be accurate, so we always had the problem of describing the equipment. We went over and over the same topic quite a few times, the two technicians, the biologist, and the cytogeneticist. There was also the problem of acquiring the equipment, so finding companies that could supply us because the equipment for assisted reproductive technology (ART) is not the same as tthe one used in a biology lab, so they have certain criteria. Also, the building, because the IVF lab, for example, shouldn't have windows but it does require air treatment, so unfortunately, at the beginning, the treatment wasn't done according to ART center standards.

When did you start after your training?

When we returned, the project was already underway, but acquiring equipment took time. We stayed until 2014 and started with artificial insemination. Later, when we

wanted to offer IVF, we had the advantage of a technician who had previously worked at Erasme Hospital and came to support us initially.

What was the business like at the beginning?

We had a steady workload, but initially, the technician and I felt a little uneasy because we were technicians. We had completed six months of training, while they had a year. I had completed six months of training and had performed sperm analyses, sperm retrieval, transfers, freezing, etc.

Weren't the roles clearly defined?

In Liège, the technician did everything. There were also biologists, but everyone can do all the techniques. The proof that I'm capable is that I stayed. I took over everything after the pharmacist-biologist left. I did all the work, and in addition to that, receiving the culture medium, receiving the equipment, coordinating with the pharmacist, and placing orders. And of course, when I needed something, I went back to Ms. H. I was in the lab; I know the order forecasts with all the details.

Didn't you ever have meetings to clearly define everyone's roles?

Yes, we talked about it. We said that we had the same training; the only difference is the ICSI, and here, she didn't even do that, so we've mastered the other techniques.

Are the operations you perform here successful?

Yes, of course, the proof is in the pregnancy results, which speak for themselves with the fertilization rate and the success rate.

Do you review this monthly?

No, unfortunately, but there was a logbook, even though there was a computerized system. We still had the logbook, so every time we had a patient, we recorded it in the logbook. This allowed us to see who was successful. We only discuss this with the externs during meetings.

Do you receive congratulations? Any motivational things?

Yes, small bonuses, but there weren't any incentives, and frankly, personally, I wasn't expecting anything. Between you and me, I wasn't even expecting the bonus I was supposed to receive once a year. I was evaluated like everyone else, even though I've been here for four years. I work alone, I worked Saturdays and Sundays, and I had no family life. I received encouragement from Dr. B and the team. What comforted me most was the joy I saw on the parents' faces when there was a pregnancy and when the mother returned with her baby; it was wonderful!

Administratively speaking, are you like everyone else who works?

Yes, exactly, and you can even ask the team how I was at work. And so far, I'm available. I tell them, no problem, you can call me.

How do you see the operation from the beginning until now? Is the center developing? Is the equipment increasing? Is there an improvement?

There has been an improvement in equipment. But at the beginning of my experience, we were like a baby taking its first steps. The team was well-oiled, yes, but after they left, there was a problem because we have a schedule among ourselves. For example, if you've done this technique, the next one will be my turn, and then the one after that.

So you really do everything at work? And you're the most available?

Yes, in addition to that, I had another responsibility besides the center's expenses. I was responsible for training the technician, but unfortunately, there came a point where there were repairs at the center, so we had to stop for a while. You have to believe in this work to give it your all because when you see the suffering of women—because it's not just lab work —it's perfection.

Because we sometimes had pick-ups on Fridays, which means when we start picking up a woman, we start work a day before, then you follow the first day, the second day, the third day, second or third, it depends, so you follow the progress, the quality of the oocytes, they are good oocytes, you can transfer or freeze them for the patient. Yes, there are criteria, but before doing this operation, you have to do the fertilization. We go through steps beforehand, that is to say, on the day of the retrieval, we receive the sperm. Beforehand, we process it, we just take the sperm. So, in parallel, we collect the oocytes, we put them in a medium, we calculate the quantity, it's a whole calculation because only one sperm can get in.

How can the next generation of healthcare professionals be so motivated and passionate?

Yes, actually, I was like that from the start, even when I worked at Avicenne Hospital. For 11 years, from the very first time, after just one week, he put me on a lab bench because he saw the quality of my work. And when I went to the children's hospital, there wasn't a lab bench; they were only doing tests for pre-operative assessments. So I set up a lab bench for factor VIII and IX, and thanks to that, there was a laboratory at the children's hospital that was twinned with a laboratory at a Hospital in France.

The next generation is doing well. I had some initial problems because I want them to learn as much as possible. We were fortunate to recruit the second technician, who went to Casablanca for training at a private laboratory.

How do you envision the center after your departure? To ensure the center maintains its performance, what is your best suggestion?

We need to recruit at least one well-trained technician with incentives. The availability of equipment and culture media is crucial! Initially, we had a problem finding companies that could supply us with culture media. They needed to have the necessary registrations. At first, we had a budget from Belgium, so we were able to buy the media directly from France, which could deliver to Morocco. Then there was the problem of each item entering the country if it wasn't registered with the DMP. So, for the delivery companies, some have the registrations and some don't. Therefore, they need to make

things easier for those who don't. For example, I'll give you a very simple example: we have different companies. We have one company with their media, and we obtained very good results. On 10 transferred patients, I had a 50% success rate. At one point, the success rate really increased, but unfortunately, sometimes the companies encounter obstacles with their registrations.

Human resources management is truly a crucial factor in success. Mr. B, who manages the center, has a human touch; when there was a minor conflict, we turned to him if we couldn't resolve the problem ourselves.

Thank you very much, that's the end of the interview. I'm going to stop recording now.
