# Supplementary material for: A realist evaluation of the development, implementation and outcomes of the first public ART Centre in Morocco
Source: PLOS Glob Public Health. 2026 Apr 20;6(4):e0005318. doi: 10.1371/journal.pgph.0005318 (PMC13094999; doi:10.1371/journal.pgph.0005318)
Supplement: S2 Data — (ZIP) [file pgph.0005318.s013.zip › S2_Data_Transcriptions_in _English/P5.pdf]

Interview Guide for HealthCare Providers

Participant Code NUMBER: _____P5

If we can talk about your role, could you explain your role at the center?

Well, my role is managing the center. In fact, I was involved with the center from the construction of the center and the implementation of the project. So, I participated as the center manager. It was a project within the framework of the Belgian University Hospital Cooperation, so we worked through all the stages of the project implementation.

So you were already managing health services at the maternity level, so at the beginning of the center design idea, you were part of the team?

Well, when I was assigned here, I was assigned to the Assisted Reproductive Technology (ART) team because they needed a midwife for the project. So, with the team, I also had my colleagues: two laboratory technicians, two doctors, and a biologist and cytogeneticist. We were a group of six to eight people. We completed training in Belgium at Erasme Hospital, and I personally undertook a three-month course in the care of infertile couples and the management of a fertility clinic at Erasme. So, I learned everything related to management, as well as secretarial skills and advanced nursing practice in this field.

It was a very intensive training program, but it was a necessary experience because we were looking ahead to the future of our first public assisted reproductive technology (ART) center in Morocco. So, we participated in the project and tried to gather all the necessary information and obtain condensed training for the transfer of this care here.

It's a pioneering experience, the first public ART center in Morocco. How do you see this experience?

It was truly an adventure in every aspect. First, it's a new field in nursing practice in Morocco. It's not an easy field, a field full of suffering. Setting up the project in the first place wasn't easy; it was complicated because all the procedures were being implemented for the first time, so a whole infrastructure had to be built, regulations and many other things.

So you started from scratch?

From scratch, my first experience in Morocco, my first area of expertise as a nurse, so we worked practically everything. So I participated in every step, supplying the center because it wasn't 100% from the university hospital, it was Belgian cooperation. So the supply of the center, the calls for tenders, and the contracts. Therefore, I participated in everything related to supplying and equipping the center. I was responsible for that and I transferred the training to the nursing team here in Morocco. The training covered everything concerning infertility, couple care, and techniques. So I created a training module for the nurses on how to manage the center, how we would proceed to welcome

couples and integrate them into the care process. So all the logistics, I worked on this with the team, of course, and with Professor B.

How many nurses do you have at the center?

So at first, there were four of them assigned; two male nurses and two female nurses, but then there were only two left, one male nurse and one male nurse. Well, there were constraints at the hospital level, that's why, but we really need the staff. Yes, I trained them to provide nursing care in the field of infertility, so as part of a multi-skilled approach, they even work within the hospital, but in other departments.

The nursing role, the practice, how would you describe it in relation to the care of the couple?

I will tell you, with all modesty, that it is the very heart of the practice, because nursing practice is absolutely essential. The nurse at the fertility center, first of all, is practicing advanced techniques in this field. She is the link between the team, between the biologists, between the clinician, and between the couples. That is to say, she is the one who welcomes the couples, she is the one who assists the doctor, she is the one who liaises with the laboratory and the clinician. Therefore, she participates in all procedures, whether medical or biomedical. She welcomes the couple, she creates the patient file, she communicates with the patients, she simplifies medical jargon into understandable everyday language. In addition to that, she is responsible for self-care because we are teaching the couple, or the woman, how to do it. How to give yourself an injection without the help of a nurse? So the woman is essentially taking charge of her own health. We have the challenge of having women who are illiterate, so we are obligated to teach them how to give themselves injections and administer their treatments. It's tiring, which is why we started offering this nurse consultation from the beginning. It was a unique experience. It's the first of its kind in the fertility center. So the first thing we did here was to offer this nurse consultation. There's the medical consultation and the nurse consultation. It's about establishing nursing protocols so that we're using the same terminology as those used in standard practice. So I documented the nursing protocols, and Dr. B wrote the medical protocols, and the lab did the same. So we implemented the fertility center's protocols.

Didn't this exist before?

This didn't exist before, especially in the field of infertility. It was new, and we did this from the very beginning. For the implementation of this project, we worked with protocols and procedures to facilitate the work and, secondly, to document it for the teams and to train the teams that would come after. The work of nurses who are trained in advanced infertility care is involved in the care process; they are the first point of contact with the couple.

Can you explain this to us?

So, to illustrate this nursing practice, their role, first, when the couple arrives, they are registered by phone. We have a waiting list because there is high demand. Then there

is the contact with the nurse who will welcome the couple, whether by phone or in person.

Is there a website?

Yes, there is an email address, which is the center's location. And of course, there is an email address for correspondence so that people from other cities can easily contact us.

Who handles all of this?

It's the nurse. We don't have a secretary. It's the nurse who welcomes and supports the couple, and who is involved in all medical and biological procedures. The preparation of paper files it is he who will open the initial file, who schedules medical consultation appointments, who prepares the medical consultation room, who communicates with the couples, who will conduct the nursing consultation after the medical consultation. Therefore, he will facilitate the nursing consultation; it is the nurse or the midwife who will explain the treatment properly, inform the patient in simple language, and explain and teach the woman how to administer her injection, support the woman even psychologically or by telephone when announcing the results of blood tests and ultrasounds, and she will communicate the blood test results to the woman and the treatment method. And of course, during the nursing consultation, she will explain and answer all the couple's questions, not only to the woman but also to her husband. Because it is a joint project. This, of course, is the preliminary phase. It involves referring the couple for diagnostic tests that aren't available here at the center.

It's about guidance, support, and communication. And during medical procedures, i.e., IVF procedures, the nurse will be present. So, practically every standard medical procedure involves eight nursing interventions, and even during IVF, the doctor, the anesthesiologist, and the nurse are all involved.

I've explained in great detail to show you that the nurse and the midwife are present throughout the entire treatment process. So, after the IVF procedure, of course, it's the hospital for two days, monitoring the woman, explaining her treatment, and providing all the necessary health information. They also call the woman two days beforehand to schedule the transfer. So, there are several medical techniques involved, and the nurse is always present at reception throughout the process, providing support during the consultation, in the oocyte retrieval room, in the transfer room, and by phone for information.

As a manager, how do you manage all of this?

I manage everything! Well, management first means attending and monitoring activities. Managing the couples, sometimes talking to the couple in most cases because the problem of infertility is a field full of suffering and stress. Sometimes I'm always called upon to participate, even with the nursing team. I support the couples, I support the biologists, and I support the doctors. I have to be present to manage all of this. Human resources management for the center, managing couples, managing material

resources, and preparing activity reports. Following the center's project plan for programming throughout the year, so it's a very demanding role.

Can you tell us about the major challenges you've encountered in managing the center since its creation? With the couples, with human resources, with equipment, and with the information system?

When dealing with a couple, how do we best welcome them? Sometimes the couple comes with suffering; sometimes the husband and wife are in conflict, and when they leave, they even want to abandon the process.

How do couples react to the waiting list?

First, we try to listen carefully to the couple and explain and communicate clearly and simply that there are criteria. When we explain this gently, most accept, and those who are depressed are given priority.

How do you prioritize?

We mainly consider age for example, a woman who is struggling with infertility…

And what about human resources?

Well, with human resources, it's not easy at all, especially at the beginning of my experience. I encountered many problems, which were a very beneficial experience for me, but I always learn from them and try to overcome the problems. The biggest challenges, especially, are the job descriptions—who does what? That's the main issue because it's a new field in Morocco. We need to establish the job descriptions; we don't have them yet, but we're working on it. That's why this difficulty affects me. To avoid conflicts, job descriptions must be available, and even the law exists, but the implementing regulations aren't there. When you're working for the first time in a new center that doesn't have job descriptions, you encounter many problems; it's very difficult! I manage, but it has repercussions on my health, of course. It's not easy. I'm always trying to stay informed, consult the head doctor, see what's happening with the team, and ultimately find common ground to work as a team and listen to everyone.

Do you find the staff motivated to work? Does this field require a particular kind of motivation?

The required profile, especially in nursing practice, is for midwives or general nurses. Both are equipped, but basic training is necessary; you need to know what infertility is. It is also necessary to know how to treat infertile couples and, of course, the logistics, including the information system and the inter-hospital system.

Is there any recognition of human resources? Performance systems?

An evaluation in all hospitals, a criteria-based evaluation, as you know, these are standard. Like everyone else, I always ask that nursing consultations be valued, or even paid for like medical consultations. Because really, given the effort the nursing team makes in this area, it must be valued materially and valued within the system because the majority don't talk about the nursing act, even though it's the nursing act. It's part of

the whole process and it's the key to the success of even the treatment protocol. Because when a couple enters the treatment process, you give them the treatment, but the woman is stressed, and with the stress, the treatment won't work if she hasn't been informed about the protocol and how to follow it. She'll fail, so that's already 14,000 dirhams. So it's already difficult for the couple, and the medical procedure will fail.

Are the 14,000 dirhams reimbursable?

No, not currently, to my knowledge, but there are some organizations that reimburse a little, but it's not widespread yet, though it's being processed.

How do you see the importance of this kind of center in Morocco?

The importance of the power of this revolutionary center, because infertility wasn't a priority in the SR strategy in Morocco. We took a lot of time to tell them, even the Minister of Health, that infertility is a suffering, just like cancer. Moreover, it's increasing, reaching 15%, and infertility means suffering, and suffering means social problems, family problems, and infertility can sometimes be the cause of divorce, the cause of depression… because the core of the family is the children in our society.

Do you think infertility in Morocco is a priority, or do we still need to work on it?

We still need to work on it; we need medical coverage, mental health support, so fortunately, this center is a pioneering center that has found a large audience. We really have a very long waiting list, whereas the private centers that were pioneers, even with their excessive costs, so this center plays a major role in reducing the suffering of couples experiencing infertility in Morocco who face difficulties accessing infertility care and services.

In your opinion, how can the State support this center?

It's true that the cost is already lower than in the private sector, but we need medical coverage because even with the 50% discount, we still need medical coverage. Secondly, we need several centers, so each university hospital should have a public IVF center for each region, and we should also have partnerships with other organizations and a well-defined pathway, a structured system, blood collection points, and high-rise buildings next to hospitals to facilitate accessibility. So this center has already facilitated geographical accessibility and financial accessibility (50% discount), but there is still more to be done. We also need human resources because demand exceeds supply; we don't have sufficient human resources, whether medical, nursing, or even biologists. We also need new talent, training, and support for training, even participation in conferences.

The state must take charge of this center because it is primarily based on training, research, and expertise in its field, because this center has already transferred expertise to other centers in Morocco, such as the University Hospital of Marrakech and Oujda.

When other centers wanted to implement assisted reproductive technology (ART) services, did they come to this center?

Yes, they came and saw how we work. There were other processes that had been initiated, which were difficult; for example, the pricing for IVF procedures wasn't official. So we worked on the pricing with Dr. B and the whole team, we worked on the minimum standards for an IVF center, the protocols adapted to our context, we also worked on the coverage, it's still being processed, even for the reimbursement of medications.

So we were there to provide services to couples and at the same time we were working on other things to facilitate the management of infertility in Morocco and share our experience with other public centers that are starting up.

Also, there are even doctors from other countries who came to see us and gained experience to get started, like this center in other countries. We have even received other african countries patients who had the procedure done here and some pregnancies were successful.

Does the center accept foreign couples?

Yes, we remain open to all couples of different nationalities. The only thing we insist on is that the couple must be married.

Thank you very much, that's the end of the interview. I'm going to stop recording now.
