# Supplementary material for: A realist evaluation of the development, implementation and outcomes of the first public ART Centre in Morocco
Source: PLOS Glob Public Health. 2026 Apr 20;6(4):e0005318. doi: 10.1371/journal.pgph.0005318 (PMC13094999; doi:10.1371/journal.pgph.0005318)
Supplement: S2 Data — (ZIP) [file pgph.0005318.s013.zip › S2_Data_Transcriptions_in _English/P6.pdf]

Interview Guide for HealthCare Providers

Participant Code NUMBER: _____P6

I would now like to begin recording our conversation.

Yes, I agree.

My connection to the project to create the center dates back to my residency training here at the Les Orangers Maternity Hospital. In 2009, I began my training in Belgium as part of the project on couple support and assisted reproductive technologies (ART).

Please tell me about the different fertility care services (e.g., preventative, diagnostic, and therapeutic) available at your center [researcher to facilitate the conversation based on services]. Why are these services important in our context?

Infertility in Morocco is a serious public health problem and comparable to the suffering caused by cancer. Couples suffer greatly to access the appropriate care. They face psychological distress and financial ruin as it is very expensive and not yet covered by health insurance.

Is there basic training in the management of infertile couples and assisted reproductive technology (ART) in Morocco?

Unfortunately, infertility is not well represented in the training curriculum for obstetricians and gynecologists (OGs) in Morocco. We receive extensive training in obstetrics, gynecology, and oncology. However, regarding infertility, we have only one course that covers the basics and is very brief on ART techniques. This is due to the absence of ART centers in Morocco that provide ART techniques at university hospital levels with laboratories. Before our center, these centers simply didn't exist!

This is also important for other specialties, such as urology. Therefore, one of the center's successes is providing training for residents in Morocco in diagnosis, treatment, and ART techniques. And we are also part of training teams for a university diploma in reproductive medicine and biology.

Have you received training in managing infertile couples and assisted reproductive technology (ART)?

Yes

If so, can you tell me where, for how long, and what the objectives of the training were?

I was pleased to be selected after the interviews to participate in the project, along with another gynecologist. I received a year of training starting in October 2009 in Liège and attended courses at the Free University of Brussels during my residency.

What was the situation before the establishment of the ART Center? How did couples access services? What problem did the ART Center solve?

Before, there were no public centers. Infertility services were provided by the private sector, at a very high cost, which remained inaccessible to middle and lower socioeconomic groups. In the public sector, infertility was not a priority and was underdeveloped in Morocco.

What were your contributions to the establishment of the first public Assisted Reproductive Technology (ART) Center?

Upon my return from training in Belgium, I participated in the architectural organization of the structure during its construction. I proposed many ideas to ensure it met the standards of an ART center and to optimize the premises; communication between the lab and the transfer room, and an airlock area in Operating Room.

We also developed a guide to ART center standards with the Ministry of Health because none existed before, and based on our experience, we had encountered several difficulties due to the lack of standards for ART centers. This guide addresses standards in terms of premises, equipment, and human resources (profile, training). These standards are shared during the training we provide in the University Diploma (DU).

During the establishment of the IVF center, did you encounter any difficulties?

Yes

If so, what were they and how did you overcome them?

The center faced many difficulties starting up, as it is a pioneer in this field within the public sector. The first problem was the construction, due to the lack of standards. Then, after operations began, we faced the challenge of meeting precise specifications for purchasing equipment, materials, and medical devices. Also, the lack of suppliers in Morocco who were certified for the essential materials and devices needed for IVF was a major challenge. We tried to overcome this through Belgian cooperation, supplier donations, and collaboration with the private sector. Also, training and motivating human resources is another major challenge for IVF. The cost also poses a significant challenge for couples. Although we offer a package deal at less than 50% of the cost of private services, many couples are unable to afford this amount.

What have been the achievements of establishing the Assisted Reproductive Technology (ART) Center?

We contributed to the development of a guide for the care of infertile couples, in collaboration with the Population Directorate, covering all three levels of care, including primary care. General practitioners also play a role in diagnosis and timely referral. We see the majority of couples who have been waiting for treatment for years and have received inadequate care, and we know how age-dependent infertility is. Therefore, it is important to involve and train general practitioners.

Since the creation of the IVF center, have you contributed in any way to improving the care of infertile couples in Morocco?

Yes

If so, how?

From the outset, we began recruiting couples. We introduced IVF techniques gradually and supported the teams. First, consultations, then inductions, and subsequently inseminations in early 2014. And I wear many hats, as a clinician, the only one working at the center, providing support and therapeutic care for couples. We receive couples who come from very far away and after a long journey. So I oversee their care in all its aspects. We have a hospital information system. I also play an organizational role, coordinating with the various clinical and nursing teams, as well as the laboratory team, since the procedures are closely linked. I am also in contact with the biochemistry and sample transport team at the laboratory. We don't have a medical secretary. Since the center's establishment, we have implemented a special circuit for the Assisted Reproductive Technology (ART) center. After several meetings with all departments to explain the specifics of ART services. I also manage everything related to forecasting and ordering medications, consumables, culture media, and, if needed, equipment and supplies, and ensure compliance. I also manage the weekly medication requests and quantities for the couples receiving care.

Do you think the Center has an impact? What kind?

Absolutely, since its creation, the center has contributed to the development of infertility services in Morocco. Not only through offering quality services at a lower cost, but also through advocating for the prioritization of infertility services, training, research, and expertise in this field.

In your opinion, who are the people most affected (positively or negatively) by the Center? Why? [Context and Probe Mechanisms]

The vulnerable population, from low and middle socioeconomic backgrounds. Our clientele is completely different from that of the private sector.

In your opinion, what factors contribute to the Center's impact? How do these factors ensure that the Center has an effect? In what way? [Probe Mechanisms]

Participation in standards and regulations, training and expertise in infertility in Morocco. Participation in legislation. We paved the way for other public centers; medical devices for ART techniques were not approved in Morocco. We worked with donations in order to be able to provide care. The center has created momentum and mobilized all stakeholders to ensure the availability of care for couples. We worked and then stopped because of the unavailability of medical devices and equipment. And the unsuccessful calls for tenders and contracts.

Regarding the needs, what is your opinion on the availability of public hospitals and public ART centers that can treat infertile couples? [To elaborate, the researcher will point out that most of these services are in large cities and need more services.]

We are the first ART center in Morocco, a pioneering ART center in Morocco. Three new centers are trying to start operations but with many difficulties. Whereas every university hospital should have an ART center to allow couples to benefit from ART services in the different regions of Morocco.

What else needs to be done to increase couples' access to preventive, diagnostic, and therapeutic interventions for infertility?

A clearly defined care pathway is needed, starting at the health center with a trained general practitioner who ensures timely diagnosis and referral, when necessary, to a specialist consultation. Ensuring universal coverage of infertility services is a right. We have contributed to the work aimed at integrating ovulation inducers into medical coverage. But this work must continue. At the center, we have a flat fee that is 50% off, but if this fee were reimbursable, it would be very beneficial in reducing the financial barrier to accessing infertility services.

What are the three most important lessons you have learned from your experience in setting up and subsequently managing the clinical aspects of the ART center?

Qualified, motivated human resources with a very high level of professionalism: This is a job that demands motivation and passion. Staff working in this field must possess specific qualities because they must manage the psychological state of infertile couples with a particular emphasis on confidentiality, given the specific nature of the problem in society. This also applies to laboratory technicians.

Infertility should be considered a public health priority same as other pathologies.

Based on your experience, what recommendations would you make to other low- or middle-income countries if they wish to establish public ART centers?

It is very important that countries prioritize infertility services on the SRH political agenda. We have extensive experience that we are ready to share with interested countries. Political will and support are absolutely essential to integrate infertility into the country's health strategy, as are the necessary financial resources, because these services are expensive. Human resource training is also crucial. Furthermore, it is necessary to work on regulations at the national level and also to secure suppliers of medical equipment and devices. I also recommend public-private partnerships in this area. We also have a role in assisting and supporting new ART centers, and we do so whenever there is a request.

Thank you very much, this concludes the interview. I'm going to stop recording now.
