# Supplementary material for: A realist evaluation of the development, implementation and outcomes of the first public ART Centre in Morocco
Source: PLOS Glob Public Health. 2026 Apr 20;6(4):e0005318. doi: 10.1371/journal.pgph.0005318 (PMC13094999; doi:10.1371/journal.pgph.0005318)
Supplement: S2 Data — (ZIP) [file pgph.0005318.s013.zip › S2_Data_Transcriptions_in _English/S1.pdf]

Interview Guide for Policymakers

Participant Code Number: ________S1

1. General Landscape and Context of Fertility in Morocco

First, I would like to start by asking you a few questions on the general situation in Morocco around infertility.

1.1. How is Morocco as a country addressing infertility?

It is very interesting to document our first ART center in Morocco; it is a pleasure.

Regarding infertility, it's true that the Ministry of Health and Social Protection (MSPS) began developing this component in 2013. This doesn't mean that infertility care didn't exist; it did exist, and it was more developed, particularly for assisted reproductive technology (ART) in the private sector. Since 2013, we have been working tirelessly to develop this component. We began our work by creating a framework for infertility care, and this framework aims to standardize care at the primary care level, i.e., with general practitioners in both the public and private sectors. Especially in our context, the age of marriage has increased, making it a priority to avoid wasting women's time trying to have a child. This is why we prioritized this initial activity: developing a framework for the management of infertility in couples before moving on to ART. So it is a priority for the ministry, it has been included in the sectoral strategic plan of the Ministry of Health.

1.2. Do we have a national policy or a strategic plan to address infertility in Morocco?

Infertility is currently part of the Ministry's sector strategy; it is part of the national reproductive health strategy, which ran from 2011 to 2020 and is currently running from 2021 to 2030.

Infertility is addressed in the Ministry's various strategies, and we also have a national plan dedicated primarily to assisted reproductive technology (ART).

1.3. Which policies and laws regulate fertility care and assisted reproduction in Morocco?
For ART (Assisted Reproductive Technology), from a regulatory standpoint, there was a void; we didn't have any regulations.

Since 2013, the initiative to develop a law has been underway, it's true. It was a first, it took a long time, from 2013 to 2019, and it was only in April 2019 that Law 47/14 was passed. This law currently regulates ART practices, and its implementing decrees are being drafted.

1.4. Is there a national registry and licensing body for fertility care and assisted reproduction?

No, at the moment there is no national registry or licensing body, but it is provided for in the law; it is part of an article in the law, the implementing text relating to the issuance of accreditations. These are not licenses but accreditations, which are provided for in the implementing texts. It is true that we took time to develop these implementing texts, but the structure responsible for regulation was too busy during the two years of COVID-19 2020/2021, which caused significant delays in the development of these implementing decrees.

1.5. Is infertility included as an essential component of Sexual and Reproductive Health and Rights (SRHR) policy and services in Morocco?

The national SRH strategy for 2021-2030 has nine components, and one is dedicated to infertility. Therefore, infertility is an essential component of the strategy and services and rights related to Sexual and Reproductive Health (SRH) in Morocco.

1.6. Do you think that fertility care is important in our setting and why?
Exactly, they are so important, and if the sense of importance wasn't there, we wouldn't be focusing on developing this component, especially since before, we would refer to the WHO data. We have 12 to 15% of couples suffering from infertility. We have studies in Morocco that support these figures; we even included infertility in the national surveys conducted by the Ministry of Health. This is a significant step forward; we included questions related to infertility in the 2018 National Population and Family Health Survey, a first for us, and it yielded a figure of 10.6%. It is what it is, but it's a figure; these are couples who suffer from infertility. Even if the figure is lower than that, we must give it importance as a right. The couple has the right to have the desired number of children at the desired time,

Just as contraceptive methods are made available to the population to space births in order to have the desired number of children at the desired time, the same must be done to help couples have the desired number of children at the desired time.

2. Setting up of the Public ART Center

2.1. What was your role in the implementation of this first public ART center in Morocco?

Yes, for the ART center in Morocco, with Professor B, whom we thank immensely for his commitment to carrying out all his work related to rehabilitation and sexual health in general, and ART in particular. It's true, we were in constant contact with him for follow-up and to support him at any time and in case of need.

2.2. What was the situation like before the first public ART Center was put in place? How were couples accessing services? What problem did it solve?

So it's an honor for us to have a public ART center, which is the Orangers Maternity Center, a national benchmark for us. Before this center, as I said at the beginning, the private sector was very developed; we had around 18 or 19 private centers, while we didn't have any centers in the public sector. This center was the only one before we moved to the private sector. And you know, in the private sector, the financial burden is very real, as is the burden of the procedure itself, and of course, the travel of couples from their regions or provinces to larger areas, especially since private centers were mainly located in large cities. So for small, remote towns, it's extremely expensive, both for medical care and for the care of the woman and her husband in a large city. It was a burden, which is understandable since it's a burden. Couples will have access to it; these are the couples who have the means or the couples who have managed to overcome their significant social constraints to obtain the funds to attempt an IVF cycle. Unfortunately, even if the first attempt fails, they will be forced to try again, and God knows that the majority do not because they lack the resources. This improves access to assisted reproductive technology (ART) for the population, especially for vulnerable populations. This is very important, particularly compared to private centers. The treatment costs much less, and I remember well, since we're talking about financing, I was at a conference. He gave a presentation showing how much IVF costs, and the room was in turmoil. Some people from the private sector left the room; the figure that was announced devastated the private sector. That being said, this center has contributed to improving access for couples who lack the means.

2.3. What steps were taken to ensure that services could be started at the ART Center? [please elaborate]

This center began its work before the official inauguration, and it was ensured that staff were trained in advance, equipment was ordered, and of course, the structure was built. All the technical and human resources were prepared in advance and even tested over a period of two to three years before the official inauguration. The preparations were not easy to launch a first ART center at the public level; it was a very difficult task, and currently, it serves as a model for developing centers at the public level.

Each time, in particular, university hospitals want to establish an ART center on their premises; they generally use the Orangers center, which is the benchmark.

2.4. What policies and regulations were needed to ensure that ART provision was possible at the Center? [please elaborate]

In all honesty, the center began working well before its inauguration; it was the reference point for drafting legislation and establishing certain standards at the legal level; it was a reference center for us in its entirety.

2.5. What are some of the actions and measures that were needed in order to enable the provision of fertility services in Public ART Centers? *[Researcher to probe what action needed to take place in relation to i) Pricing of ART treatments, ii) health coverage of infertility treatments, iii) Marketing Authorization and Registration of culture media and medical devices, iv) standardization of public ART centers, v) development of infertility management guideline, vi) Integration of infertility in Health Plan, and v) Application decrees of Bill N° 47-14 on ART]*

I'll start with pricing, as I mentioned with Professor B. He tried to estimate the cost of the procedure by referring to everything related to the care provided for infertility treatments. Similarly, we advocated with the national health insurance system for the inclusion of infertility-related medications in the list of reimbursable medications. The AMO (Health Insurance Fund) is indeed involved, and Professor B worked with us on this matter.

Currently, a list has been included, and it was the same thing—a first for the moment. We are currently advocating for this, and the work is being carried out by the National Health Insurance Fund (AM) to ensure comprehensive infertility care, not just medications. Similarly, advocacy has been done to obtain marketing authorizations (MA) for medications, and this has been done with pharmaceutical companies to encourage them to submit their applications for consideration. And here's a story: "I was asked why medications aren't reimbursable. I took the question into consideration, and I was at the National Health Insurance Fund (ANAM)." What was the result? The result is that pharmaceutical companies must submit their medications for reimbursement; that is, for medications to be reimbursed, the documentation must be in place. It's not the ANAM (National Agency for Health Insurance) that will tell them to bring in their files. Therefore, the initiative must come from the pharmaceutical companies, and through partnerships with very active NGOs in this field, such as MAPA, we were able to advocate with pharmaceutical companies. As a result, the documentation was submitted, and the medications were included on the list. It was so easy to submit the medications for reimbursement.

The same applies to medical devices. With Professor B, anything consumable was a problem. He launched calls for tenders, but they were unsuccessful because the devices weren't registered in Morocco. Similarly, we advocated with the DMP (Directorate of Medication et

Pharmacy) and with the companies holding the licenses for these medical devices, and now they are registered. So it's thanks to this center that we are standardizing medical equipment, techniques, medications, and infertility care. It's a reference center for assisted reproductive technology (ART), not just the clinical procedure itself, but everything that precedes it. We've also developed a reference guide for public ART centers, but we haven't published it yet, pending the implementation regulations, in case there are any corrections before publication. We used the first public center, and Professor B was our essential partner in developing these ART standards. He was also with us in developing the framework for the care of couples, and then the Marrakech center, which was already established. So both centers were with us during the development of the framework for the care of infertility in couples. For the integration of infertility, it is integrated at the sectoral strategy level, just as it is integrated at the national strategy level for sexual and reproductive health. The implementing decrees are currently being drafted. For the Ministry of Health, our initial objective is to establish ART centers at the university hospital level. Each university hospital must have an ART center, so in principle, for this first center, the budget is essentially allocated to the University Hospital.

2.6. What were the key investments in the health system infrastructure that needed to be made during the setting up and implementation of the Center? *[Researcher to probe what action needed to take place in relation to i)service delivery, ii) health workers iii) health records iv) infertility medicines and equipment) management and leadership and vi) financing and subsidization]*

Standardization of everything related to structure, information support, and medical and technical equipment is essential, but the governance of these centers is also crucial. It is very important to have dedicated staff for these centers because it is a fascinating job that requires qualified and passionate personnel.

2.7. How is the setting up of the provision of public fertility care at the Public ART Center financed? Please elaborate.

It's part of the university hospital, I think there was even staff from the Ministry of Health involved through international cooperation.

3. Contributions and Outcomes of the Public ART Center

I would now like to focus on the events since the creation of the first public ART center.

3.1. What difference do you think the ART Center has made to people with infertility? Why so?

This ART center has been able to improve accessibility for couples who lack the means; it has been able to fulfill a couple's right, which is very important if we consider a legal factor, especially when it concerns young couples experiencing infertility in a situation of suffering.

3.2. Who do you think is benefiting from the Center? *[Researcher probe if the Center is benefiting people from all regions, social economic status, ethnic or religion etc.]*

In principle, for this center, at the very beginning, when it was the only public center, it was the population of the entire kingdom who used it. Once we had the Marrakech center, which was established, the population of the southern zone, the Marrakech-Safi zone, was served by this center. Currently, we have the third center in the eastern region. Oujda has started to take care of couples from the region with the establishment of other public centers. This will certainly relieve these centers, whose waiting list is very long.

3.3. Why do you think the ART Center is mostly benefiting these people?

It benefits everyone, but especially those with fewer financial resources. An important point we appreciated about this center is the support provided to clients.

It has qualified nursing staff who accompany and support these couples before, during, and after treatment. This is an aspect that is lacking in private centers. The psychological aspect is very important for the success of the procedure itself. Regarding home treatment, they can manage this aspect themselves.

3.4. In your view, which factors are contributing to the Center having an impact? How do these factors cause the Centre to have an effect? In what way? *[Probe Mechanisms]*

The essential foundation is always HR. The Center's resources and leadership are always engaged in monitoring patient care and supporting staff. Staff who love what they do. Essentially, regarding staff, having equipment, etc., is very easy; it's not what's lacking. What's lacking are clinical, managerial, and interpersonal skills. This is very important.

3.5. In your view, what factors can potentially prevent provision of fertility care services for men and women with infertility at the Center? What should be done about these issues?

There are several of them, but what can hinder things most is the staff shortage. Personally, I really appreciate this center, but I always have a concern about the staff shortage and the sustainability of the center's activities. We must have sufficient trained and qualified staff to work at this center. We must always think about preparing the next generation; it's very important. As long as the right people are there, we can't wait for people to leave to get others. Human Resources must invest to ensure quality service. Similarly, there's an area that needs development: providing information to couples about raising awareness and preparation well before they begin the process. Even though the Ministry of Health hasn't yet reached out to the general public, the center has a team that does this, and it's very important. This team ensures understanding well before couples begin their fertility treatment process. And of course, another factor is not bringing only Cases requiring ART are not all cases of infertility, and that's why we developed a care framework to avoid overloading this center with cases that require care well before they require ART, but which can be managed within the healthcare system long before reaching this center.

3.6. Compared to existing need, do you think that the Center is meeting the needs of fertility care in Morocco? What else should be done?*[Researcher to probe further, researcher might point out that the Center is in a large city. How does this affect rural population? Are the number of ART Centers adequate?]*

Currently, the Ministry of Health's vision is to establish an ART center at each university hospital. We have 7 university hospitals; if we manage to have 7 ART centers, I believe that's a first step and a gain. Once we have public centers at the university hospitals, the next vision is to establish an ART center at each regional hospital to further improve accessibility, but always with support. We must have qualified staff to manage these centers. Having a structure is easy, but we need qualified staff to work in this center. It's true that university hospitals are located in large cities, but for a first step, we don't have a choice but to gradually move through university hospitals before going to regional centers.

3.7. In your opinion, does the ART Center play any other role in fertility care provision? Which one? [researcher probe referrals or training of health professionals]

It's the support and preparation aspect before and during the preparation of couples.

This is very important, and the center's team reinforces the implementation of self-care at home. This is a very important component that relieves the center on the one hand and contributes to the success of the procedure. Also, training to train other people—we have guidelines, except at the Ministry of Health. We are still working with Professor B to establish a training plan in partnership with the private sector. Training in the public sector is insufficient, but this center will contribute to the basic training of specialist doctors to do many things. We will always establish a partnership with the private sector, which is much more developed in our country, to ensure the sustainability of training at the Moroccan level.

3.8. What are the reasons why the development and implementation of the ART Center has been successful or not?

It's about committed leadership who is convinced of what they are doing. It's important to go slowly; it's normal, it's a first project, and to overcome obstacles, and each time there are obstacles, he sits down with his team around a table with resources from the Ministry of Health to discuss the obstacles and find a solution. So, patience is necessary. It's the main reason for the success of this center: patience, leadership. In leading another person, we should have the same result.

4. Perspectives on learning from Morocco to other countries

I would like to conclude by asking you what has been learned in Morocco and how this can be used to help other countries begin providing fertility care in public hospitals.

4.1. In your opinion what would be the benefits, if any, to the implementation of a publicly funded ART Center in another country?

It is to improve couples' access to the treatment of infertility problems; that is the essential reason. It is to improve accessibility; there is a whole host of suffering, a social aspect, a human right. It is very important, and the majority of countries are committed to meeting the need for this human right. It is very important.

4.2. In your opinion, are there obstacles to the development of public ART Center? If so, which ones and how can these be overcome?

Once the comprehensive infertility care component and assisted reproductive technology (ART) are included in the orientations and strategies of the Ministry of Health, I believe that the obstacles must be overcome, including the financial aspect, and even in terms of international cooperation.

If we have structuring projects, cooperation generally supports this; it's simply a matter of having structuring projects. The Ministry of Health is now ready for the construction of university hospitals (CHU) with the budget; it's true that the first center was supported by cooperation. Now the other centers are being built with the university hospital budget, and the acquisition of

equipment and consumables is part of the budget, but the training component remains and needs to be strengthened.

4.3. What other considerations do you think should be taken into account if/when introducing such public ART Centers in other low- and middle-income countries?

It is very important to have reference points, to have standards, in order to move towards implementation and expansion. Standards regarding the architecture, organization, and operation of the regulatory center must be established. It is necessary to regulate the practice of ART from the outset. Standards must be in place before implementation, and of course, advocacy and awareness campaigns must be conducted from the beginning. Countries must be made aware of the importance of this component of infertility care. It is very important that they are responding to human rights; it is not a luxury, it is a response to a right of the population.

Thank you very much, that is the end of the interview. I will stop the recording now.
