# Supplementary material for: A realist evaluation of the development, implementation and outcomes of the first public ART Centre in Morocco
Source: PLOS Glob Public Health. 2026 Apr 20;6(4):e0005318. doi: 10.1371/journal.pgph.0005318 (PMC13094999; doi:10.1371/journal.pgph.0005318)
Supplement: S2 Data — (ZIP) [file pgph.0005318.s013.zip › S2_Data_Transcriptions_in _English/S2.pdf]

Interview Guide for Policymakers

Participant Code Number: ____S2

1. General Landscape and Context of Fertility in Morocco

First, I would like to start by asking you a few questions on the general situation in Morocco around infertility.

1.1. How is Morocco as a country addressing infertility?

The general landscape of infertility, especially the demographic landscape, as studies and surveys have shown, and the figures today demonstrate a Fertility Rate of 2. The child, or newborn, has become very precious, and Morocco is tackling infertility with all scientific means. It simply responds to the needs of the couple; there is a whole health policy. First, births must be spaced out, women's health must be well taken care of, and morbidity and mortality must be reduced. It's a whole public health program. And then there is assisted reproductive technology (ART). Technology, in a broad sense, allows for medication, therapy, and the development of medicine and science. Today, techniques allow couples to have children. And then there's the law. It's a law that responds to the need and protects the couple and the healthcare provider, that is, the doctor. It's a law that respects ethics and professional conduct.

1.2. Do we have a national policy or a strategic plan to address infertility in Morocco?

1.3. Which policies and laws regulate fertility care and assisted reproduction in Morocco?

1.4. Is there a national registry and licensing body for fertility care and assisted reproduction?

The law provides for this national registry and all the associated bodies, and the law is very clear. I think it's time to draft the implementing regulations to continue this path toward the development not only of ART but also of SRH in general. Of course, infertility is included as an essential component of the strategy, services, and rights related to sexual and reproductive health.

1.5. Is infertility included as an essential component of Sexual and Reproductive Health and Rights (SRHR) policy and services in Morocco?
Yes, there is the second SRH strategy 2021/2030, which includes all components and SRH in the broadest sense.

1.6. Do you think that fertility care is important in our setting and why?

2. Setting up of the Public ART Center

2.1. What was your role in the implementation of this first public ART center in Morocco?
There is a whole team that worked together in a very close and inclusive way, and there were several programs that converged their ways of thinking and their efforts, and we worked first with the private sector, then there were many consultations between healthcare providers, between lawyers, between sociologists, and between university professors, since they had this essential responsibility of being able to train future providers of assisted reproductive technology (ART). There were a whole series of draft meetings, since we worked several times until 2 or 3 a.m. Those were hours on end to be able to agree on a piece of legislation. We had lawyers with us who guided us because they had done a benchmark study with other countries, and we were able to have a text that responds to the Moroccan context. And the role, as I said

earlier, is not individual but is a role of the whole team, which today is proud of the Moroccan product that meets the needs of Moroccan couples and The university professors were with us and took on this essential responsibility of training future providers.

2.2. What was the situation like before the first public ART Center was put in place? How were couples accessing services? What problem did it solve?

I can't be very precise because there wasn't much information, no registry. Were there doctors who practiced ART? Were there couples who visited these private doctors? Were there couples who were satisfied, who had more than one child thanks to ART? But today we are in the 21st century with all the technologies, globalization, it is necessary to regulate this practice because we are not immune to abuses, and the state, the public authorities, have reacted by taking this regulation of the practice into consideration for the benefit of Moroccan couples.

2.3. What steps were taken to ensure that services could be started at the ART Center? [please elaborate]

All measures were taken because they were stipulated by regulations, and today I believe that healthcare providers in Morocco are responsible and conscientious, and they spare no effort to first take the prescribed measures and then respond to couples within the framework of ethics and professional conduct. To my knowledge, there are currently no complaints that this center offers substandard services or services that do not adequately meet needs.

2.4. What policies and regulations were needed to ensure that ART provision was possible at the Center? [please elaborate]

First, there was the program here at the Ministry of Health. They made sure that this was first included in the five-year plans and the budget law. They ensured that this was done. Then there was the cooperation with the United Nations agencies, specifically UNFPA and WHO, which provided us with assistance. I want to emphasize that the law and regulations came about thanks to this cooperation in a very short and reasonable timeframe. It didn't take much time. These people, the stakeholders and decision-makers, were informed, and this consultation process allowed us to reduce the delays and finalize the text.

2.5. What are some of the actions and measures that were needed in order to enable the provision of fertility services in Public ART Centers? *[Researcher to probe what action needed to take place in relation to i) Pricing of ART treatments, ii) health coverage of infertility treatments, iii) Marketing Authorization and Registration of culture media and medical devices, iv) standardization of public ART centers, v) development of infertility management guideline, vi) Integration of infertility in Health Plan, and v) Application decrees of Bill N° 47-14 on ART]*

I think today we are extremely fortunate that the ongoing overhaul of the healthcare system has allowed the texts to be passed by the government council and then voted on by parliament. Law 6.22, published in the official gazette, thus encompassing all these legal frameworks, allows for the overhaul of the system with an exceptional product: universal health coverage. Today, all Moroccan citizens will be covered, especially couples. Assisted reproductive technology (ART) has taken its rightful place within this universal health coverage. The law on ART, which we mentioned earlier, defines infertility as an illness. Therefore, it is the law that legally obligates us to recognize infertility as an illness.

ART will be covered by the Universal Health Coverage (CMU). I believe that this will prevent any shortcomings, since it is covered. So, what is needed at this stage is to train more gynecological healthcare providers to allow couples easy access to ART. I think that the first public center that has been inaugurated, as well as private centers, have a vital role to play in training and providing these services to couples who desire them.

2.6. What were the key investments in the health system infrastructure that needed to be made during the setting up and implementation of the Center? *[Researcher to probe what action needed to take place in relation to i)service delivery, ii) health workers iii) health records iv) infertility medicines and equipment) management and leadership and vi) financing and subsidization]*
The main investments, which in my opinion (and this is just my opinion), were financial, but were necessary but are not really the key factor for success. The real investment is brainpower, human capital, healthcare providers, and especially university professors who have trained in ART (Assisted Reproductive Technology), and I would say that this must continue, and there will be a cascading training system between the public and private sectors, and we will train more healthcare providers, and this can only be a service provided to couples who desire this ART.

2.7. How is the setting up of the provision of public fertility care at the Public ART Center financed? Please elaborate.
The University Hospital is an autonomous institution; when it wants to create a new care unit, it submits it to the board of directors. Generally, when it serves the public, it is approved, and then investments are managed from a managerial perspective, but there is also the state. When the University Hospital doesn't have enough money to do it and it's a service for the public, the state provides a subsidy. I would say that any subsidy, whether tangible or financial, can only succeed if there is an intangible subsidy, i.e., a combination of tangible and intellectual contributions from the university hospital, the Ministry of Health, and United Nations agencies. This is necessary to bring the project to fruition, not in its material dimension, but in its operational aspects, so that it results in a product and the machine runs smoothly thanks to these intangible efforts.

3. Contributions and Outcomes of the Public ART Center

I would like now to focus on events since first Public ART Center was set up.

3.1. What difference do you think the ART Center has made to people with infertility? Why so?

Today, when infertile couples approach an ART center or provider, it's not simple; they arrive in a state of silent distress, so the provider asks them to undergo tests and follow the scientific process. This distress gradually diminishes their sense of helplessness, and when they are given an appointment for ART, this distress transforms into hope. So, today, for couples, these centers represent an unexpected hope, something that was the case ten years ago. And I've met infertile couples at many events, and I've seen their smiles. They were very satisfied. Despite the shortage of centers, they make the effort to travel. So, the action is to establish more centers to reduce the obstacles to accessing this service.

3.2. Who do you think is benefiting from the Center? *[Researcher probe if the Center is benefiting people from all regions, social economic status, ethnic or religion etc.]*

The center's services benefit everyone: couples first and foremost as the main clients, teachers, and service providers thanks to continuing education, an expertise that is being developed. It benefits the country because today, anyone who has a child through assisted reproductive technology (ART) will benefit from all possible care and attention, and will have the opportunity to go to university, enter the workforce, and create value and wealth for their country. Everyone wins.

3.3. Why do you think the ART Center is mostly benefiting these people?

That's a very simplistic view; it doesn't only benefit these people, but it benefits the entire population and the development of the country.

3.4. In your view, which factors are contributing to the Center having an impact? How do these factors cause the Centre to have an effect? In what way? *[Probe Mechanisms]*
First, there is the financial factor in its two dimensions: the solvency of couples. I think Universal Health Coverage has solved this problem, this insufficiency. Couples are solvent; they can be covered.

There is also the second aspect of solvency: This center must be maintained in harmony with the development of medicine and technology. Therefore, there must be investments so that this center evolves in a way that is compatible with science and technology.

There is the geographical obstacle: These centers must be present in at least the 12 regions of the kingdom so that people who are not financially well do not have to travel. And the third factor is the continuing education of providers to keep up with the development of science and technology, therapeutics, and medications to increase skills and performance.

3.5. In your view, what factors can potentially prevent provision of fertility care services for men and women with infertility at the Center? What should be done about these issues?

Today, with globalization and open borders, I think it's essential to be very close to scientific training. The factors that can hinder access are cultural: couples will initially turn to individuals claiming to have the ability to treat their infertility, whether it's a traditional healer or a herbalist. This is very culturally ingrained. Therefore, we need to move beyond this and towards scientific training, starting with the family doctor, the primary care physician at the health center, or the private practice, who will then refer them to the specialized center.

3.6. Compared to existing need, do you think that the Center is meeting the needs of fertility care in Morocco? What else should be done?*[Researcher to probe further, researcher might point out that the Center is in a large city. How does this affect rural population? Are the number of ART Centers adequate?]*

It's true the center meets the need, but when there is only one center and infertile couples are numerous, the center is forced to schedule appointments and maintain a calendar. So it meets the need, but within certain time constraints, there should be several centers to increase the availability of care.

3.7. In your opinion, does the ART Center play any other role in fertility care provision? Which one? [researcher probe referrals or training of health professionals]

First, there is its classic role, the continious training, therefore one of the national and international influence of the

performance level of our healthcare system, of our healthcare providers, and all of this is directly linked to its mission. The most important role is hope for all couples, and this is very important for the individual, for humanity.

3.8. What are the reasons why the development and implementation of the ART Center has been successful or not?
ART is in development and has been successful; there is no doubt that to date there are no obstacles to providing care. The center is here, it is responding, but the factor is that efforts have been combined. There is a broad consensus among all public and private stakeholders, whether they are providers or public health planners. It is thanks to this broad consensus.

4. Perspectives on learning from Morocco to other countries

I would like to finish off by asking you about what has been learnt in Morocco and how it can be used to assist other counties to start provision of fertility care in public hospitals.

4.1. In your opinion what would be the benefits, if any, to the implementation of a publicly funded ART Center in another country?

To give hope to other couples beyond our borders, never back down from such a thing, to give real hope, and then the center represents a transfer of skills; it will allow our healthcare system to shine and contribute to sustainable development by investing in human capital to serve all citizens of the world.

4.2. In your opinion, are there obstacles to the development of public ART Center? If so, which ones and how can these be overcome?
I'll repeat my earlier answer but from a different angle. If success were a consensus among all stakeholders, I think the obstacle is not having that broad consensus; it's very far off. And then there are the healthcare providers, both public and private, who are motivated and want to practice this technique. I think the obstacle would be not giving them this opportunity and this chance. Money isn't an obstacle because today there's a right to health enshrined in the constitution and the law, and public authorities never hesitate to provide services to the citizen.

4.3. What other considerations do you think should be taken into account if/when introducing such public ART Centers in other low- and middle-income countries?

The key consideration is Universal Health Coverage and the management because it's expensive, especially in low-income countries. People don't have resources and it costs a lot, and it becomes a hope, and it's an effective technique, it becomes a hope that we can't reach, why? Because of money. I think we need to introduce a support mechanism. Ideally, it would be universal health coverage (UHC), for people who can afford it, that's ideal. If this coverage is still being implemented, there are still obstacles. We need to find a support mechanism for these people to whom assisted reproductive technology (ART) gives real hope.

Thank you very much, that is the end of the interview. I will stop the recording now.
