# Supplementary material for: A realist evaluation of the development, implementation and outcomes of the first public ART Centre in Morocco
Source: PLOS Glob Public Health. 2026 Apr 20;6(4):e0005318. doi: 10.1371/journal.pgph.0005318 (PMC13094999; doi:10.1371/journal.pgph.0005318)
Supplement: S2 Data — (ZIP) [file pgph.0005318.s013.zip › S2_Data_Transcriptions_in _English/S3.pdf]

Interview Guide for Policymakers

Participant Code Number: _________S3_______

1. General Landscape and Context of Fertility in Morocco

First, I would like to start by asking you a few questions on the general situation in Morocco around infertility.

1.1. How is Morocco as a country addressing infertility?

Thank you for these questions. In Morocco, the management of infertility has undergone significant development, but with a number of limitations as well. To summarize, there has been progress that allows infertile couples in Morocco to receive care using existing techniques, both in terms of diagnostics and treatment. That's the positive side. The areas that need improvement, which represent some weaknesses in the management system, include coverage of infertile couples and unequal access to care.

This is not equitable for everyone. I'll elaborate; whether from a historical perspective or in terms of the development of healthcare in Morocco over the last 20 years, there has been a significant increase in the number of infertility treatment centers. These centers are mostly in the private sector, which was the pioneer in this field initially, and there are few in the public sector. This needs to be put into perspective because infertility is a relatively new phenomenon. Only about four decades ago, the first techniques were developed. The first IVF procedure, the first assisted reproductive technology (ART), dates back approximately 42 years. However, there hasn't been widespread adoption of this technology, even in terms of format or healthcare structures. The healthcare system wasn't prepared to expand infertility treatment within the public health system. The SSPs (Social and Professional Services) became interested in contraception because it's something that has developed, but infertility was already addressed within the public system. The private sector took matters into its own hands, and in 2008, the Maternity Hospital of Les Orangers took the initiative to create the first public Assisted Reproductive Technology (ART) center. This initiative was strategic because it was part of a comprehensive project and established the starting point for the overall management of infertility, including ART. So, that's a bit of what happened in terms of development. To summarize, the public system was slow to adopt it. The Maternity Hospital of Les Orangers played a pioneering role in introducing this into the public sector, and subsequently, the University Hospital of Marrakech followed the 2025 plan established by the Minister of Health, which stipulated that each university hospital must have an ART center. We were pioneers in this field and we tried to share this knowledge with others. The SSPs (French Society of Pediatrics) were unable to introduce this primary infertility care in assisted reproductive technology (ART). What is the weakness?

The weakness lies in the geographical and financial inaccessibility of infertility care.

1.2. Do we have a national policy or a strategic plan to address infertility in Morocco?

The Population Directorate's program, the SMI (Maternal and Child Health Service), and the FP (Family Planning) program have introduced the management of infertility. They have done a great deal of structural work, including developing guidelines and preparing standards for assisted reproductive technology (ART) centers. The maternity hospital developed these standards in collaboration with the Ministry of Health. Most importantly, a plan for the development of infertility management and ART in Morocco for 2020-2030 has been developed.

This document was developed using the expertise of the Orangers Maternity Hospital, as we contributed our expertise to its creation. It is available and is at the Ministry of Health. The problem lies in its implementation and deployment. It's true that the two years of COVID slowed down all the plans they had in place in general because there was a global crisis, including in Morocco. But we are already in 2023, meaning three years have passed since its development was delayed, and there is no concrete budgeting for its implementation that is available globally and broadly and addresses all aspects of developing ART in general.

1.3. Which policies and laws regulate fertility care and assisted reproduction in Morocco?

Morocco has made a great effort to develop, validate, and introduce the laws concerning infertility care and assisted reproductive technology (ART). It took a long time and was fraught with difficulties.

But this law was finally passed in 2019. It was necessary because there were many issues at stake regarding infertility care, and finally, we have this law. Now, to implement it, many implementing decrees are needed, and there is practically a decree in place. What is lacking now is the implementation of this law.

1.4. Is there a national registry and licensing body for fertility care and assisted reproduction?

It exists in the law, but it's not yet implemented. The law only exists on paper; there is a register and regulations governing the practice of infertility care, but this law is not yet in effect. So it exists on paper, but not yet in practice.

1.5. Is infertility included as an essential component of Sexual and Reproductive Health and Rights (SRHR) policy and services in Morocco?

It is included in the SRH strategy, but it only occupies a small part. It is not very developed, and it has been said that this infertility care program is an orphan program, relegated to a secondary role. Priorities include maternal health, cancer, and maternal mortality, but infertility is always relegated to a secondary position and considered only secondarily. It seems like an orphan program. If in the definition of essential that the essential means that have been put in place for this the budget.

1.6. Do you think that fertility care is important in our setting and why?

Yes, it is very important for two reasons. Because subnational and Moroccan studie show that couples suffer with a level of suffering comparable to that experienced by cancer and that fertility care requires support; secondly, because the studyconducted here at the maternity ward shows that couples suffer from psychological problems, depression and related issues. This explains the importance of fertility care.

2. Setting up of the Public ART Center

2.1. What was your role in the implementation of this first public ART center in Morocco?

The role we played was that of being visionary, introducing it at a time when it was not a priority. That's the role we played, both with regard to stakeholders in Morocco and with regard to international officials within the framework of cooperation. When we decided to do it, it was with a vision: we said that in 2008, Morocco would face the problem of infertility within the next 10 years. This would emerge because certain program areas, such as maternal health, would develop, maternal mortality would decrease, and positive things would be developed, such as

cancer care and STI management. These areas are well-developed, and infertile couples are becoming increasingly apparent. This was going to create a conflict. In 2008, we proposed this project with the help of bilateral cooperation, which was not accepted the first time because They said it wasn't a priority, neither the second nor the third time. We made a sustained plea, projecting them ten years into the future. That's why the role we played was one of vision and project development, because it required a certain level of technical expertise. We also played a role in implementation, and we were able to execute it on time. Within four years, we were able to set it up and disseminate our expertise through the various requests that came from other university hospitals.

2.2. What was the situation like before the first public ART Center was put in place? How were couples accessing services? What problem did it solve?

Before, the only players in infertility care, up to and including assisted reproductive technology (ART), were the private sector. This meant there was no regulator; that is, there were no rules or standards.

People received care, but under the conditions of the private sector, which didn't play a regulatory role.

When ART was introduced into the public sector, we were able to establish standards, even in the drug supply chain, its purchase, marketing authorization, and all that.

We played a role in ensuring that medications were available, and they were no longer smuggled in from the north. Things became more regulated, and we also played a role in drafting the legislation, taking into account the activities and, above all, the financial aspect.

We set a ceiling on the cost of care; that is, we limited expenses, the cost of treatment techniques, to a ceiling that serves as a benchmark.

That's the role the public sector played, and in general, the public sector plays a role of regulator to counterbalance the private sector, which also has its own model. There was only one model, the private one, meaning you had to pay for the service. Those who couldn't pay had no other alternatives and had to pay the private sector price, which is twice as high. The problem that has been solved is increasingly strategic in relation to the private sector, also a complementary role to the private sector, showing society and politicians that it is possible in the public, because before they didn't believe it, and it required a great effort from the entire team who worked on it because the team working at the IVF center made a great individual and collective effort to demonstrate to politicians, the social sector, civil society, and infertile couples that it is possible to do it in a high-quality way at a limited cost. That's a bit of what was contributed.

2.3. What steps were taken to ensure that services could be started at the ART Center? [please elaborate]

We started with the principle that when an infertile couple comes to the center we work with, we created a model where the couple pays a flat fee and all procedures are handled within the service. This is because the patient is torn between the medications they need to bring and the consumables, between this and that. The center played a role in relieving the couple of the burden and difficulty of gathering everything needed for a procedure. Our model was also based on the fact that they come, they pay, and the center takes care of everything that is cumbersome to manage. The center played a role in obtaining medications and culture media

that weren't even approved for use and weren't even available. We played a significant role in ensuring that the media received approval. We paid a lot to make this system work, even sometimes incurring losses. That's the role we played, and all of this was done within a cost-control framework that the system absorbed. Many disadvantages of a comprehensive care plan in a single geographic location; he pays and he had everything gathered in one clinical location.

2.4. What policies and regulations were needed to ensure that ART provision was possible at the Center? [please elaborate]

Here too, it's theoretical, but practical. Here in our center, we started by establishing the norms and standards of practice. The team here at the center, before starting, became aware that quantity is not a luxury in an ART center. They put the protocols in place first. Before starting, that's what was done strategically. Strategically, to ensure that the work is done correctly, the standards for an ART center were drafted. The center worked with the Ministry of Health. The document is there; it's among the rules, but this document is not yet in effect. We have to wait for the legislation for its implementation. So, there are things that we have established through our own responsibility to carry out activities according to quality standards and protocols, but at the national level in Morocco, it was the legislation that could establish it.

2.5. What are some of the actions and measures that were needed in order to enable the provision of fertility services in Public ART Centers? *[Researcher to probe what action needed to take place in relation to i) Pricing of ART treatments, ii) health coverage of infertility treatments, iii) Marketing Authorization and Registration of culture media and medical devices, iv) standardization of public ART centers, v) development of infertility management guideline, vi) Integration of infertility in Health Plan, and v) Application decrees of Bill N° 47-14 on ART]*

The actions and measures necessary for effective patient care include, firstly, staff competence. Staff must have the necessary skills and adequate training because it is a highly specialized skill, whether it be for the doctor, the nurse, or the manager. Secondly, there must be an organized center, and we have already discussed how it should be organized: no errors, no problems with the information system. It must be well-organized. Thirdly, there must be the resources: high-quality equipment, consumables, and medications. So the ART center is a reflection of a fairly advanced organization that brings together several elements to make a healthcare system work. Therefore, infertility is the combination of several elements so that it can produce a result. It's a team effort; it's different from vaccination, where things are done externally. Here, it's really very specialized and requires a quite complex organization, where, in addition, there's the laboratory and the clinician. If I make a comparison with the maternal mortality program, there's a team that has to manage complications of maternal health, such as complications during childbirth, a team that includes a clinician, a biologist, a hematologist, a surgeon, and a radiologist to be able to manage it, and it's that complex. There are also the laws, but there's also the governance for the implementation of the laws. The law is there, the practitioner, but there is work between the two.

For the management framework to be available, it was created without a request.

It was done before starting. There was a conscious effort to write a framework and take responsibility for this action, which was indeed wise. Regarding the integration of infertility care into the health plan, we fought for this, but the actions are timid. People still think that infertility is not an illness. There is a lot of advocacy work from learned societies and NGOs, and from our

center, which played a major role as a collaborating center. This is part of its project on infertility; it played a major role in moving in this direction.

2.6. What were the key investments in the health system infrastructure that needed to be made during the setting up and implementation of the Center? *[Researcher to probe what action needed to take place in relation to i)service delivery, ii) health workers iii) health records iv) infertility medicines and equipment) management and leadership and vi) financing and subsidization]*

For a public center, first of all, the investments that were made, the management expertise, it was necessary to manage a project, and to manage it, the entire administration played this role.

The university hospital administration supported this project. There was bilateral cooperation that helped us with half of the financing, and for the expertise, all these elements were there for the administrative side. The financial side for the university hospital was the project itself; there is the project content. I'll continue with the investments: 1. Expertise 2. Construction. There was staff training; the team traveled for 6 months to a year, depending on their profiles. We needed doctors, biologists, and technicians for this training, and the purchase of equipment was also a major investment. The maternity ward played a key role in getting this project up and running, adopting it, and ensuring its continuity. The project was completed in 2012-2013 after four years, and subsequently, it was firmly established. The maternity ward took ownership of the project to ensure its long-term viability. This is the investment that was made. It even included the hospital's budget, that's right.

The investment includes the team's leadership; there are key people who played a role and who contributed their expertise and skills to manage the center. There is also the leadership at the maternity level; this is the role I played to maintain a position, autonomy, identity, and sustainability, and also to integrate activities at the gynecology and obstetrics care level. So the integration was achieved, therefore there was this dual leadership.

2.7. How is the setting up of the provision of public fertility care at the Public ART Center financed? Please elaborate.

It was initially funded by the project itself. After the project, there is autonomy with the care provided and the hospital budget. We try to achieve a balance between expenses and revenues, knowing that a rebalancing will be necessary. The costs of the techniques have increased, and after ten years, we want to review and correct the costs so that we can stop the budget rebalancing.

3. Contributions and Outcomes of the Public ART Center

I would like now to focus on events since first Public ART Center was set up.

3.1. What difference do you think the ART Center has made to people with infertility? Why so?

First, it provided them with care in a complete, holistic model, as I said earlier. In a single geographical location, there is testing, care, medication, culture media—everything is available in the same center, providing comfort to the couple.

2. A psychological approach by the on-site team, which implemented the psychological approach that was superfluous at one point in other centers, but here it was established.

3. It brought a more structured role for self-care, i.e., the couple participates in their care through self-injection, and this required staff training. All of this is for care; there is always a contribution. What have we brought to couples? We have reduced financial inaccessibility, but with some margin, i.e., couples could pay $5,000 or $4,000; here they pay only $1,500 for access. So we have reduced the market segment of couples to access this care, and it is still expensive in terms of financial care, comfort, and quality of care, but we couldn't do this for everyone; we have a very long waiting list.

3.2. Who do you think is benefiting from the Center? *[Researcher probe if the Center is benefiting people from all regions, social economic status, ethnic or religion etc.]*

There are no limits; anyone who comes has access to care regardless of the region. We hope that in the future each region will take care of its patients to avoid geographical inaccessibility.

3.3. Why do you think the ART Center is mostly benefiting these people?

Because the center's indicators in terms of success statistics or care are positive.

Care isn't just about pregnancy; it's about the approach, the care provided even when someone leaves with a diagnosis of infertility, for example. The couple receives dignified care, respecting their dignity by saying, "Here's the diagnosis; you left with this result

This adds value. A third of the IVF cycles are successful, and this falls within the norms for conventional IVF.

3.4. In your view, which factors are contributing to the Center having an impact? How do these factors cause the Centre to have an effect? In what way? *[Probe Mechanisms]*

Yes, primarily I will return to HR. It's the team working on-site who believed in this work, in this project. They are dynamic in maintaining, developing, and doing their best to establish their place nationally, despite the rapidly changing environment, whether due to COVID or the organization of the healthcare system. Despite all this, the team was there on-site, despite the difficult times. Resources are the most important factor in establishing and maintaining continuity because there is a team that believes and has demonstrated that it can be done and has enabled us to maintain operations. So it's a team effort.

3.5. In your view, what factors can potentially prevent provision of fertility care services for men and women with infertility at the Center? What should be done about these issues?

The two elements that can hinder progress and that we are working on are maintaining the HR of the team. For 12 years, there has been an aging of the teams. Each time, we have introduced new assignments of people whom we have taken under our wing to train. For biologists and clinicians, this is an important factor. There is also the equipment, which needs renewal. These are the two elements that can disrupt progress and that we are working on to maintain it. On the financial side, we will have to readjust the costs at our place so that we remain within a balanced budget because we must meet eligibility requirements, i.e., we are given funds, we must have revenue. All of this must not be done at the expense of individuals. This is the introduction of the AMO (Compulsory Health Insurance). Even the solidarity-based AMO must invest in the care of assisted reproductive technology (ART). Also, the growing media and consumables are dependent on companies, and these are things we don't control. We should promote the companies that supply these products to ensure quality. This is also an important factor that could hinder the center's work.

3.6. Compared to existing need, do you think that the Center is meeting the needs of fertility care in Morocco? What else should be done?*[Researcher to probe further, researcher might point out that the Center is in a large city. How does this affect rural population? Are the number of ART Centers adequate?]*

The center addresses the infertility needs of the population, but over these 10+ years of operation, we realized that we cannot meet the needs of all of Morocco. We have a long waiting list, which is why it is urgent to have other centers in each region. This is an emergency, which is why in each of the 12 regions of Morocco, there should be an organization for infertility care. All of this is outlined in the document to reduce geographical and financial inaccessibility. That is to say, there is a whole organizational proposal that each university hospital should have a fertility center, and each region should have a dedicated infertility care pathway, just like cancer care, pregnancy care, and STIs. This affects the rural population, so there needs to be a dedicated pathway.

3.7. In your opinion, does the ART Center play any other role in fertility care provision? Which one? [researcher probe referrals or training of health professionals]
The role of training, research, and expertise. Moreover, for training, the first residents trained in IVF were a few residents who came from the maternity ward, that is, residents from the Rabat University Hospital. We couldn't take all the gynecology-obstetrics residents from the Rabat University Hospital; that's why it's necessary to establish more centers. Training will take place abroad for basic training. It was the IVF center at the Orangers Maternity Hospital that introduced the infertility and IVF component into the basic training of residents. For other public centers, there was a transfer; centers in Marrakech, Tangier, and Oujda benefited to varying degrees from this center, and that was the initial idea of the project. It is a reference center.

3.8. What are the reasons why the development and implementation of the ART Center has been successful or not?

1. The first was the support of Moroccan institutions, notably the FMPR, the Rabat University Hospital, and the leadership of the maternity ward, which contributed to its success.

2. The center's team, a select group, played an extraordinary role in executing and implementing the project, taking ownership of it.

These are the main factors that contributed to the success of this work.

4. Perspectives on learning from Morocco to other countries

I would like to finish off by asking you about what has been learnt in Morocco and how it can be used to assist other counties to start provision of fertility care in public hospitals.

4.1. In your opinion what would be the benefits, if any, to the implementation of a publicly funded ART Center in another country?

The first advantage is providing care to an infertile population, which is significant throughout the country, so that they can have access within the country. When there is no IVF center in the country, only a small category of infertile couples will have to travel to other countries for procedures, a colossal loss for the country. That's why the public authorities will invest to keep infertile couples locally. This is the first element of investment for what the maternity ward has done for other countries. The maternity ward has been requested by several countries; it has been requested by Mauritania, Senegal, Benin, and Côte d'Ivoire to transfer and support

infertility. And the maternity ward has played a role in the MENA region through its status as a WHO Collaborating Center to share the experience of 22 countries in the MENA region.

4.2. In your opinion, are there obstacles to the development of public ART Center? If so, which ones and how can these be overcome?

The first obstacle is the lack of awareness among policymakers that this is a medical condition requiring investment and implementation. If a country's Ministry of Health recognizes that it needs an ART center because its population is suffering, but doesn't recognize this and take the necessary steps to organize a center, then it would be an obstacle.

4.3. What other considerations do you think should be taken into account if/when introducing such public ART Centers in other low- and middle-income countries?

If we want to introduce an ART center in other countries, we must consider the constraints of introducing a cutting-edge technology that requires clinical expertise which we can address, but the biological laboratory skills are very difficult and require significant investment. The key consideration is to bring these two technologies together in certain countries, and it's not just about introducing the technology itself, but also everything that accompanies it, i.e., For clinicians, it's fertility awareness; for biologists, it's the equipment. There must be companies that handle the equipment, that can import it through purchasing, and that provide maintenance for this equipment.

The growing media need to be adapted to the environment of each country. In some countries, it's 40°C with high humidity, and the consumables must not be destroyed by the heat. All of these factors must be considered beforehand in each country.

Thank you very much, that is the end of the interview. I will stop the recording now.
