# Supplementary material for: A realist evaluation of the development, implementation and outcomes of the first public ART Centre in Morocco
Source: PLOS Glob Public Health. 2026 Apr 20;6(4):e0005318. doi: 10.1371/journal.pgph.0005318 (PMC13094999; doi:10.1371/journal.pgph.0005318)
Supplement: S2 Data — (ZIP) [file pgph.0005318.s013.zip › S2_Data_Transcriptions_in _English/S4.pdf]

Interview Guide for Policy Makers

Participant Code NUMBER: ___________S4

1. General Landscape and Context of Fertility in Morocco

First, I would like to start by asking you a few questions on the general situation in Morocco around infertility.

1.1. How is Morocco as a country addressing infertility?

It's a very vague question; you have to look at the past. These are initiatives from the private sector. Morocco's policy and the Ministry of Health's policy on couple health regarding infertility is primarily focused on combating hypernatalism, and therefore state measures are geared towards limiting birth rates rather than helping infertile couples. That's why only initiatives from the private sector are able to meet this demand.

1.2. Do we have a national policy or a strategic plan to address infertility in Morocco?

As far as I know, no, there is no national policy or strategic plan. If there is a plan, it is not acknowledged, it is not declared. In any case, what we see is still the establishment of a public center for the AMP, but a strategic plan that will talk about a plan, no.

1.3. Which policies and laws regulate fertility care and assisted reproduction in Morocco?

I'm not going to talk about policies but about laws; there's only one law, Law 47-14, which governs this medical specialty based on medical data. It's a medical specialty; it's governed by Law 131-13 and Law 47-14 for the clinical aspect; for the biological aspect, it's governed by Law 12-01 and Law 47-14.

1.4. Is there a national registry and licensing body for fertility care and assisted reproduction?

Not at this time.

1.5. Is infertility included as an essential component of Sexual and Reproductive Health and Rights (SRHR) policy and services in Morocco?

I'm not aware of that.

1.6. Do you think that fertility care is important in our setting and why?

It's a medical condition like any other; you have to see the journey and struggle of infertile couples and the social tragedies that follow. Yes, they are important, and the infertility rate in Morocco will continue to climb; we will reach the rates of Western and developed countries.

2. Setting up of the Public ART Center

I would now like to talk with you about the first Public ARTC Center at XX which you may have been involved in setting up or have at least heard about.

2.1. What was your role in the implementation of this first public ART center in Morocco?

It's just to allow the law to work; we participated in the training of certain practitioners from public centers within the framework of the learned society.

2.2. What was the situation like before the first public ART Center was put in place? How were couples accessing services? What problem did it solve?

Infertile couples who couldn't afford it couldn't access this treatment. And now that's changed; it's given hope to couples who don't have much money.

2.3. What steps were taken to ensure that services could be started at the ART Center? [please elaborate]

We took action by setting up a dedicated center for this purpose, which was included in the construction phase from the outset, respecting European standards for equipment quality and training for the practicing staff.

2.4. What policies and regulations were needed to ensure that ART provision was possible at the Center? [please elaborate]

Ongoing staff training will facilitate the ordering and delivery process for consumables and equipment maintenance contracts, the establishment of a dedicated administrative and computerized registry service, and ensure that the infertile couples' care pathway is properly managed. The risk is that in a public center, the pathway will be lost and diverted to other departments. That's why National Agency of medical insurance is working on a care framework and developing a treatment protocol.

2.5. What are some of the actions and measures that were needed in order to enable the provision of fertility services in Public ART Centers? *[Researcher to probe what action needed to take place in relation to i) Pricing of ART treatments, ii) health coverage of infertility treatments, iii) Marketing Authorization and Registration of culture media and medical devices, iv) standardization of public ART centers, v) development of infertility management guideline, vi) Integration of infertility in Health Plan, and v) Application decrees of Bill N° 47-14 on ART]*

The investment covers the location, infrastructure, equipment, and training.

2.6. What were the key investments in the health system infrastructure that needed to be made during the setting up and implementation of the Center? *[Researcher to probe what action needed to take place in relation to i)service delivery, ii) health workers iii) health records iv) infertility medicines and equipment) management and leadership and vi) financing and subsidization]*

2.7. How is the setting up of the provision of public fertility care at the Public ART Center financed? Please elaborate.

The university hospital is handling that.

3. Contributions and Outcomes of the Public ART Center

I would like now to focus on events since first Public ART Center was set up.

3.1. What difference do you think the ART Center has made to people with infertility? Why so?

The reduction in prices for assisted reproductive technology (ART) attempts has allowed some couples to have access to this care.

3.2. Who do you think is benefiting from the Center? *[Researcher probe if the Center is benefiting people from all regions, social economic status, ethnic or religion etc.]*

all layers, all regions I suppose,

3.3. Why do you think the ART Center is mostly benefiting these people?

Because it's an observation made by people who have access to it and have not been refused acceptance.

3.4. In your view, which factors are contributing to the Center having an impact? How do these factors cause the Centre to have an effect? In what way? *[Probe Mechanisms]*

It's simply a matter of including ART in the healthcare package; if we do that, all people who benefit from compulsory health insurance (AMO) will benefit from this coverage, whether they are covered by AMO or CNOPS.

3.5. In your view, what factors can potentially prevent provision of fertility care services for men and women with infertility at the Center? What should be done about these issues?

Factors that can hinder administrative procedures include: if they are too cumbersome, if appointments are far away, or if there is a mismatch between services and the human resources available at the center. This will lead to longer appointment times and even cause problems.

If there is a mismatch between supply and demand, even if there are 5 centers, is that enough?

On the other hand, if there is a health policy involving the outsourcing of services within the framework of public-private partnerships, which officials boast about and declare in all interviews, if they were willing to use public-private partnerships, it would be a good example.

3.6. Compared to existing need, do you think that the Center is meeting the needs of fertility care in Morocco? What else should be done?*[Researcher to probe further, researcher might point out that the Center is in a large city. How does this affect rural population? Are the number of ART Centers adequate?]*

I don't think the number of cases is very significant; that's where public-private partnerships can play a role in shortening appointments. Agreements and conventions are necessary.

3.7. In your opinion, does the ART Center play any other role in fertility care provision? Which one? [researcher probe referrals or training of health professionals]

Yes, it's another role of raising awareness and participation in educating the Moroccan population. Trying to raise the level doesn't mean ending up with cases that drag on for years between the fqih, laachab, etc., and that will improve the quality of care. Sometimes it's a medical problem, and there's a solution to this problem through competent medical care.

3.8. What are the reasons why the development and implementation of the ART Center has been successful or not?

Success lies in meeting the needs of certain couples who travel with children; the information that is shared, the level of awareness is higher, and knowledge is updated while everyone benefits.

4. Perspectives on learning from Morocco to other countries

I would like to finish off by asking you about what has been learnt in Morocco and how it can be used to assist other counties to start provision of fertility care in public hospitals.

4.1. In your opinion what would be the benefits, if any, to the implementation of a publicly funded ART Center in another country?

If Morocco follows the policy outlined by His Majesty Mohammed VI, it will be a South-South cooperation, and that's a win-win situation. As in other areas, Morocco can play a leading role in disseminating information and providing care for infertile couples in their countries of origin.

Similar to what is done in other areas, this is a South-South cooperation.

4.2. In your opinion, are there obstacles to the development of public ART Center? If so, which ones and how can these be overcome?

We just need to look at the trade agreements between the two countries, the trade legislation, and the reciprocal legislation, and the financing, which is a major issue that needs to be thoroughly discussed and carefully planned.

4.3. What other considerations do you think should be taken into account if/when introducing such public ART Centers in other low- and middle-income countries?

We're talking about this right now, it's a cooperation; Morocco has been ready to help low-income African countries, among others. It's within the framework of exchanges and cooperation; Morocco has always been a pioneer in this field, and why not extend it to the medical field and why not extend it to assisted reproductive technology (ART), which is part of it.

Thank you very much, that is the end of the interview. I will stop
