# Supplementary material for: A realist evaluation of the development, implementation and outcomes of the first public ART Centre in Morocco
Source: PLOS Glob Public Health. 2026 Apr 20;6(4):e0005318. doi: 10.1371/journal.pgph.0005318 (PMC13094999; doi:10.1371/journal.pgph.0005318)
Supplement: S2 Data — (ZIP) [file pgph.0005318.s013.zip › S2_Data_Transcriptions_in _English/S5.pdf]

Interview Guide for Policy Makers

Participant Code NUMBER: __________S5

1. General Landscape and Context of Fertility in Morocco

First, I would like to start by asking you a few questions on the general situation in Morocco around infertility.

On this question, I will answer you honestly. I personally ask myself the same question: Is it an illness or simply a desire to have children for a minority of people?

In my opinion, the healthcare system does not recognize infertility as a "pathology" or a "public health problem."

During the implementation of the Compulsory Health Insurance, I was at ANAM (National Agency for Health Insurance) and I witnessed that no one at the time mentioned infertility as a component of the service package to be covered, which demonstrates that it was not among the healthcare system's priorities.

For the sake of financial balance, there is a prioritization process, and infertility was not addressed.

2. Setting up of the Public ART Center

I would now like to talk with you about the first Public ART Center which you may have been involved in setting up or have at least heard about.

I didn't participate in the center's creation, but I know about it and I truly believe it was a first in Morocco, as previously only the private sector had offered services to infertile couples.

Regarding the difficulties encountered by the center, given that it is a public institution subject to public procurement regulations, I think the availability of medications and medical devices is sometimes hampered by the monopoly of pharmaceutical companies, which prefer to invest in countries where services are well-established, demand is high, and where medications are covered by health insurance.

The law of the market is supply and demand; The nature of the medications (Hormones), the lack of generics, fixed prices with low demand (few public centers) – all these factors influence the availability of the medications and medical devices necessary for the proper functioning of the center. This is due to the low attractiveness of laboratories.

Morocco is one of the most developed countries in the pharmaceutical industry and in the organization and regulation of the supply system for medications and pharmaceutical products. And we are proud of this progress.

Faced with this situation, it is imperative to have the involvement of political decision-makers and openness to the international community.

Political commitment is crucial; if the state intervenes, everything becomes possible. There is the fast-track process for the registration of medications and pharmaceutical products, and there are numerous examples of the intervention of political decision-makers in the success of several programs, such as vaccination, tuberculosis, and HIV. Without political commitment to these health programs, they would have been doomed to failure. Therefore, I believe that state intervention is essential in certain programs, as it remains the guarantor of this population's right to health.

3. Contributions and Outcomes of the Public ART Center

I would like now to focus on events since first Public ART Center was set up.

In my opinion, this center has contributed, firstly, to raising awareness that this is a public health issue, because many people didn't believe it, including myself.

Secondly, access to infertility services remains too expensive and impossible for people from low and middle socioeconomic backgrounds. Here we have a structure that offers hope to this group and breaks the idea that it's solely a private matter. Therefore, this center brings significant added value to the issue of infertility in our country.

And the creation of this center stems from the leadership of our Professor B, who conducted this initial project in Morocco, and it is through advocacy and the commitment of people convinced of the cause that things are coming to fruition. Honestly, I think the recent integration of certain infertility treatment medications is one of the impacts of this public center, and this is just the beginning and it must continue to expand medical coverage to other medications and even treatment techniques, for example, the number of IVF cycles.

4. Perspectives on learning from Morocco to other countries

I would like to finish off by asking you about what has been learnt in Morocco and how it can be used to assist other counties to start provision of fertility care in public hospitals.

Morocco is a country that has made significant progress in recent decades in the pharmaceutical industry and in the quality control of medicines and medical devices, as well as in universal health coverage. I can truly say that we are very proud of this progress.

Regarding infertility, it is only in the last decade that we have begun to see the impact of major advocacy efforts on infertility, with legislation and the creation of more public and private centers. However, there is still work to be done, and this must be a priority issue with the commitment of policymakers to regulate the pharmaceutical market for the benefit of the population.

Thank you very much, that is the end of the interview. I will stop
