# Supplementary material for: A realist evaluation of the development, implementation and outcomes of the first public ART Centre in Morocco
Source: PLOS Glob Public Health. 2026 Apr 20;6(4):e0005318. doi: 10.1371/journal.pgph.0005318 (PMC13094999; doi:10.1371/journal.pgph.0005318)
Supplement: Attachment — Submitted filename: Response to reviewers.pdf [file pgph.0005318.s017.pdf]

To the editor,

Plos Global Public Health

February , 2026

Dear Editor

Re: Response to reviewers

We are pleased to attach our point-by point response to reviewers' queries. We thank the reviewers for taking the time to review our work. All your comments were incorporated accordingly in the manuscript text. All the changes are highlighted in the text.

Thank you for considering our work.

Point by point responses:

Figures : Figure file has been processed using the PLOS NAAS tool as recommended. The downloaded NAAS-validated TIFF file has been uploaded with the revised submission to ensure compliance with PLOS formatting and resolution requirements.

REVIEWER 1:

1. Most of the comments have been included as possible. Word limit needs to be checked to make it concise. Few grammatical checks required.

Response:

We thank the reviewer for this positive assessment and for acknowledging that previous comments have been adequately addressed.

In response to the recommendation on conciseness : the manuscript has been carefully edited to reduce redundancy and improve clarity, repetitive statements in the introduction and discussion sections were streamlined, some elements were condensed without altering the meaning.

The manuscript has also undergone thorough grammatical and stylistic editing. Minor typographical and syntactic errors were corrected throughout the text. Sentence structures were simplified where necessary to enhance readability.

Changes to the text are highlighted in the manuscript.
