# Supplementary material for: A realist evaluation of the development, implementation and outcomes of the first public ART Centre in Morocco
Source: PLOS Glob Public Health. 2026 Apr 20;6(4):e0005318. doi: 10.1371/journal.pgph.0005318 (PMC13094999; doi:10.1371/journal.pgph.0005318)
Supplement: Attachment — Submitted filename: Plos Comments.docx [file pgph.0005318.s014.docx]

1. Congratulations to the authors for their hard work. It is good work done of establishing the ART center in Morocco and a good role model for other LMICs.

2. The qualitative data is described in the manuscript however the details of quantitative data w.r.t services availed, success rates is lacking. It is mentioned in the beginning that quantitative data will be described.

3. Please check with the word limit of the journal for qualitative manuscript. Description can be made concise

4. In discussion please mention about models of care for infertility in LMICs, if published literature is available

5. remove typical from line 126 and 128

6. mention the details of ART services provided at the center

7. 129 line onwards As of the time of study used very frequently .

Comment: Minor Revision. Such Models should be published
