# Supplementary material for: A realist evaluation of the development, implementation and outcomes of the first public ART Centre in Morocco
Source: PLOS Glob Public Health. 2026 Apr 20;6(4):e0005318. doi: 10.1371/journal.pgph.0005318 (PMC13094999; doi:10.1371/journal.pgph.0005318)
Supplement: Attachment — Submitted filename: Response to reviewers.docx [file pgph.0005318.s016.docx]

To the editor,

Plos Global Public Health

November , 2025

Dear Editor

Re: Response to reviewers

We are pleased to attach our point-by point response to reviewers’ queries. We thank the reviewers for taking the time to review our work. All your comments were incorporated accordingly in the manuscript text. All the changes are highlighted in the text.

Thank you for considering our work.

Point by point responses:

**1. Ethics File Upload** : We note that your Supporting Information files contain Ethical Approval documents. Please note that PLOS does not publish Ethical Approval forms as Supporting Information files, and it is only required that ethical approval is reported in the body of the manuscript. Therefore, we ask that you please resubmit these documents as Other files, which are not intended for publication.

**Response:**
We have re-uploaded the Ethics Approval (AF 96-22 and its English translation) under "Other files".

**2. Data Availability**: “All PLOS journals now require all data underlying the findings described in their manuscript to be freely available…”

**Response**:
We appreciate this clarification. We have prepared anonymized summary data used in the manuscript, which we have now uploaded as Supporting Information S12 Excel file and S13 Zip. We have also updated the Data Availability Statement accordingly: “Anonymized data used for this study are available in the Supporting Information”

- « Thank you for submitting your underlying dataset in the file titled “Supporting information.rar”. We note that this data set consists of interview transcripts. Before we proceed, can you please confirm that all participants gave consent for their interview transcripts to be published? »

**Response** : All participants provided consent for their interview transcripts to be published. All transcripts have been fully de-identified, and no identifying information (names, initials, addresses, specific dates, or small-group identifiers) remains. Also the transcripts are provided in english as per the editors request.

**3. Financial Disclosure:** Please provide a detailed online Financial Disclosure statement. This is published with the article. It must therefore be completed in full sentences and contain the exact wording you wish to be published.

**Response:** This work was supported by the UNDP–UNFPA–UNICEF–WHO–World Bank Special Programme of Research, Development and Research Training in Human Reproduction (HRP), a co-sponsored programme executed by the World Health Organization. Four co-authors (GM, HH, KG, JK) are staff members of the World Health Organization. The views expressed in this article are those of the authors and do not necessarily represent the views, decisions, or policies of the World Health Organization. The funders had no additional role in data collection and analysis, or the decision to publish.

**4. Funding Information :** Please ensure that the funders and grant numbers match between the Financial Disclosure field and the Funding Information tab in your submission form. Note that the funders must be provided in the same order in both places as well.

**Response :** the same text (and order) in both the Financial Disclosure, and the Funding Information was respected.

**5. Manuscript file format :** We uploades the manuscript as .docx.
*No LaTeX file used; the manuscript was prepared in Word.*

**6. Figures :** Figures were exported as .tif

**7. Supporting Information :** the files that were as .rar were replaced with a .zip file.

**REVIEWER 1:**

**1.** Congratulations to the authors for their hard work. It is good work done of establishing the ART centre in Morocco and a good role model for other LMICs.

**Response:** Thank you for recognizing our work, this is greatly helpful to us.

**2.** The qualitative data is described in the manuscript however the details of quantitative data w.r.t services availed, success rates is lacking. It is mentioned in the beginning that quantitative data will be described.

**Response:** Thank you. We have revised the manuscript to include quantitative data in the Results section.

Tables 09-11 have been added as Supporting Information for completeness. Changes to the text are highlighted in the manuscript.

**3.** Please check with the word limit of the journal for qualitative manuscript. Description can be made concise

**Response :**Thank you for this helpful comment. As per the PLOS Global Public Health author guidelines, there is no fixed word limit for qualitative manuscripts. However, we agree that clarity and conciseness are important. In response, we have carefully reviewed the manuscript to reduce redundancy while preserving the richness and integrity of the qualitative data.

Changes to the text are highlighted in the manuscript.

**4.** In discussion please mention about models of care for infertility in LMICs, if published literature is available

**Response :**Thank you for the valuable suggestion.We have included ( in the discussion section) examples of ART models from other LMICs, such as South Africa, Gambia, and Rwanda. Changes to the text are highlighted in the manuscript.

**5.** Remove typical from line 126 and 128

**Response :**We have removed the word “typical” as suggested.

**6.** Mention the details of ART services provided at the centre

**Response :**We have expanded the « Nature of services provided at the ART centre » section to describe the services. A Figure, showing the couples’ care pathway, have been added as Supporting Information 2 for clarity. Changes to the text are highlighted in the manuscript.

**7.** 129 line onwards As of the time of study used very frequently .

**Response :** We have revised the phrasing throughout the manuscript to eliminate redundancy while retaining chronological accuracy.

Comment: Minor Revision. Such Models should be published

**REVIEWER 2:**

**1.** Does this manuscript meet PLOS Global Public Health’s publication criteria?
Response: No

My feedback:
The manuscript number Manuscript Number PGPH-D-25-01503 presents a promising model of public sector ART service delivery in Morocco and reflects a rare example of health system innovation in a traditionally privatized domain. However, it lacks critical elements that would allow it to serve as a model for replication, such as disaggregated outcomes, internal/external evaluations, adaptation learnings from the Belgian model, and structured quality assurance indicators. Therefore, while methodologically it holds promise, it is not yet ready for publication in its current form.

**Response :**We thank the reviewer for highlighting these critical aspects and for recognizing the potential of the Moroccan ART centre as a model for public sector service delivery in LMICs.

We have revised the manuscript to provide more details on clinical outcomes, describe internal monitoring mechanisms used at the ART centre, outline the key components of the Belgian model that were adapted locally, and include reference to quality assurance practices.

**2.** Has the statistical analysis been performed appropriately and rigorously?
Response: No
My feedback:
No statistical or quantitative evaluation of program impact is presented. The paper mentions 2,495 beneficiaries (Line 52) but does not clarify whether this refers to individuals or couples, nor does it provide clinical success rates (pregnancy, live birth, etc.). Time-trend data and baseline burden are entirely absent.

**Response :**We thank the reviewer for this important observation. We have clarified throughout the manuscript (in the Methods and Clinical Outcomes sections) that the 2,495 refers to couples, not individual patients.

We have added a « Clinical Outcomes » section in the Results section and Tables 09-11 have been added as Supporting Information with more detailed data including:

- The number and proportion of IVF, FET, IUI, and ovulation induction cycles performed;
- Pregnancy rates, Live birth rates ;
- Number of complications (none reported).

We agree that time-trend and baseline burden data would enhance the program evaluation, however such data were not available across the 2013–2022 period due to limitations in the national health information systems and the absence of an ART services registry in Morocco. We have acknowledged this limitation explicitly in the revised manuscript and emphasized the need for a national ART registry going forward (results and discussion sections).

**3.** Have the authors made all data underlying the findings in their manuscript fully available?
Response: Unclear
My feedback:
No links to raw data, transcripts, or internal monitoring systems have been provided. There is insufficient transparency in how the findings (especially Table 3) were generated.

**Response :** Thank you for this important observation**.** We have prepared de-identified summary data used in the manuscript and uploaded them as **Supporting Information**.
In regard to the construction of Table 3 (Context–Mechanism–Outcome configurations) we have expended on how the findings were generated in the Method section. Changes to the text are highlighted in the manuscript.

**4.** Is the manuscript presented in an intelligible fashion and written in standard English?
Response: Yes, with revisions needed
My feedback:
- While the language is generally clear, specific terminology such as “official inauguration” (Line 381) needs replacement with “optimally functional” to better reflect when the centre became fully equipped to offer services professionally. This also implicitly conveys the time needed when LMICs lack a formal enabling policy.

**Response :**Thank you for this thoughtful observation. We acknowledge the importance of distinguishing between the formal inauguration and the point at which the centre became fully operational. In our context, the term “official inauguration” refers specifically to the ceremonial event held in May 2016, attended by the Belgian Ambassador and the Moroccan Minister of Health. However, the centre became optimally functional much earlier, in 2014, when the first cycles were initiated and full services were made available.

To avoid confusion, we have revised the manuscript using “ceremonial inauguration event” instead of “official inauguration”. Changes to the text are highlighted in the manuscript.

- The structure also suffers from repetition in the CMO framework across sections.
While the manuscript refers to the realist evaluation model, the connection between context, mechanism, and outcomes is not consistently or clearly demonstrated. Authors should strengthen how these three elements interact across different domains in Table 3 and the results.

**Response:** Thank you for your observation. We added a diagram (Figure 1) that maps the themes and subtheme explicitly connecting context, mechanisms, and outcomes, thus, emphasizing that these factors interact and reinforce each other.

**5. Review Comments to the Author:**
**Strengths:**
The paper captures a policy breakthrough in making ART services publicly accessible in Morocco, a country with a pluralistic health system.
The timeline from concept (2008) to operationalization (2013) reflects sustained effort and partnership with Belgian clinical institutions (Lines 398–404).
The use of realist evaluation offers valuable theoretical framing.
Table 3 is well-placed under results (Line 231) and integrates institutional and societal enablers.

**Response :**We thank the reviewer for recognizing the strengths of our study. We appreciate the encouraging feedback.

**Areas for Improvement:
Ambiguity in Sample Size and Units**
Line 52: Mentions 2,495 “patients,” and Line 477 states “more than 2,000 people.” In ART, the unit is typically “couples.” Clarification is needed. This has implications for calculating success rates per treatment cycle, assessing access equity, and establishing appropriate denominators for ART program performance.

**Response :**We thank the reviewer for this helpful observation. We have revised the manuscript to consistently refer to the unit of analysis as “couples”. We have clarified that the 2,495 refers to the number of couples who received services at the public ART centre between 2013 and 2022. This clarification has been made throughout the manuscript.

**Gender Dynamics**
Line 306–307 notes that male infertility was frequently observed at the center, helping raise awareness on a previously socially invisible issue. This is a critical gender-sensitive insight that deserves greater visibility in the Results or Discussion section. The manuscript could elaborate on whether this influenced the design of counseling protocols, community messaging, or clinical engagement strategies. A policy reflection on the role of public centers in reshaping gendered narratives of infertility would add depth and align with WHO's gender equity lens.

**Response :** We thank the reviewer for highlighting the importance of gender dynamics in infertility care. We have expanded the Methods section (Line 175 - 187) to elaborate on the Centre’s gender-sensitive practices.All stages of care were conducted with both partners. Even in cases where the infertility cause was female, male partners were actively involved in counselling and treatment planning. This couple-centered approach permitted shared decision-making, reduced stigma surrounding male infertility and helped a better understanding of infertility as a shared health concern, we have added a discussion about it.

**Inadequate Outcome Reporting**
Lines 52–53: Mention the number of beneficiaries but omit clinical and programmatic outcomes such as live birth rate, age-specific success rates, number of treatment cycles, patient follow-up, and dropouts. This is a major limitation for a paper claiming success of a public sector ART model. Without such outcomes, replication, scalability, and cost-effectiveness analysis remain speculative.

**Response :**Thank you for this important observation. We have added available quantitative data to the Results section and Tables 09-11 have been added as Supporting Information with more detailed data. While our dataset does not yet include comprehensive age-specific success rates, we recognize these as critical metrics for future evaluations and have noted them as limitations.

**Missing Baseline Burden and Trends
-**No reference is made to the infertility burden in Morocco, either from Demographic Health Surveys, national estimates, or global data sources (such as WHO’s 2023 report on global infertility burden). Absence of such trend data (2008–2021) makes it difficult to assess the program’s reach or relevance. The lack of historical context and service coverage dilutes the paper’s utility for policy and planning.

**Response :** Thank you for highlighting this point. We have now revised the manuscript to include references to the global burden of infertility as reported by the WHO 2023 global infertility estimates, as well as national estimates, including recent findings from Morocco's National Population and Family Health Survey (ENPSF). These have been added in the introduction section.

- Especially important is the absence of dropout rates, failed cycles, and follow-up completion. These are core indicators for ART program performance and their omission undermines claims of effectiveness.

**Response :** We appreciate the reviewer’s important observation. We have clarified in the manuscript (Results section) that all observed treatment dropouts occurred after initial consultations and diagnostic investigations, but prior to the start of any ART cycle. Once treatment commenced, no couples dropped out during ovarian stimulation, oocyte retrieval, or embryo transfer, which we attribute to the bundled payment model that encourages completion of the full treatment cycle. More detailed data (Live birth rates and cancellation rates) were incorporated in Tables 09-11 as Supporting Information.

**Inconsistencies in Timeline of Operations**
Line 381: The term “official inauguration” is from the article and may be retained, but the review suggests clarifying when the center became optimally functional—that is, professionally staffed, regulated, and delivering quality-assured services. This will avoid reader misperception that initial services were makeshift or informal and more accurately reflect the delay caused by the absence of enabling policies in LMIC contexts.

**Response :** We thank the reviewer for this valuable suggestion. We have clarified the timeline in the manuscript to distinguish between the ceremonial inauguration event of the ART Centre in 2016 and the period during which it became optimally functional (fully staffed and operating under approved protocols). Changes to the text are highlighted in the manuscript.

**Replication and Adaptation Unclear**
Lines 398–404: The manuscript notes the replication of Belgian standards, but without clarity on contextual adaptations. What clinical protocols, eligibility criteria, or service delivery components were localized? Were any services modified due to sociocultural or systemic differences? These questions are central to understanding how LMICs can replicate such a model with adaptation. The paper should offer examples of which parts of the Belgian model were directly adopted and what was modified to suit the Moroccan context.

**Response :** We appreciate this important point. We have expanded the Methods section to clarify which Belgian standards were directly adopted and which were adapted to fit the Moroccan context. Changes to the text are highlighted in the manuscript.

**WHO Health System Pillars**
Lines 107–108: The authors refer to WHO’s six health system building blocks but do not systematically address them. Key gaps include:

**Response :** We thank the reviewer for this important observation. We have revised the manuscript to explicitly present relevant information for each block. Changes to the text are highlighted in the manuscript.

Health Workforce: No mention of staff training, cadre mix, retention mechanisms, or capacity building.

**Response :**We added details in the methods section on staff training (specialized training in infertility for gynecologists, biologists, and nurses), cadre mix, and retention strategies.

Health Information Systems: The existence of an HMIS is mentioned, but no data on indicators collected, gender disaggregation, or system use is provided.

**Response :**We have included in the methods section a description of the Gyneco 2000 software including the types of data collected. We also described how gender-disaggregated indicators are recorded, and the use of the system.

Leadership and Governance: Decision-making, accountability systems, or institutional coordination (between MoH, local governments, and partners) is not discussed.

**Response :**We have revised the manuscript adding the decision-making structures, the coordination between the ART centre, and other institutions and the role of the centre in drafting ART regulations.

Health Financing: Cost per ART cycle, proportion of out-of-pocket expenditure, insurance mechanisms, or state subsidies are not documented, despite being critical to assess equity. The manuscript should also clarify whether any cost transparency measures were in place, such as fee disclosure to patients or standardized billing. This would signal good governance and equity.

**Response :** We have included Table 8 as Supporting Information detailing the costs of ART cycles. We have also clarified that the full amount of treatment costs is borne directly by the couples, as there is no insurance coverage or state subsidy for ART services in Morocco.We also clarified that patients received clear information about standardized fees prior to treatment initiation.

**No Mention of Quality Improvement (QI) or External Evaluation**
There is no reference to any internal audit, clinical review cycle, patient feedback mechanism, or third-party evaluation. In a field as sensitive as infertility and setting up something greenfield project- quality assurance is central to patient safety, ethical practice, and public confidence. Future revisions should specify whether QI processes (e.g., treatment success audits, stock-out surveillance, complication monitoring) were instituted.

**Response :** Thank you for highlighting this important point. We have added details on internal quality assurance at the ART Centre, including standard operating procedures (SOPs), clinical protocols, and a secure health information system. Two internal evaluation studies (2018) on sperm concentration quality control and retrospective ART outcome assessment support ongoing quality improvement. While no formal external evaluation occurred, continued collaboration with partners provides informal benchmarking. Changes to the text are highlighted in the manuscript.

**Impact Beyond Access – Results Underreported**
The manuscript restricts its results to availability and access, ignoring broader systemic and societal impact. Suggested additions include:
Program influence on policy dialogue and normalization of ART as a public health service
Regulatory effects on pharmaceutical and consumable pricing or availability
Testimonials showing how the program affected patients’ emotional wellbeing, confidence in public health systems, or reduced stigma. These dimensions are especially important in infertility care.

**Response :** Thank you for your observations on the policy impacts of the ART centre. We would like to highlight that several of these points are already addressed in the Result section, particularly in the Enabling policy and legal environment section. For example, the manuscript elaborates on the advocacy efforts with the Ministry of Health and the National Health Insurance Agency that led to the registration of ART supplies and drugs. It also details the enactment of Law 47-14 in 2019, which regulates ART practice in Morocco, and how the ART centre played a pioneering role in influencing national reproductive health strategies and expanding services to other university hospitals.

In regard to patients’ emotional wellbeing, and confidence in public health systems, we added a patient testimonial showcasing the psychosocial outcomes.

Changes to the text are highlighted in the manuscript.

**Policy Recommendations Too Generic**
Line 576: Recommending “similar centres” does not account for resource constraints, regional needs, or institutional capacity. Consider suggesting embedded ART units within existing multispecialty hospitals with teleconsultation support, tiered referral protocols, or regional ART hubs linked to PHCs.

**Response :** Thank you for this constructive suggestion. We have revised the recommendations section to move beyond the generic proposal of “similar centres.” Specifically, we now highlight the importance of embedding ART units within existing multispecialty teaching hospitals to optimize human resources and infrastructure. We suggest using a hub-and-spoke model, where regional ART hubs provide advanced care and primary health centres (‘spokes’) deliver first-line infertility services. Other recommendations are also detailed in Table 5. Changes to the text are highlighted in the manuscript.

**Professional Organizations & Multi-Stakeholder Voice**
Line 84: Absence of public sector models is noted but not analyzed. There is no mention of how the initiative engaged (or did not engage) professional bodies like gynecology societies, community representatives, or pharmacists. The governance and advocacy ecosystem is therefore underexplored.

**Response :** We thank the reviewer for this comment. While the initiative did not engage professional bodies, community representatives, or pharmacists, it was supported by institutional coordination between the Ministry of Health, Mohammed V University, and Ibn Sina University Hospital. We have clarified this in the manuscript to reflect the governance and advocacy ecosystem of the ART centre.

**Terminology**
“Enabling environment” in Table 3 remains vague. Authors should illustrate enabling conditions through actual programmatic measures—such as provider incentives, inclusion of infertility in national policy, mitigation of religious/cultural opposition, and streamlining of supply chains for ART consumables.
It would further help to list concrete operational mechanisms like subsidy mechanisms, task-shifting authorizations, or partnership MOUs with local governments that made this “enabling environment” functionally meaningful.

**Response :** Thank you for your valuable feedback. We have substantially revised the Enabling Policy and Legal Environment section to describe key enabling factors such as the inclusion of infertility care in national reproductive health policies, advocacy efforts leading to the enactment of Law 47-14 regulating ART practice, ongoing work to integrate ART drugs into compulsory health insurance reimbursement schemes, and the critical role of regulatory approvals and supply chain processes for ART consumables.

**Private Sector Monopolies – Missed Opportunity**
The paper misses the opportunity to reflect on how ART services in many LMICs are monopolized by private providers with high out-of-pocket costs and minimal regulation. A public sector entry not only enhances access but challenges traditional market structures. This critical policy insight should be emphasized.

**Response :** We thank the reviewer for highlighting this important dimension. In the revised discussion, we have added a paragraph noting that, prior to the creation of the ART Centre, ART services in Morocco were exclusively provided by the private sector, with high out-of-pocket costs, and minimal regulation. The introduction of a public ART centre disrupted this monopoly by offering more affordable, and regulated care.

**Comparative Reference to France**
While the manuscript notes Belgian collaboration, it could benefit from referencing France’s publicly funded ART system, given linguistic and policy parallels in North Africa. Comparative insights would elevate the manuscript’s relevance to other middle-income countries. France offers a publicly funded, equity-oriented ART program, and its health regulations and social norms are closer to the Maghreb context. Referencing its success could guide policymakers in similar sociocultural regions.

**Response :** We thank the reviewer for this pertinent suggestion. We acknowledge the importance of France’s publicly funded ART system as a potentially relevant comparative model for the Maghreb context.However, the scope of our study was specifically to document the development and implementation of the Moroccan public ART centre in collaboration with Belgian partners, rather than to conduct a comparative analysis of different international models. However we have indicated in the manuscript examples of countries with developed ART provision and financing including France, among others which can be part of international collaborations.
